# POLE2 promotes osteosarcoma progression by enhancing the stability of CD44

**DOI:** 10.1038/s41420-024-01875-x

**Published:** 2024-04-16

**Authors:** Baichuan Wang, Hongzhi Hu, Xiaohui Wang, Zengwu Shao, Deyao Shi, Fashuai Wu, Jianxiang Liu, Zhicai Zhang, Juan Li, Zhidao Xia, Weijian Liu, Qiang Wu

**Affiliations:** 1 Department of Orthopedics, Union Hospital, Tongji Medical College, Huazhong University of Science and Technology, 1277 Jiefang Road, Wuhan, 430022 China; 2 Department of Otorhinolaryngology, Union Hospital, Tongji Medical College, Huazhong University of Science and Technology, Wuhan, 430022 China; 3 Institute of Life Sciences 2, Swansea University Medical School, Singleton Park, Swansea, SA2 8PP UK

**Keywords:** Sarcoma, Cell biology

## Abstract

Osteosarcoma (OS) is the most prevalent primary malignancy of bone in children and adolescents. It is extremely urgent to develop a new therapy for OS. In this study, the GSE14359 chip from the GEO database was used to screen differentially expressed genes in OS. DNA polymerase epsilon 2 (POLE2) was confirmed to overexpress in OS tissues and cell lines by immunohistochemical staining, qPCR and Western blot. Knockdown of POLE2 inhibited the proliferation and migration of OS cells in vitro, as well as the growth of tumors in vivo, while the apoptosis rate was increased. Bioinformatics analysis revealed that CD44 and Rac signaling pathway were the downstream molecule and pathway of POLE2, which were inhibited by knockdown of POLE2. POLE2 reduced the ubiquitination degradation of CD44 by acting on MDM2. Moreover, knockdown of CD44 inhibited the tumor-promoting effects of POLE2 overexpression on OS cells. In conclusion, POLE2 augmented the expression of CD44 via inhibiting MDM2-mediated ubiquitination, and then activated Rac signaling pathway to influence the progression of OS, indicating that POLE2/CD44 might be potential targets for OS treatment.

## Introduction

Osteosarcoma (OS) is the most prevalent aggressive primary malignant bone tumor in children and adolescents [1, 2]. OS originates from primitive transformed mesenchymal cells that can trigger osteoblastic differentiation and produce malignant osteoid. OS typically occurs in the metaphyseal region of long bones, with the distal femur (43%) being the more common location, followed by proximal tibia (23%) and humerus (10%) [3, 4]. Risk factors for OS are reported to include taller stature, above-average birth weight and a history of previous radiotherapy treatment [5].

The latest advancements in OS treatment were initiated in the 1980s when multi-drug chemotherapy was proven to improve overall patient survival as compared to surgery alone [6]. However, there has not been a significant improvement in the treatment regimen in the past decades. OS treatment still relies heavily on tumor resection surgery and non-specific combination chemotherapy, which have significant short-term and long-term toxicity and morbidity [7]. The 5-year survival rate for patients with OS local lesions ranges between 60–80% [8]. Additionally, due to the strong metastatic tendency of OS cells [9], approximately 15–20% of patients have clinically detectable metastases at the time of diagnosis. More than 85% of metastases occur in the lungs, which is also a leading cause of treatment failure and death. Therefore, it is dramatically crucial to develop more effective molecular targeted therapy for OS based on in-depth understanding of the mechanism of OS occurrence and metastasis.

DNA replication is a fundamental cellular process that ensures the delivery of genetic information with accuracy [10]. DNA polymerases exert a vital role in the process of normal DNA replication and repair in vivo [11]. In human, there are 15 DNA polymerases identified, belonging to the A-, B-, X- and Y-families, respectively [11]. DNA replication in eukaryotes necessitates the synergy between 3 DNA complex enzyme B family members (Pols α, δ and ε), among which polymerase epsilon (POLE) is the primary enzyme responsible for the synthesis of the leader strand. POLE plays an essential role in maintaining genetic stability during DNA replication [12, 13], and comprises 4 subunits (A–D). DNA polymerase epsilon 2 (POLE2) is located on chromosome 14q21-q22 and is a POLE B subunit involved in the regulating DNA replication, repair and the cell cycle [14–16]. Moreover, POLE2 is modified by APRIL to affect the proliferation of liver cancer cells [17]. However, the role of POLE2 in human cancers, especially OS, is still largely unknown.

To gain a deeper understanding of the molecular mechanisms underlying the development and metastasis of OS, this study primarily focused on the effects of POLE2 on OS, both in vitro and in vivo. We explored the tumor-promoting functions of POLE2 in OS cell lines and a nude mouse model. Importantly, we investigated the downstream regulatory mechanism of POLE2, and found that POLE2 inhibited the ubiquitination degradation of CD44 to promote tumor progression. These findings might provide potential molecular targets for the diagnosis and treatment of OS.

## Results

### POLE2 was upregulated in OS

**Fig. 1:**
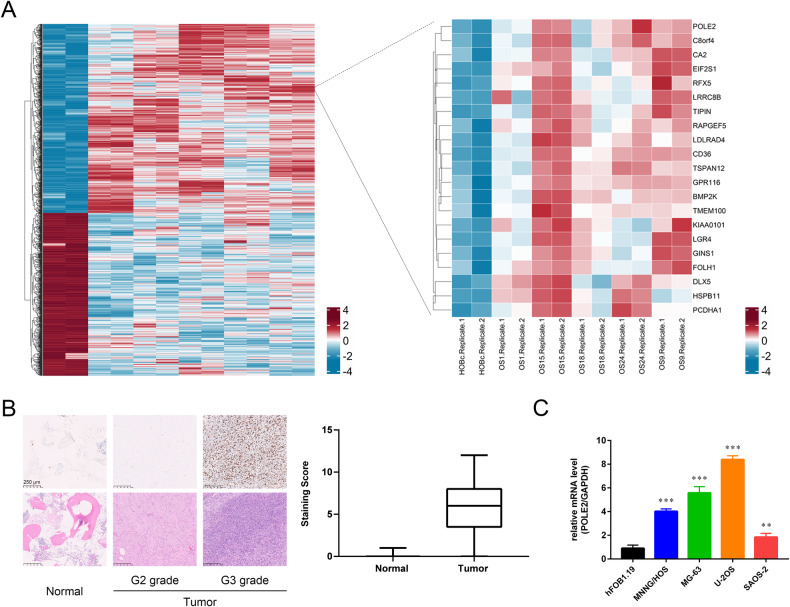
POLE2 was upregulated in the OS tissues and cells. **A** GSE14359 chip from GEO public database was used for gene differential expression analysis using R studio. The screening standards were│log2 FC│ > 1 and adjusted *P* < 0.05. **B** The expression levels of POLE2 in OS tissues and normal tissues were detected by immunohistochemical staining, and the histological morphology was observed under microscope after H&E staining. Quantification of immunohistochemical staining were shown in the box plots. The scale bar is 250 μm. **C** The expression of POLE2 in OS cell lines and hFPB1.19 cells was measured by qPCR. **P < 0.01, ***P < 0.001.

GSE14359 chip was utilized to identify differentially expressed genes (DEGs) in order to investigate dysregulated genes in OS. As depicted in Fig. 1A, POLE2 exhibited a significant upregulation among all the DEGs, with one of the highest fold changes observed in OS tissues. Moreover, the immunohistochemical staining results of OS tissue microarray demonstrated a significantly elevated expression level of POLE2 in OS tissues compared to that in normal tissues (Fig. 1B, Table 1). Similarly, the upregulation of POLE2 expression was also observed in OS cell lines (MNNG HOS, MG-63, U-2OS and SAOS-2) (Fig. 1C). Of note, a higher level of POLE2 was found in OS tissues with more advanced tumor grades (Fig. 1B). All the results indicated that POLE2 was significantly upregulated in OS tissues. To investigate the pivotal role of POLE2 in OS, MNNG HOS and U-2OS cell lines were selected for constructing the POLE2 knockdown cell models. Based on qPCR results, shPOLE2-1 (RNAi-20909) exhibited the most efficient knockdown among 3 designed shRNAs with POLE2 as a template, and was used for subsequent studies (Fig. S1A). The results of fluorescence imaging revealed that the efficiency of lentivirus infection reached 80% (Fig. S1B). In addition, both qPCR and western blot analyses demonstrated the significant downregulation of POLE2 by shPOLE2 (Figs. S1C, D and S5A), indicating that POLE2 knockdown cell models were constructed successfully.

**Table 1:** Expression patterns in osteosarcoma tissues and normal tissues revealed in immunohistochemistry analysis.

POLE2 expression	Tumor tissue		Normal tissue		*P* value
	Cases	Percentage	Cases	Percentage	
Low	28	43.8%	22	100%	<0.001***
High	36	56.2%	0	-	

### POLE2 played a pivotal role in promoting OS progression

**Fig. 2:**
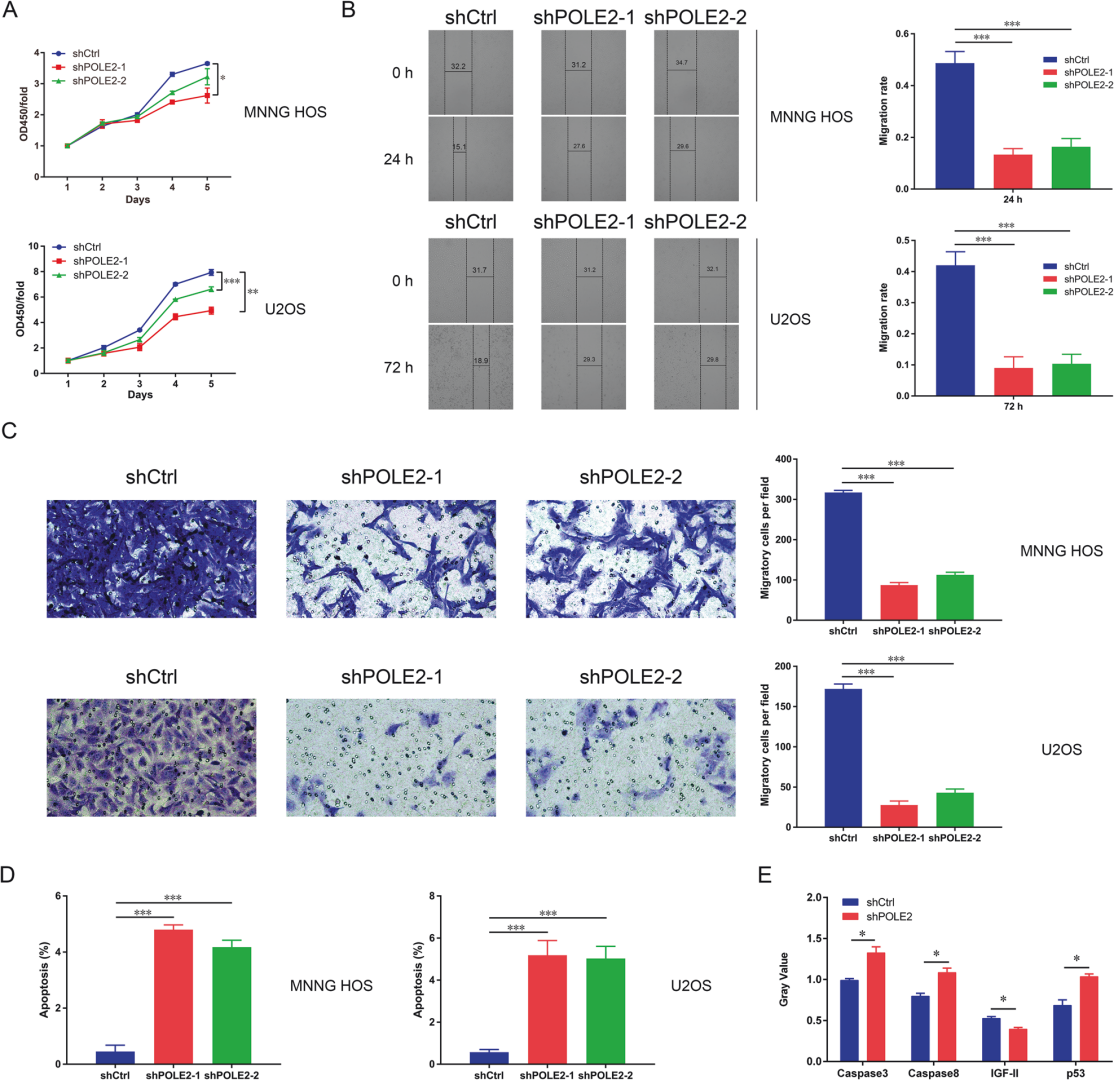
The effects of POLE2 on OS cells in vitro. **A** CCK-8 assay was used to test the proliferation of OS cells. Cell proliferation ability was evaluated by the OD value of CCK-8 assay at 450 nm. **B** Wound healing assay was performed to detect the migration ability of MNNG HOS and U-2OS cells (magnification: ×50). **C** The migratory ability of OS cells was measured by trans-well assay (magnification: ×200). The scale bar is 100 μm. **D** The apoptosis of OS cells after infection with shPOLE2s was determined by flow cytometry (FCM). **E** The Human Apoptosis Antibody Array was used to investigate the changes of cell apoptosis- related proteins in MNNG HOS cells after POLE2 knockdown. Histogram was used to show the proteins with significant changes. ShCtrl: cells infected with negative control shRNA; shPOLE2: cells infected with POLE2 shRNA. *P < 0.05, **P < 0.01.

CCK-8 assay was employed to assess cell proliferation, and the findings indicated that POLE2 knockdown markedly impeded the proliferation of OS cells (Fig. 2A). The wound healing assay demonstrated that the inhibition of POLE2 resulted in a diminishment in migration ability of OS cells (MNNG HOS and U-2OS), which was further validated by trans-well assay (Fig. 2B, C). Meanwhile, the infection of shPOLE2s led to an increase in apoptosis rate of OS cells (Fig. 2D). Moreover, the results of Human Apoptosis Antibody Array showed that shPOLE2 upregulated the expression of Caspase 3, Caspase 8 and p53 while downregulating the expression of IGF-II in OS cells (Fig. 2E and Table S4). These findings suggested that POLE2 might inhibit OS cell apoptosis by modulating these proteins.

**Fig. 3:**
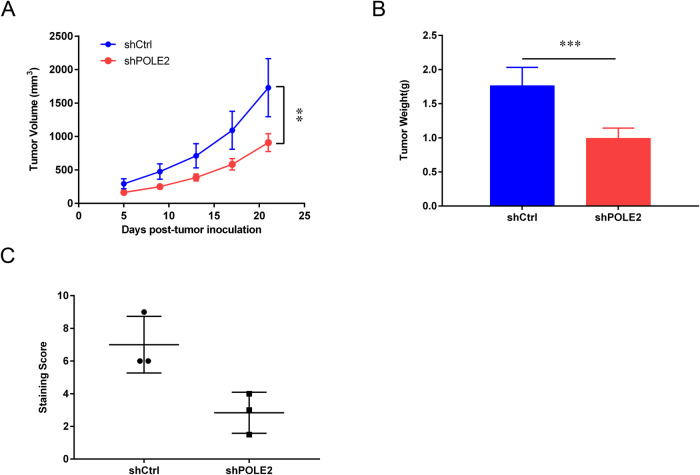
The effects of POLE2 on OS in vivo. **A** The tumor volume was detected by Vernier caliper. **B** The weight of the tumor in nude mice. **C** The expression levels of Ki-67 in the tumor tissues of shCtrl group and shPOLE2 group were evaluated by immumohistochemical staining, and the results of quantification of immunohistochemical staining were shown in box plots. ShCtrl: cells infected with negative control shRNA; shPOLE2: cells infected with POLE2 shRNA. **P < 0.01, ***P < 0.001.

Furthermore, POLE2 knockdown animal models were constructed by subcutaneous injecting shPOLE2 OS cells, to explore the role of POLE2 in vivo. The tumors in the shPOLE2 group exhibited significantly smaller volume and weight than those in the shCtrl group (Fig. 3A, B). Immunohistochemical staining results showed a distinct decrease in Ki67 expression within the shPOLE2 group (Fig. 3C, D).

To sum up, the in vitro and in vivo results collectively suggested that knockdown of POLE2 exerted inhibitory effects on OS cell proliferation and migration as well as the growth of OS tumors.

### POLE2 regulated the stability of CD44 via MDM2-mediated ubiquitination modification

**Fig. 4:**
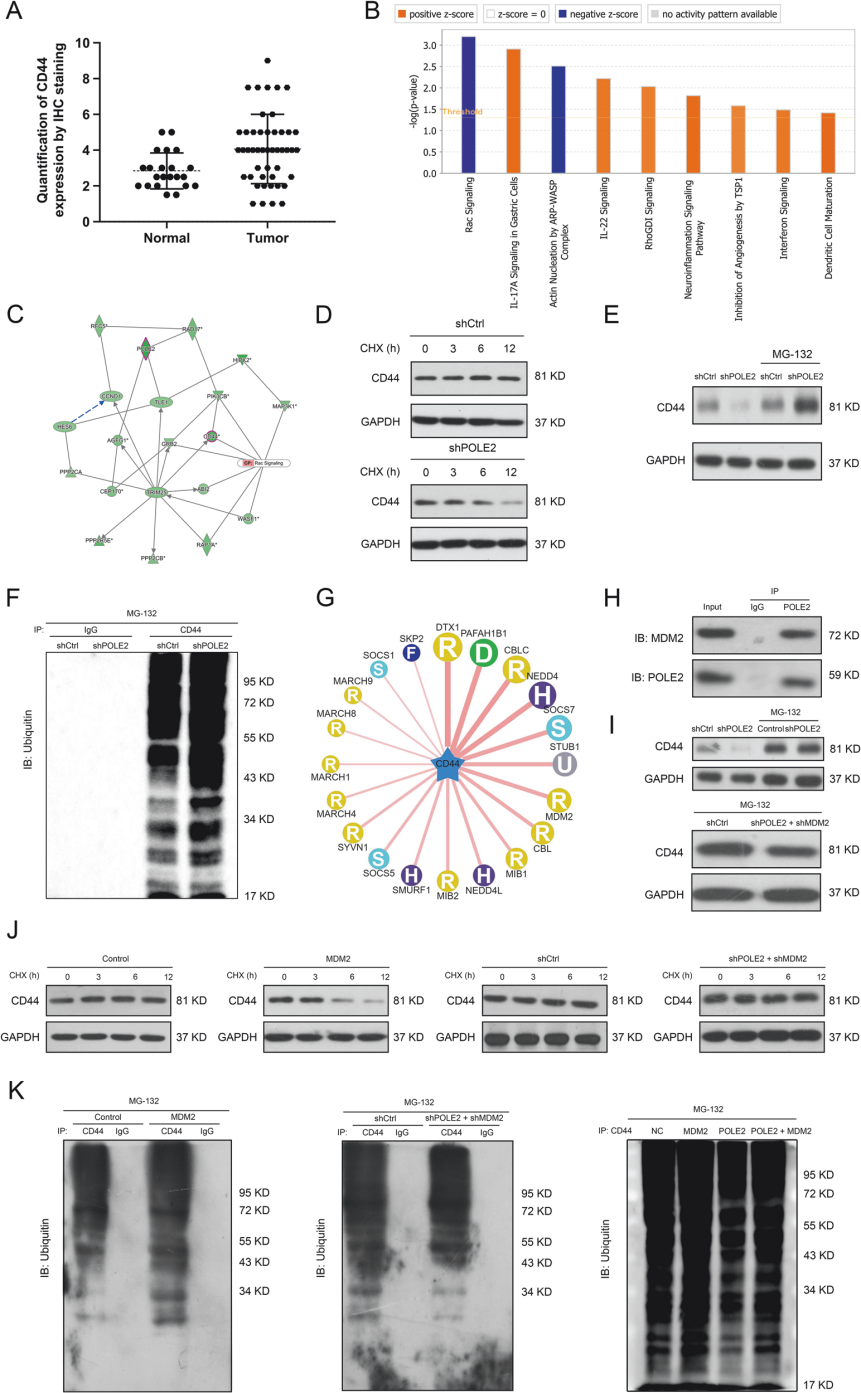
The exploration of potential regulatory mechanism of POLE2 in OS. **A** The expression of CD44, a downstream molecule of POLE2, was determined by immunohistochemical staining, and the results of quantification of immunohistochemical staining were shown in the scatter diagram. **B** The differentially expressed genes were used to perform pathway enrichment analysis. **C** Molecular network among POLE2, CD44, and Rac signaling pathway was conducted by IPA. **D** Western blot was performed to detect the levels of CD44 protein in POLE2 knockdown MNNG HOS cells after CHX treatment. CHX: 0.2 mg/mL. **E** Western blot was used to measure the levels of CD44 protein in POLE2 knockdown MNNG HOS cells with or without MG-132 treatment. MG-132: 20 μM. **F** After treating MNNG HOS cells infected or uninfected with shPOLE2 lentivirus with MG-132, immunoprecipitation (IP) was performed with CD44 and IgG to evaluate the ubiquitination levels of CD44 protein. **G** The UbiBrowser database was used to predict human ubiquitin ligase (E3) targeting CD44 protein. **H** The Co-IP assay was conducted to determine the interaction between POLE2 and MDM2. **I** Western blot was used to measure the levels of CD44 protein in MDM2 overexpression and double knockdown of POLE2 and MDM2 MNNG HOS cells with or without MG-132 treatment. MG-132: 20 μM. **J** Western blot was performed to detect the levels of CD44 protein in MNNG HOS cells that were treated with MDM2 overexpression or double knockdown of POLE2 and MDM2 after CHX administration. CHX: 0.2 mg/mL. **K** After individual or synergistic interference with the expression of MDM2 and POLE2 expression in MNNG HOS cells with MG-132, IP was performed to evaluate the ubiquitination levels of CD44 protein. ShCtrl: cells infected with negative control shRNA; shPOLE2: cells infected with POLE2 shRNA; Control: cells infected negative control lentivirus; MDM2: cells infected with MDM2 overexpression lentivirus; POLE2: cells infected with POLE2 overexpression lentivirus; shPOLE2+shMDM2: cells with infected with POLE2 shRNA and MDM2 shRNA; POLE2 + MDM2: cells with infected with POLE2 and MDM2 overexpression lentivirus.

In order to further investigate the downstream regulatory mechanism of POLE2 in OS, differentially expressed genes (DEGs) in MNNG HOS cells infected with shPOLE2 lentivirus were screened using Limma package in R studio (Table S5). ITGA2, CD44, RAP1A, PIK3CB and ARNT were found to be markedly inhibited by shPOLE2 (Fig. S2A). After perturbing the expression of the above five genes in OS cells separately, MTT assay results displayed that downregulation of CD44 or RAP1A significantly inhibited the proliferation of MNNG HOS cells (Fig. S2B). Based on the results of differential analysis, qPCR and MTT assays, CD44 was chosen as the downstream molecule of POLE2 for subsequent exploration. qPCR and western blot results indicated that the mRNA and protein levels of CD44 were considerably downregulated in MNNG HOS cells after shPOLE2 lentivirus infection (Figs. S2C and S5B). CD44 expression was also found to be upregulated in OS tissues compared to normal tissues (Fig. 4A). Furthermore, pathway enrichment analysis based on all the DEGs showed that Rac signaling pathway was significantly inhibited in the shPOLE2 group compared to the control group (Fig. 4B). Molecular network analysis indicated a connection between POLE2, CD44 and Rac signaling pathway (Fig. 4C). More importantly, CD44 was found to be an upstream gene in the Rac signaling pathway (Fig. S2D). Western blot results showed that downregulation of POLE2 restrained the expression and phosphorylation of Rac signaling pathway related proteins, suggesting that POLE2 knockdown suppressed the activation of Rac signaling pathway (Figs. S2E and S5C). Therefore, the Rac signaling pathway was the downstream pathway of POLE2.

Protein stability is largely regulated by post-translational modifications. Protein ubiquitination is one of the most important post-translational modifications, and is activated by ubiquitin-activating enzyme E1, ubiquitin-conjugating enzyme E2, and substrate-specific ubiquitin ligase E3 [18, 19]. When treated with the protein synthesis inhibitor CHX, CD44 protein levels in MNNG HOS cells were significantly reduced in a time-dependent manner, and the reduction of CD44 in the POLE2 knockdown group was markedly higher than that in the control group (Fig. 4D and Fig. S6A). Besides, the downregulation of CD44 protein levels by POLE2 deficiency was partially mitigated by the proteasome inhibitor MG-132 (Fig. 4E and Fig. S6B). More importantly, POLE2 knockdown enhanced the ubiquitination of CD44 protein (Fig. 4F and Fig. S6C), suggesting that POLE2 might affect the stability of CD44 protein by modulating ubiquitination modification. A previous study has reported that CD44 interacted with E3 ligase and is one of the targets affected by E3 ligase-dependent lysosomal degradation [20]. E3 ligases targeting CD44 were predicted using the UbiBrower database, and the interactions between POLE2 and these E3 ligases were analyzed by STRING (Fig. 4G and Fig. S3A). Among them, MDM2 had a strong relationship with POLE2 and CD44 (Fig. S3A). Vlatkovic et al. showed that MDM2 interacted with a region at the C-terminal of DNA polymerase epsilon [21]. Moreover, the findings of Co-IP confirmed the interaction between POLE2 and MDM2 (Fig. 4H and Fig. S6D). Levels of CD44 protein in MNNG HOS cells overexpressing MDM2 were obviously downregulated, which was alleviated by MG-132 (Fig. 4I and Fig. S6E). Of note, upregulation of MDM2 not only destabilized CD44 protein, but also boosted the levels of CD44 ubiquitination (Fig. 4J, K and Fig. S6F, G). In summary, POLE2 knockdown increased the levels of CD44 ubiquitination that relies on MDM2, thereby destroying the stability of CD44 protein and reducing CD44 protein in OS cells.

### POLE2 promoted OS development via regulating CD44

To verify whether POLE2 affected OS progression via regulating CD44 expression, we constructed a POLE2 overexpression cell model, CD44 knockdown cell model, and POLE2 overexpression while CD44 knockdown cell model in vitro. As expected, CD44 was highly expressed in OS cell lines (MNNG HOS, MG-63, U2OS and SAOS-2) compared with hFOB 1.19 cells (Fig. S4A). qPCR and western blot were used to detect the overexpression or knockdown efficiency. As shown in Fig. S4B, C and Fig. S7A, cell models in vitro were constructed successfully.

**Fig. 5:**
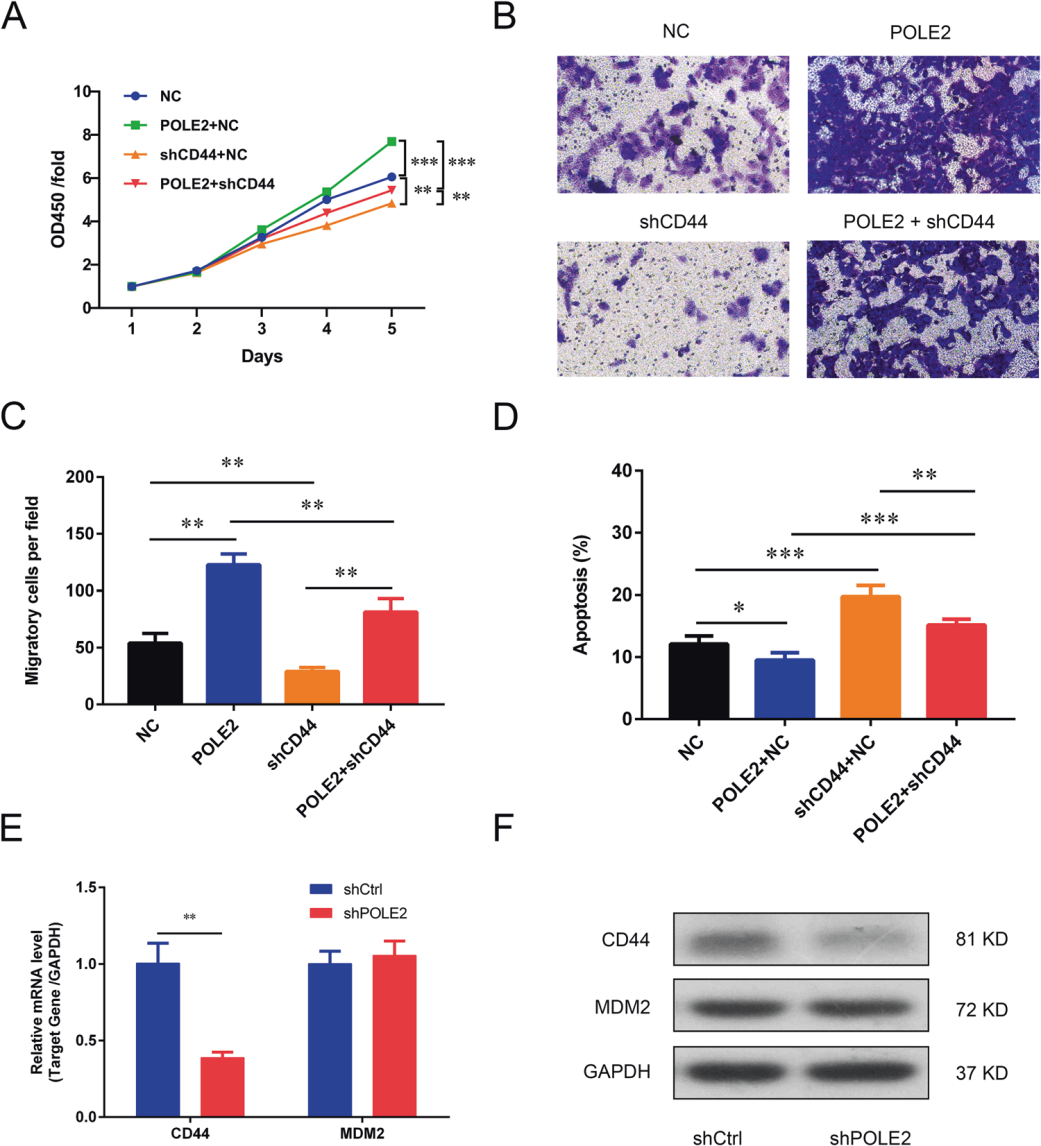
POLE2 affected the biological behevior of MNNG HOS cells via regulating CD44 expression. **A** The MTT assay was used for assessing the proliferation ability of MNNG HOS cells after overexpressing POLE2, knockdown CD44, or overexpressing POLE2 while knockdown CD44. **B**, **C** The invasive ability of MNNG HOS cells was determined by trans-well assay. **D** The apoptosis of MNNG HOS cells in POLE2 overexpression group, shCD44 group, and POLE2 + shCD44 group was detected by FCM. **E**, **F** The mRNA and protein expression of CD44 and MDM2 in tumor tissues of POLE2 knockdown nude mice models were detected by qPCR and western blot, respectively. POLE2: cells infected with POLE2 overexpression lentivirus; shCtrl: cells infected with negative control shRNA; shCD44, cells infected with CD44 shRNA; POLE2 + shCD44, cells co-infected with POLE2 overexpression vector and CD44 shRNA. *P < 0.05, **P < 0.01, ***P < 0.001.

Overexpression of POLE2 promoted MNNG HOS cell proliferation, knockdown of CD44 suppressed cell proliferation, while downregulation of CD44 could counteract the promoting effect of POLE2 upregulation on MNNG HOS cell proliferation (Fig. 5A and Fig. S4D). Trans-well assay results revealed that POLE2 overexpression promoted migration of MNNG HOS cells, and CD44 downregulation inhibited cell migration (Fig. 5B, C and Fig. S4E, F). In addition, Flow cytometry results showed that POLE2 overexpression decreased the apoptosis of MNNG HOS cells, while CD44 downregulation increased the apoptosis rate of OS cells. CD44 downregulation alleviated the inhibitory effect of POLE2 overexpression on cell apoptosis (Fig. 5D and Fig. S4G). Furthermore, knockdown of POLE2 also inhibited the expression levels of CD44 in vivo (Fig. 5E, F and Fig. S7B).

## Discussion

OS is a common primary malignant bone tumor and ranks sixth among children under the age of fifteen [22, 23]. There is an urgent need to explore the molecular mechanisms of OS development to develop more effective treatments. This study utilized GSE14359 chip data from the GEO public database for differential expression analysis. It was found that POLE2 was upregulated in OS, which was consistent with our subsequent experimental results, demonstrating significantly higher levels of POLE2 expression in OS cell lines and tissues compared to normal tissues. Li et al. demonstrated the oncogenic effect of POLE2 on lung adenocarcinoma [24]. Moreover, Zhang et al. reported that high expression of POLE2 was associated with a poor prognosis of renal cell carcinoma (RCC), with knockdown of POLE2 dramatically inhibiting the proliferation and migration of RCC cells [25]. Recently, an in vivo study indicated a positively correlation between POLE2 and tumor formation in esophageal squamous cell carcinoma [26]. In this study, the in vitro findings illustrated that POLE2 knockdown inhibited the proliferation and migration of OS cells while facilitating cell apoptosis. More importantly, the tumor growth was dramatically suppressed in the shPOLE2 group when compared to the shCtrl group. In brief, POLE2 played an essential role in promoting the development of OS.

Considering that the specific mechanisms of POLE2 in OS progression was still unclear, DEGs in MNNG HOS cells with POLE2 knockdown were screened, and the downstream targets and pathways of POLE2 were identified using bioinformatics analysis. The expression of CD44 and Rac signaling pathway-related protein were inhibited in the POLE2 knockdown group, and CD44 was an upstream regulator of Rac signaling pathway. Moreover, downregulation of POLE2 significantly decreased the phosphorylation levels of Rac signaling pathway-related proteins (PI3K and RAC). Geoueffic Y et al. reported that CD44 activated Rac pathway and was involved in cell migration [27]. Therefore, POLE2 activated Rac signaling pathway by upregulating CD44 expression, and then participated in the progression of OS.

CD44 (cluster of differentiation 44) is a surface glycoprotein encoded by the CD44 gene on chromosome 11q13 [28], and it is involved in cell-cell interactions, cell adhesion and migration [29]. Our findings indicated that the level of CD44 protein in MNNG HOS cells was decreased significantly after POLE2 knockdown. The ubiquitin-proteasome system (UPS) regulates multiple signaling pathway and plays a key role in controlling protein homeostasis and developmental decisions [30]. It has been reported that upstream genes can regulate the level of CD44 protein in melanoma through promoting the ubiquitination and degradation of CD44 [31]. Therefore, we speculated that POLE2 might affect CD44 protein levels by regulating CD44 ubiquitination. As illustrated in Fig. 4F, POLE2 knockdown diminished the expression of CD44 by destabilizing CD44 protein. The downregulation of CD44 by POLE2 knockdown was alleviated when the proteasome was inhibited using MG-132. Besides, CD44 protein ubiquitination was upregulated after POLE2 depletion. Consequently, POLE2 manipulated CD44 expression through the ubiquitin-proteasome pathway. It has been reported that CD44 was a target of E3 ubiquitin ligases [20]. In this study, the relationship between POLE3 and E3 ligases targeting CD44 was further explore. MDM2, as an E3 ligase that acted on CD44, was manifested to interact with POLE2. MDM2 induced the degradation of the target proteins by ubiquitination [32, 33]. Our results found that overexpression of MDM2 destroyed the stability of CD44 protein and increased the ubiquitination level of CD44, suggesting that POLE2 inhibited CD44 protein ubiquitination by interacting with MDM2 to enhance the stability of CD44.

CD44 was highly expressed in OS cells and was related to the metastatic potentiality [28, 34]. In addition, CD44 upregulation promoted tumor cells proliferation, tumor stem cell differentiation and drug resistance [35]. This study manifested that CD44 was upregulated in OS tissues and cell lines. More importantly, the proliferation and migration of MNNG HOS and U-2OS cells were promoted by POLE2 overexpression but suppressed by CD44 knockdown, which was consistent with the previous reports [25, 35]. The tumor-promoting effects of POLE2 were alleviated by downregulating CD44 expression. Additionally, CD44 knockdown induced OS cell apoptosis, which was attenuated through POLE2 overexpression. Based on previous studies and our results, we concluded that POLE2 promoted tumor progression of OS cells by maintaining the expression of CD44.

In conclusion, the role of POLE2 in OS was investigated in vitro and in vivo, and these results demonstrated that POLE2 might be a potential target for the treatment of OS. Moreover, POLE2 enhanced the stability of CD44 through inhibiting MDM2-mediated ubiquitination. However, the role of POLE2 and CD44 in OS progression should be further confirmed in large clinical sample size.

## Materials and methods

### Microarrays analysis from GEO database

To investigate molecular mechanisms of OS development and metastasis, the GSE14359 chip from Gene Expression Omnibus (GEO) database was used for microarrays analysis. Two non-neoplastic primary osteoblast cells were used as the control group, and 10 conventional osteosarcoma tissues were used as the tumor group. The data was then analyzed by Limma package of R studio and the screening criteria for differentially expressed genes were |log2 fold change| > 1 with the adjusted *P* value < 0.05.

### Tissues chip

The OS tissue microarrays (OS804c and BO244f) used in this study were purchased from Xi’an Alena biotechnology Co. LTD (Xi’an, China) to determine POLE2 and CD44 expression in OS tissues and normal tissues. There were 86 samples in these tissue microarrays, including 64 OS tissues and 22 normal tissues. All human tissues were obtained with the informed consent of the patients and their families. All experiments involved in this study were approved by the Ethics committee of Huazhong University of Science and Technology.

### Cell culture

The OS cell lines (MNNG HOS, MG-63, U-2OS, and SAOS-2), and hFOB1.19 cell line (human osteoblasts transfected with SV40) were purchased from BeNa Culture Collection (Beijing, China). MNNG HOS cells were cultured in MEM with 10% fetal bovine serum (FBS). MG-63 and hFOB1.19 cells were cultured in DMEM-H with 10% FBS. U-2OS cells were cultured in the RPMI-1640 with 10% FBS. SAOS-2 cells were cultured in the McCOY’s 5 A with 15% FBS. All the cells were cultured under an atmosphere of 5% CO_2_ at 37 °C.

### Immunohistochemical staining

After the paraffin sections of OS tissue microarrays were dewaxed, antigen repair was performed on the paraffin sections with citrate buffer (Maxim Biotechnology Development Co. LTD, Fuzhou, China). After washing with 1 × PBS, the sections were blocked by using 3% H_2_O_2_ for 5 min and then blocked for 15 min with 5% serum. After that, the primary antibody was used to incubate the sections at 4 °C overnight. The second antibody was then used to incubate the sections at 37 °C for 1 h. Afterwards, the sections were stained by DAB in the dark for 5 min, and re-dyed with hematoxylin (Baso Biotechnology Co. LTD, Zhuhai, China) for 15 s. The sections were finally sealed with neutral gum and photographed using light microscopy. The primary and the second antibodies used in this assay were shown in Table S1.

### Hematoxylin-eosin staining (H&E staining)

The sections were dewaxed and hydrated with xylene, absolute ethanol, alcohol and distilled water. Then the sections were stained with hematoxylin for 5 min. After washing with water, sections were differentiated with 1% hydrochloric acid alcohol for several seconds, then returned to blue with 0.6% ammonia water, and rinsed again under running water. After that, the sections were stained in eosin staining solution for 2 min. Finally, the sections were put into 95% alcohol, absolute ethanol and xylene successively in order to dehydrate and make them transparent. The sections were taken out from xylene and dried, then sealed with neutral gum, observed and photographed under the microscope.

### Lentivirus transfection and knockdown gene cells model

RNAis of POLE2 and CD44 were designed and shown in Table S2. The target sequences were synthesized into DNA oligo, and the double chains of DNA oligo formed. Then, they were connected to the vectors under the action of Fermentas T4 DNA Ligase and transfected into the cells. Cells were harvested, and the lentiviruses were purified and concentrated. Finally, the lentivirus solution was infected with OS cells with a virus titer of 1 × 10^8^ TU/mL. The expression of green fluorescent protein (GFP) carried by lentivirus was used to evaluate the infection efficiency.

### qPCR

After cell collection, total RNA was extracted with Trizol buffer (Sigma-Aldrich, St. Louis, Missouri, USA). Then, the extracted RNA was converted to cDNA by reverse transcription using Hiscript QRT supermix for qPCR (+ gDNA WIPER) (Vazyme Biotech Co., Ltd, Nanjing, China) under the manufacturer’s instructions. Real-time PCR was used to detect the mRNA expression. The formula 2^-∆∆Ct^ was used to calculate the relative mRNA expression. Primer sequences were shown in Table S3.

### Western blot (WB)

Cells were lysed by RIPA lysis buffer, and the total proteins were extracted, the concentration of which was tested by using BCA Protein Assay Kit (HyClone-Pierce, Logan, UT, USA). 20 µg of proteins per lane were separated by 10% SDS-PAGE gel, and then transferred to poly vinylidene fluoride (PVDF) membranes. The membranes were blocked by TBST solution with 5% non-fat milk at room temperature for 1 h. Primary antibodies were then added for incubation overnight at 4 °C. Afterwards, the membranes were incubated with HRP-conjugated secondary antibodies at room temperature for 2 h. Finally, the membranes were visualized using the ECL-Plus™ western blot system (GE Healthcare Life Sciences, USA), and densitometric analysis was performed using Image J. The primary antibodies and the second antibodies involved in this assay were shown in Table S1.

### CCK-8 assay

MNNG HOS and U-2OS cells infected with negative control shRNA or POLE2/CD44 shRNAs were seeded into 96-well plates with 2000 cells per well. After 24 h, cell proliferation was assessed by CCK-8 assay for 5 consecutive days. 20 μL of CCK-8 (Sigma-Aldrich) was added 96-well plates for 4 h. After oscillating for 2–5 min, a microplate reader (Tecan infinite, Männedorf, Switzerland) was used to detect the OD value at a wavelength of 450 nm. Three independent repetitions were performed in this assay.

### MTT assay

MNNG HOS cells infected with lentivirus were seeded into 96-well plates with 3000 cells per well. 20 μL of 5 mg/mL MTT (Genview, Florida, USA) was added to each well for 4 h before the end of culturing, from the second day after the planking. After 4 h, 100 μL of DMSO solution was added. The OD value was then detected at the wavelength of 490 nm using a microplate analyzer (Tecan infinite, Mannedorf Zurich, Switzerland). Three independent experiments were set up in this assay.

### Flow cytometry (FCM)

The experimental cells were grown in 6-well plates, and when the cell density reached 85%, the cells were digested and centrifuged. The cell deposits were washed using D-Hanks precooled at 4 °C. 10 μL of Annexin V-APC (eBioscience, California, USA) was added to stain cells in the dark for 15 min at room temperature and the flow cytometry (Millipore, Schwalbach, Germany) was finally used to detect and analyze cell apoptosis. Three independent repetitions were performed in this assay.

### Wound healing assay

4 × 10^4^ MNNG HOS or U-2OS cells were seeded into 96-well plates. A scratch was made from the bottom in the middle of the 96-well plates when the convergence degree of cells reached about 90%. Cells were cultured at 37 °C with 5% CO_2_ and 96-well plates were scanned using Cellomics (Thermo, Massachusetts, USA) after scratch formation at 0 h, 24 h and 72 h, and the cell migratory area was evaluated. After that, the cell migration rate was then calculated according to the migratory area. The experiment was independently repeated for 3 times.

### Trans-well assay

Trans-well chambers (24-well, 8-mm pore) were purchased from Corning Company (NY, USA). The chambers were rehydrated with 100 μL serum-free medium for 1–2 h at 37 °C. Cells in the logarithmic growth period were harvested and resuspended in a serum-free medium. 100 μL of the cell suspension (total 1 × 10^5^ cells) was added into the upper chamber, and 500 μL culture medium containing 30% FBS was added into the lower chamber to incubate for 16 h at 37 °C. Non-invading cells on the upper chamber were then removed with a cotton swab. The cells adhering to the membrane were fixed in 4% paraformaldehyde for 30 min and stained with 0.1% crystal violet for 20 min at room temperature. 5 fields of view per well were selected randomly under a 200 × microscope, and images were captured. Three independent experiments were set up in this experiment.

### Human Apoptosis Antibody Array chip assay

Human Apoptosis Antibody Array Kit (Abcam, Cambridge, MA, USA) was analyzed to investigate the expression changes of proteins related to cell apoptosis. MNNG HOS cells after lentivirus infection were collected and lysed with 1 × Lysis buffer for 30 min at 4 °C. Then the total protein was extracted and diluted to 0.5 mg/mL in Array Diluent Buffer provided by the kit. The array antibody membrane was blocked by 1 × Blocking Buffer for 30 min at room temperature and then incubated with 1 × Biotin-conjugated Anti-Cytokines overnight at 4 °C. HRP linked Streptavidin was added to the membranes. Finally, proteins were visualized using ChemiDoc XRS chemiluminescence detection (Bio-Rad Laboratories, Hercules, California, USA) and imaging system. The density of the spots was quantitated using Quantity One software and normalized to the α-tubulin levels. In this experiment, a double-hole repetition was set up.

### Tumor formation model in nude mice

4-week female nude mice were purchased from Shanghai SLAC Laboratory Animal CO. LTD (Shanghai, China), and randomly divided into two groups, 8 mice per group. The 4 × 10^6^ MNNG HOS cells infected with shCtrl or shPOLE2 were subcutaneously injected into the nude mice. These mice were then fed for 49 days, during which the volume of tumors in the mice was measured by Vernier caliper. Finally, mice were sacrificed by over-dose of 0.7% pentobarbital sodium and tumors were collected from the mice. After measuring the weight and volume, the tumors were stored in liquid nitrogen at −80 °C. The expression of Ki67 in tumor tissues from mice was detected by immunohistochemical staining, and CD44 levels as well as MDM2 levels were determined by qPCR and western blot. Information on relevant primary and secondary antibodies was provided in Table S1. In this experiment, we used a double blind method. Animal experiments were approved by Institutional Animal Care and Use Committee of Huazhong University of Science and Technology.

### RNA sequencing and ingenuity pathway analysis (IPA)

Total RNA in OS cells MNNG HOS transfected with shRNA or shPOLE2 was extracted by Trizol, and then sequenced by human GeneChip primeview (Affymetrix, Santa Clara, CA, USA) according to manufacturer’s instructions. The differentially expressed genes in OS cells after POLE2 knockdown were screened based on the standards (FDR < 0.05 and |Fold change| ≥ 1.3). The IPA was then performed for all significantly differentially expressed genes. |Z-score| > 2 was considered significant. If Z-score > 0, it indicated that the pathway was activated and labeled orange, while negative Z-score indicated that the pathway was suppressed and labeled blue.

### Protein stability assay

The protein synthesis inhibitor cycloheximide (CHX, 0.2 mg/mL, S7418, Selleck) was used to treat POLE2 knockdown or MDM2 overexpression MNNG HOS cells to inhibit protein translation. Then MNNG HOS cells were lysed at 0, 3, 6, and 12 h and total protein was extracted. 20 μg total protein was utilized for western blot to detect the levels of CD44 protein, and then to evaluate the degradation rate of CD44 protein. Information on relevant primary and secondary antibodies was provided in Table S1.

### Ubiquitination assay

Ubiquitin-proteasome pathway inhibitor MG-132 (20 μM, HY-13259, MEC) was used to treat POLE2 knockdown or MDM2 overexpression MNNG HOS cells for 6 h. MNNG HOS cells were collected and lysed to obtain total protein. 20 μg total protein was subjected to western blot to detect the levels of CD44 protein. Another 1.0 mg total protein was incubated with the antibody at 4 °C overnight, and then incubated with 20 μL beads at 4 °C for 2 h. The protein-antibody-beads complex was washed twice with IP lysis buffer, and then subjected to western blot to determine the ubiquitination levels of CD44 protein using ubiquitin antibody. Information on relevant primary and secondary antibodies were provided in Table S1.

### Co-Immunoprecipitation (Co-IP)

MNNG HOS cells were harvested and lysed with precooled IP lysis buffer for 5 min on ice. BCA Protein Assay Kit was used to measure the protein concentration. 1.0 mg protein was incubated with the antibody at 4 °C overnight and then incubated with 20 μL beads at 4 °C for 2 h. The protein-antibody-beads complex was subjected to SDS-PAGE after washing twice with IP lysis buffer, and transferred to PVDF membranes. The membranes were sealed with TBST containing 5% skimmed milk at room temperature for 1 h. The membranes were incubated with primary antibodies at 4 °C overnight, and then incubated with secondary antibodies at room temperature for 2 h. After washing 3 times with 1 × TBST, chemiluminescence method was used for color development, and chemiluminescence imaging system (GE) was used for protein banding imaging. Information on relevant primary and secondary antibodies were provided in Table S1.

### Celigo cell counts assay

MNNG HOS cells with overexpressed or knocked down genes were constructed and placed into 96-well plates with 3000 cells per well. Starting from the second day after the paving, Celigo (Nexcelom Bioscience, Lawrence, Massachusetts, USA) was applied to detect the same position of the plates once a day, continuously for 5 days. Then the image software was used for cell counting and analysis. The experiment was repeated three times.

### Statistical analysis

The data involved in the study were analyzed by SPSS 23 (IBM, SPSS, Chicago, IL, USA) and GraphPad software (Graphpad Software, La Jolla, CA). Rank Sum test was used to analyze the quantitative immunohistochemical results of POLE2 in OS tissues and normal tissues. Unpaired student t-test was used to compare the difference between two groups. One-way analysis of variance (ANOVA) was performed to compare three or more means. The statistical difference was significant when P value was less than 0.05.

### Supplementary information

Supplementary figures

Supplemental Table 1

Supplemental Table 2

Supplemental Table 3

Supplemental Table 4

Supplemental Table 5

aj-checklist

Original Data File

## Data Availability

All data used in this article is showed in the article and supplementary materials.
