# Supplementary material for: POLE2 promotes osteosarcoma progression by enhancing the stability of CD44
Source: Cell Death Discov. 2024 Apr 16;10:177. doi: 10.1038/s41420-024-01875-x (PMC11021398; doi:10.1038/s41420-024-01875-x)
Supplement: Supplementary file 1 — Supplementary figures [file 41420_2024_1875_MOESM1_ESM.docx]

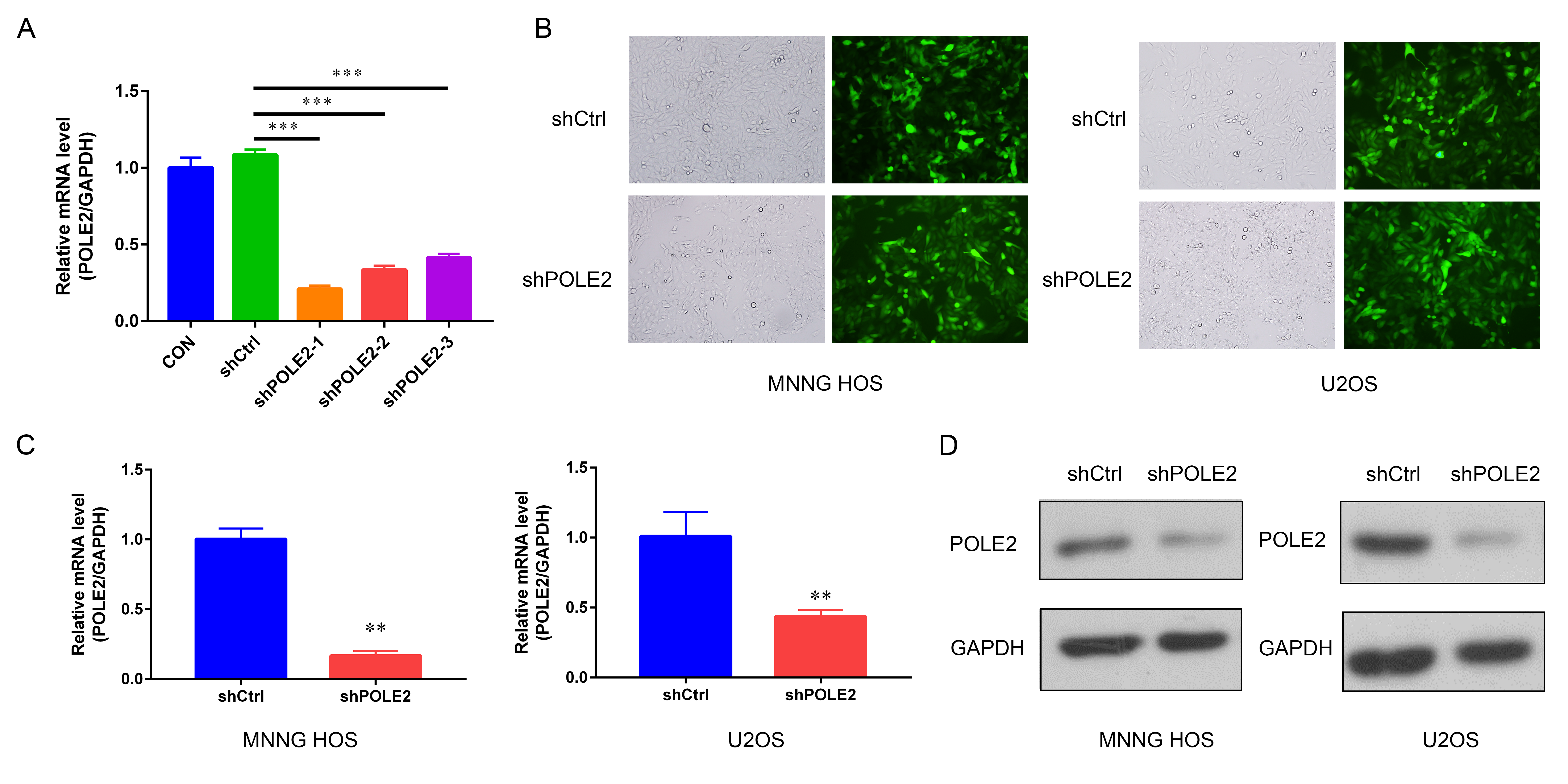


**Supplementary figure 1**

**The construction of shPOLE2 cell model**

(A) Three shPOLE2s were designed to knockdown the expression of POLE2 in MNNG HOS cells, and the knockdown efficiency of POLE2 was evaluated by qPCR. (B) The infection efficiency was assessed by observing lentivirus-mediated green fluorescent protein expression (magnification: 200 ×). (C-D) The mRNA and protein levels of POLE2 were determined by qPCR and western blot in order to evaluate the knockdown efficiency of shPOLE2. ShCtrl: cells infected with negative control shRNA; shPOLE2: cells infected with POLE2. ** P < 0.01, *** P < 0.001.


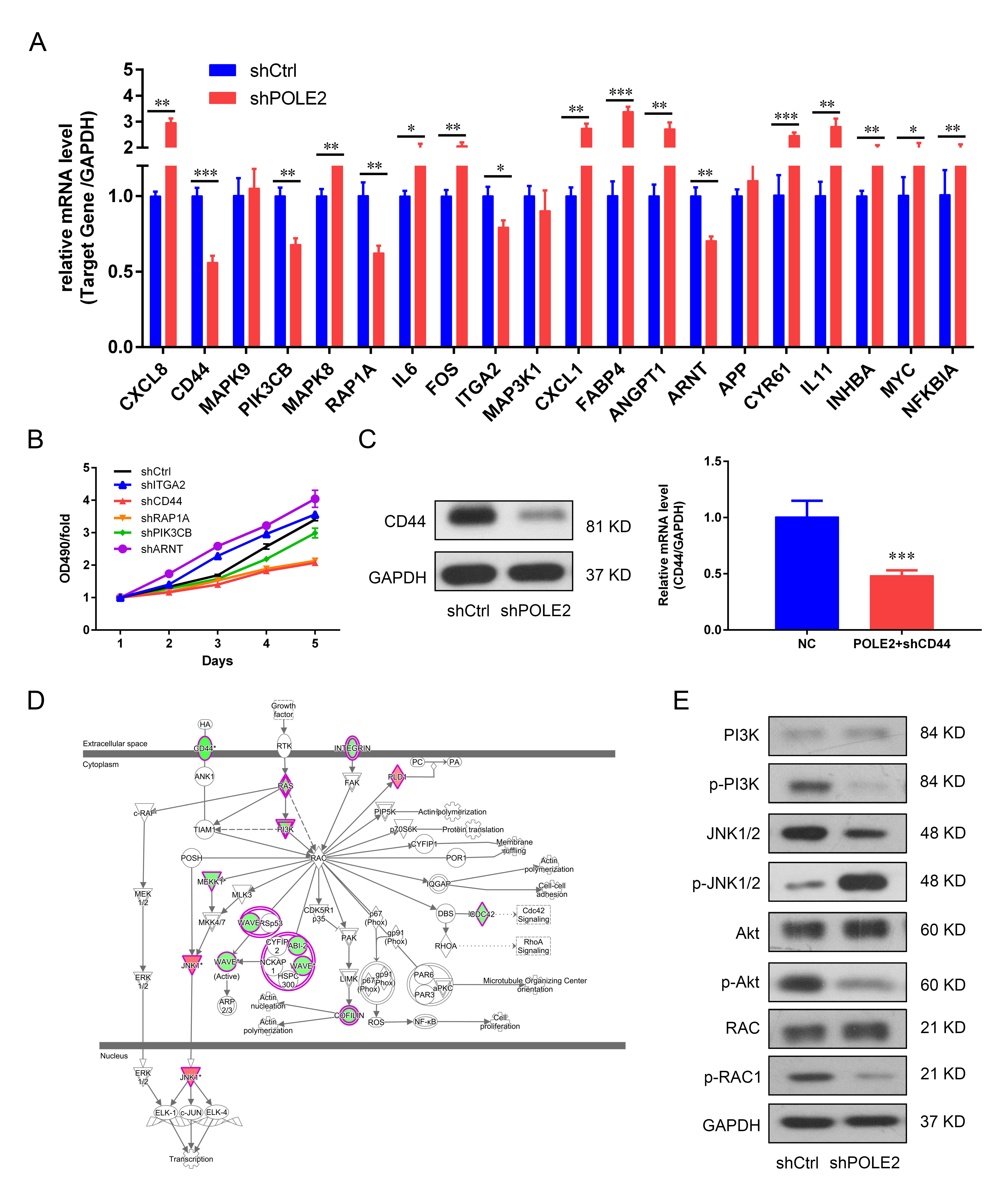


**Supplementary figure 2**

**The downstream key genes and singnaling pathway of POLE2**

(A) 20 downstream key genes of POLE2 were screened by IPA, and their expression levels were detected by using qPCR. (B) Among these 20 genes, 5 genes that were markedly downregulated after POLE2 knockdown were selected to construct gene knockdown OS cell models. MTT assay was performed to detect the cell proliferation. (C) After POLE2 knockdown, the mRNA and protein levels of CD44 were assessed by qPCR and western blot. (D) The distribution and expression of differentially expressed genes in the Rac signaling pathway. (E) The downstream pathway Rac signaling pathway was screened by IPA. Western blot was used to measure the expression of Rac signaling pathway-related proteins. ShCtrl: cells infected with negative control shRNA; shPOLE2: cells infected with POLE2 shRNA. * P < 0.05, ** P < 0.01, *** P < 0.001.


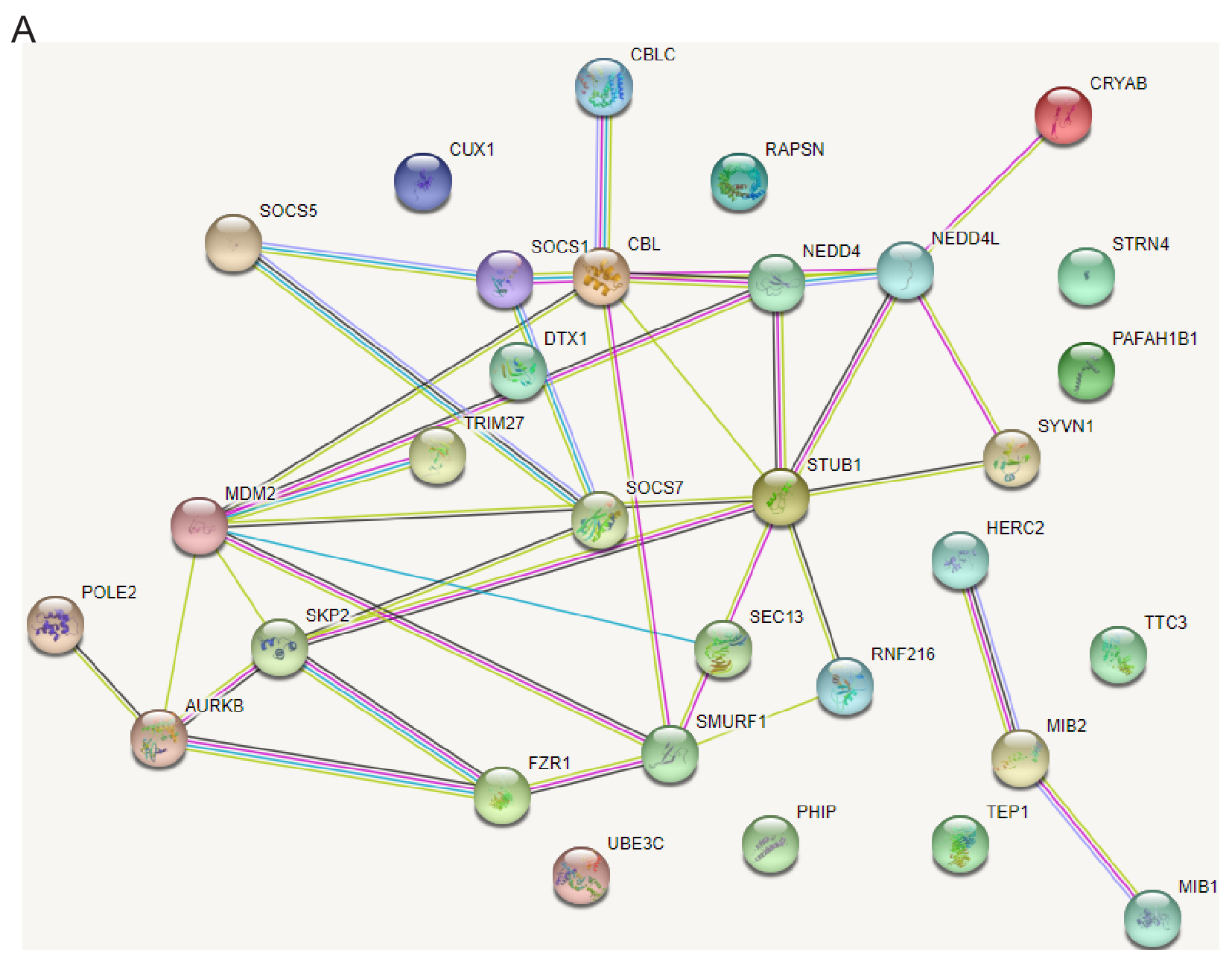


**Supplementary figure 3**

The STRING was used to predict the protein-protein interaction network between POLE2 and E3 ligase targeting CD44.


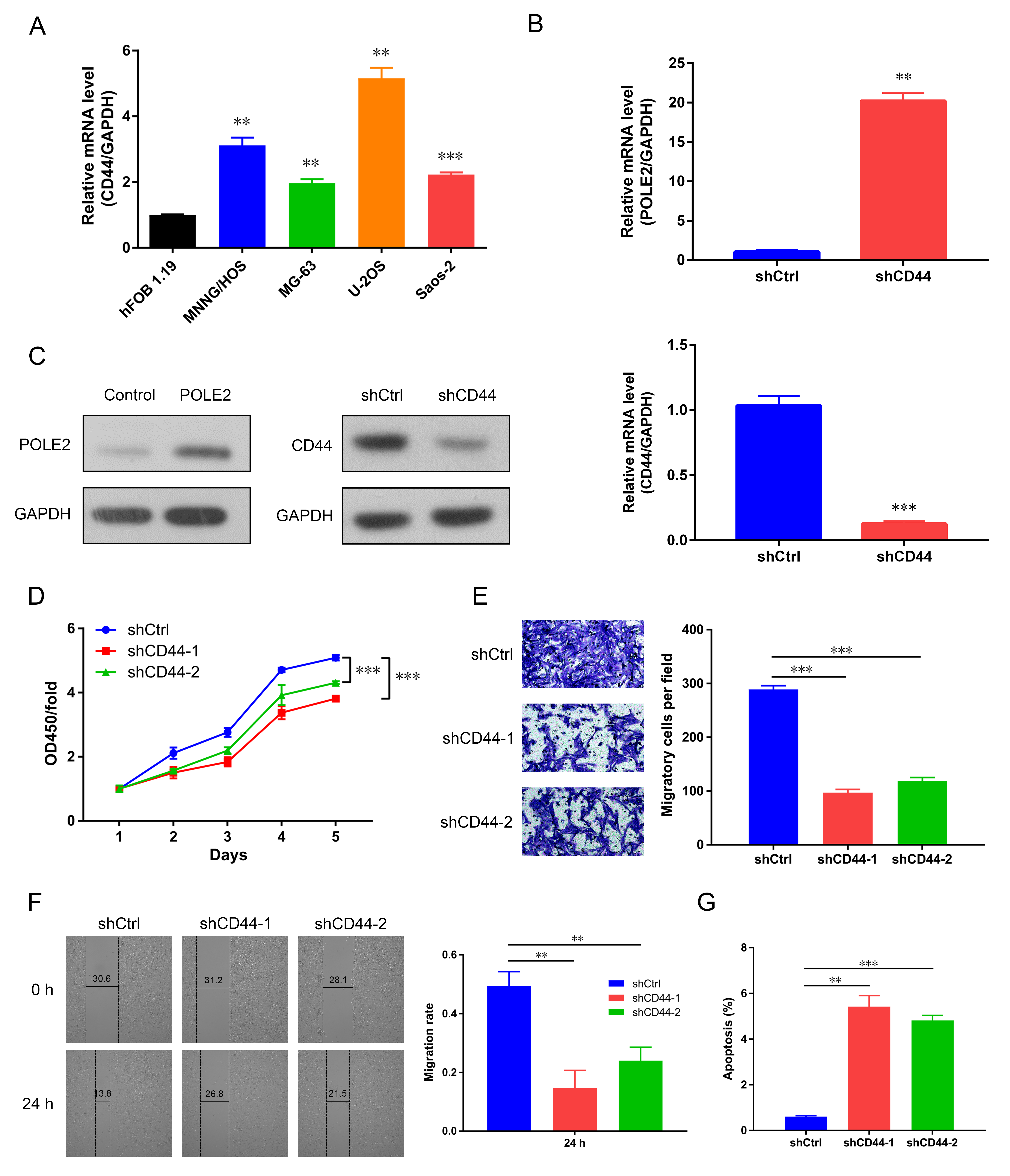


**Supplementary figure 4**

**The construction of POLE2 overexpression and CD44 knockdown cell models**

(A) The expression of CD44 in OS cell lines and hFPB1.19 cells was measured by qPCR. (B-C) The mRNA and protein levels of POLE2 and CD44 were determined by qPCR and Western blot in order to evaluate the overexpression efficiency of POLE2 and the knockdown efficiency of shCD44. (D-E) To exclude off-target effects of shCD44s, CCK-8 and Trans- well, wound healing and FCM assays were performed to detect the proliferation, migration and apoptosis of MNNG HOS cells infected with two other shCD44 lentiviruses. POLE2: cells infected with POLE2 overexpression lentivirus; shCtrl: cells infected with negative control shRNA; shCD44, cells infected with CD44 shRNA. * P < 0.05, ** P < 0.01, *** P < 0.001.


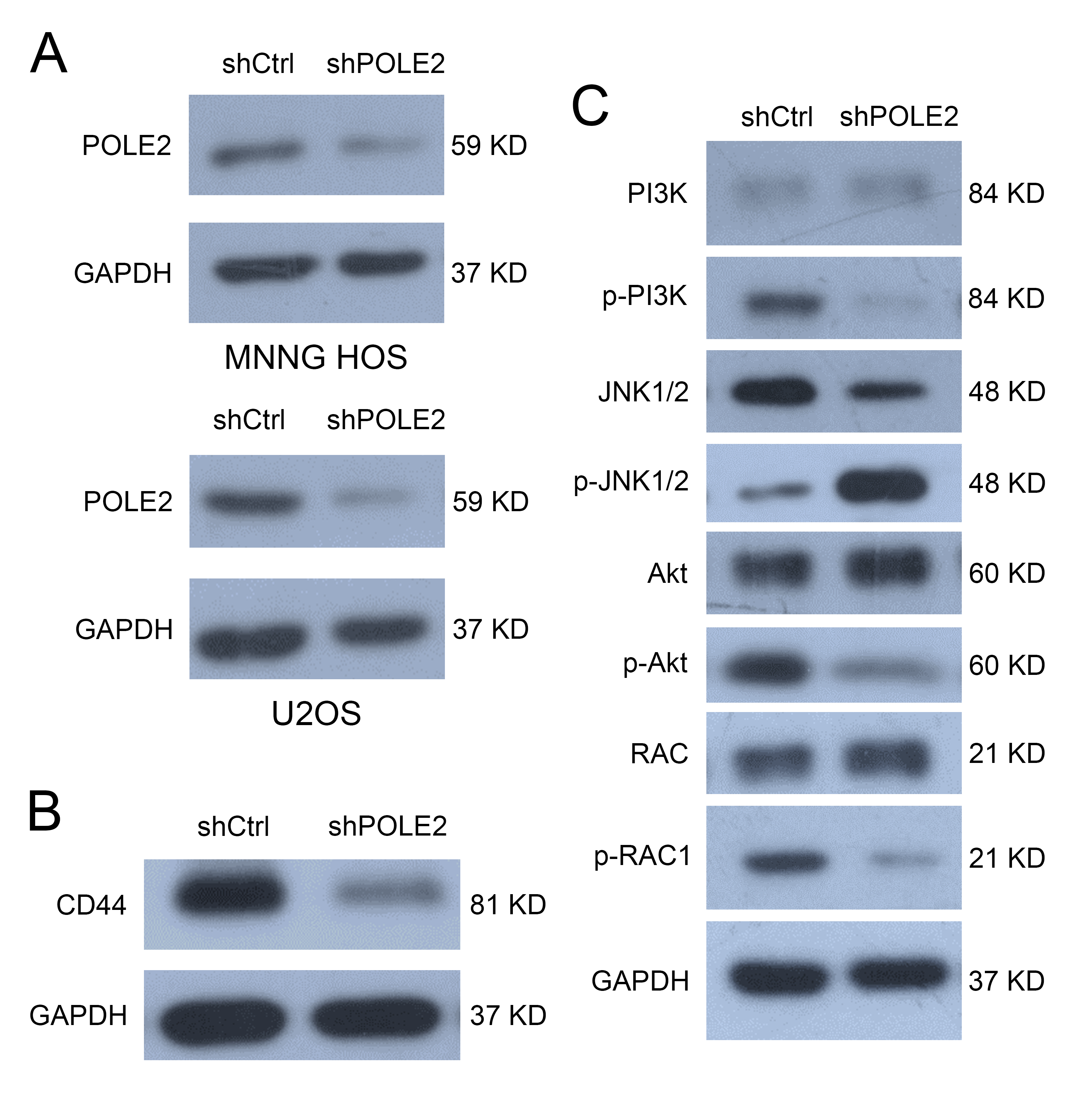


**Supplementary figure 5**

**The original western blots in Supplementary figure 1-2**

(A) The original western blots in Supplementary figure 1D. (B) The original western blots in Supplementary figure 2C. (C) The original western blots in Supplementary figure 2E.


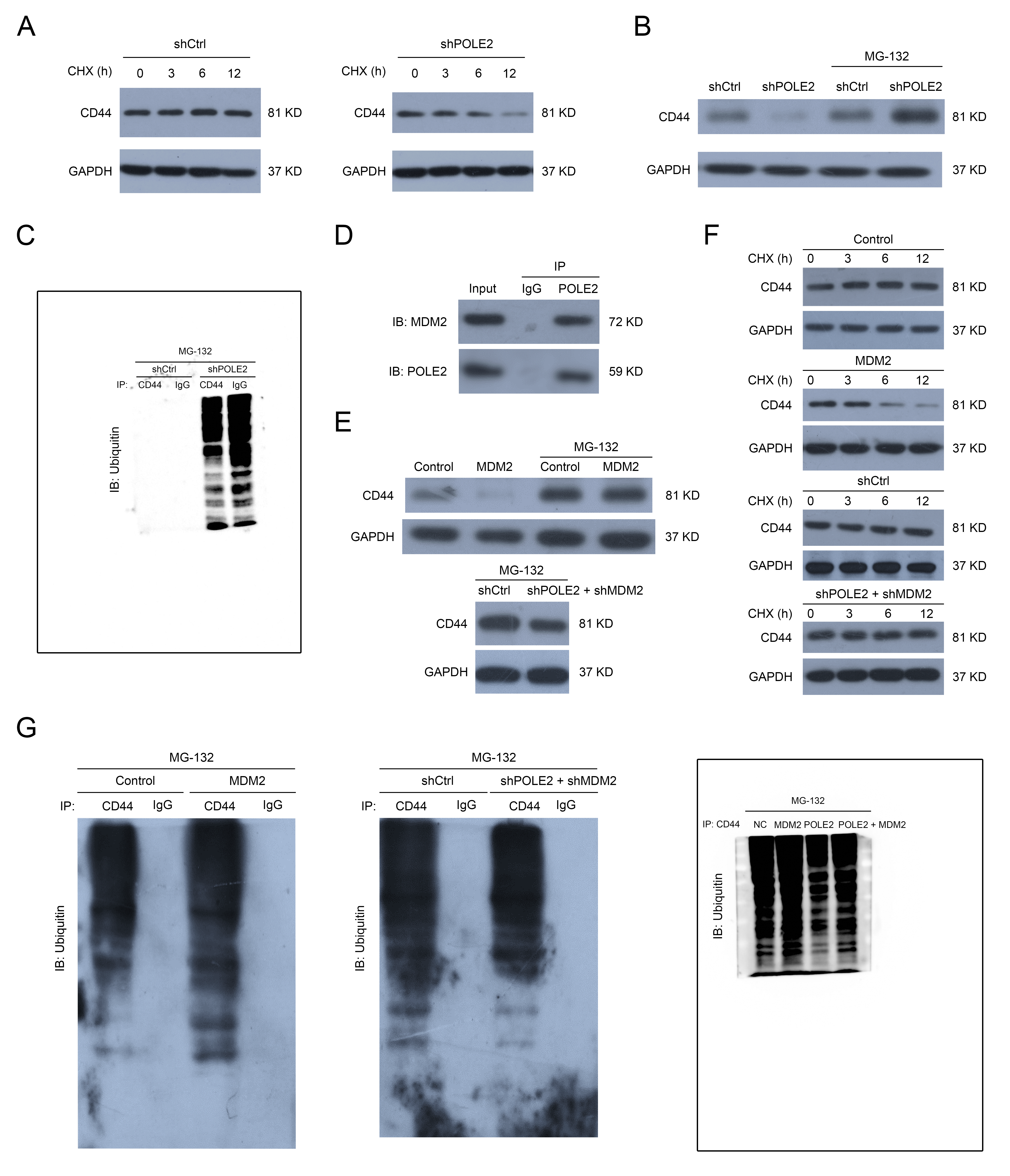


**Supplementary figure 6**

**The original western blots in Figure 4**

(A) The original western blots in Figure 4D. (B) The original western blots in Figure 4E. (C) The original western blots in Figure 4F. (D) The original western blots in Figure 4H. (E) The original western blots in Figure 4I. (F) The original western blots in Figure 4J. (G) The original western blots in Figure 4K.


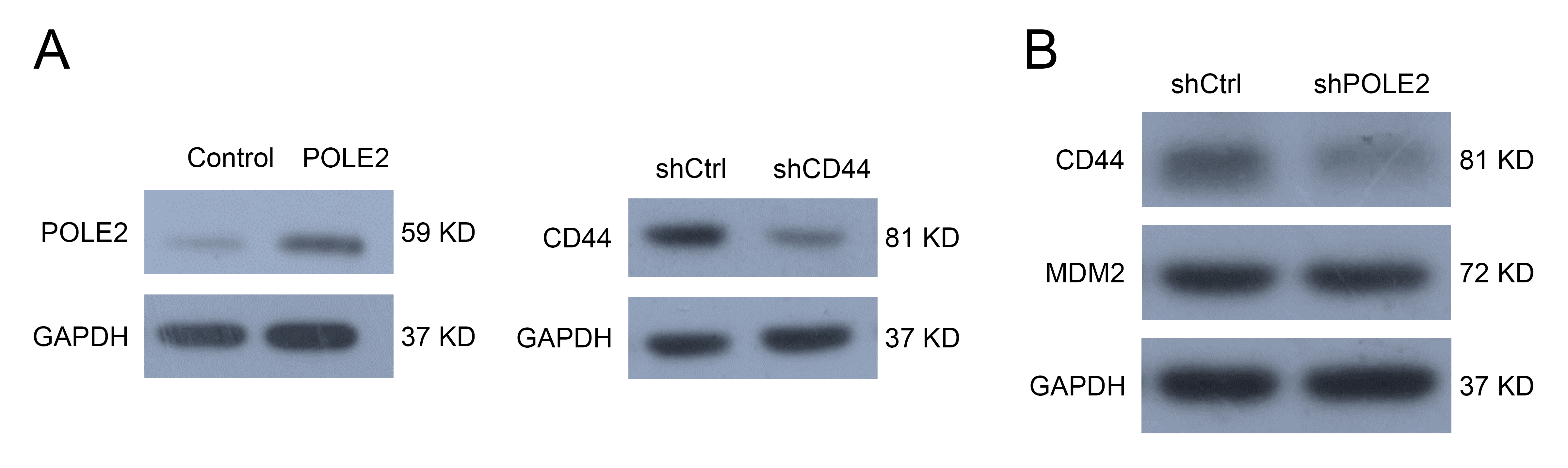


**Supplementary figure 7**

**The original western blots in Supplementary figure 4 and Figure 5**

(A) The original western blots in Supplementary figure 4C. (B) The original western blots in Figure 5F.
