# Supplementary material for: POLE2 promotes osteosarcoma progression by enhancing the stability of CD44
Source: Cell Death Discov. 2024 Apr 16;10:177. doi: 10.1038/s41420-024-01875-x (PMC11021398; doi:10.1038/s41420-024-01875-x)
Supplement: Supplementary file 2 — Supplemental Table 1 [file 41420_2024_1875_MOESM2_ESM.docx]

**Supplementary table 1** The antibodies used in this study.

|  | **Antibody name** | **Dilution ratio** | **Source of antibody species** | **Company** | **Item No** |
| --- | --- | --- | --- | --- | --- |
| **Western Blot or IP** | | | | | |
| The primary antibody | POLE2 | 1:1000 | Rabbit | abcam | ab180214 |
|  | PI3K | 1:1000 | Rabbit | abcam | ab191606 |
|  | P-PI3K | 1:1000 | Rabbit | abcam | ab182651 |
|  | JNK1/2 | 1:1000 | Rabbit | abcam | ab112501 |
|  | P-JNK1/2 | 1:1000 | Rabbit | abcam | ab131499 |
|  | Akt | 1:1000 | Rabbit | CST | 4685 |
|  | p- Akt | 1:1000 | Rabbit | bioss | bs-5193r |
|  | RAC | 1:1000 | Rabbit | abcam | ab180683 |
|  | P-RAC1 | 1:500 | Rabbit | abcam | ab203884 |
|  | CD44 | 1:1000 | Mouse | CST | 3570 |
|  | Ubiquitin | 1:1000 | Rabbit | Proteintech | 10201-2-AP |
|  | MDM2 | 1:2000 | Rabbit | Proteintech | 27883-1-AP |
|  | GAPDH | 1:3000 | Rabbit | Bioworld | AP0063 |
| The second antibody | Goat Anti-Rabbit IgG | 1:3000 | Goat | Beyotime | A0208 |
|  | Goat Anti-Mouse IgG | 1:3000 | Goat | Beyotime | A0216 |
| **Immumohistochemical staining** | | | | | |
| The primary antibody | POLE2 | 1:100 | Rabbit | abcam | ab180214 |
|  | CD44 | 1:200 | Rabbit | abcam | ab157107 |
|  | Ki67 | 1:200 | Rabbit | abcam | ab16667 |
| The second antibody | Goat Anti- Rabbit IgG H&L | 1:400 | Goat | abcam | ab6721 |
