# Supplementary material for: POLE2 promotes osteosarcoma progression by enhancing the stability of CD44
Source: Cell Death Discov. 2024 Apr 16;10:177. doi: 10.1038/s41420-024-01875-x (PMC11021398; doi:10.1038/s41420-024-01875-x)
Supplement: Supplementary file 3 — Supplemental Table 2 [file 41420_2024_1875_MOESM3_ESM.docx]

**Supplementary table 2** The RNAi sequences were designed.

| **Gene** | **ShRNA Sequences** |
| --- | --- |
| shPOLE2 (Pbr10056) | 5′ - GCGATTGTTCTTGGAATGATA - 3′ |
| shPOLE2 (Pbr20910) | 5′ - CTGGAAGATCCTACTGGAACA - 3′ |
| shPOLE2 (Pbr20911) | 5′ - CCTGATGAAAGCGGAAGCAAA - 3′ |
| shCD44 (Pbr10012) | 5′ - AAGCTCTGAGCATCGGATTTG - 3′ |
| shCD44 (Pbr00203) | 5′ - TTGAATATAACCTGCCGCTTT - 3′ |
| shCD44 (Pbr00204) | 5′ - CCGCTGACCTCTGCAAGGCTT - 3′ |
