# Supplementary material for: POLE2 promotes osteosarcoma progression by enhancing the stability of CD44
Source: Cell Death Discov. 2024 Apr 16;10:177. doi: 10.1038/s41420-024-01875-x (PMC11021398; doi:10.1038/s41420-024-01875-x)
Supplement: Supplementary file 4 — Supplemental Table 3 [file 41420_2024_1875_MOESM4_ESM.docx]

**Supplementary table 3** The primer sequences of genes used for qPCR.

| **Gene** | **Forward primer** | **Reverse primer** |
| --- | --- | --- |
| POLE2 | 5′ - TGAGAAGCAACCCTTGTCATC - 3′ | 5′ - TCATCAACAGACTGACTGCATTC - 3′ |
| CXCL8 | 5′ - CTTGGCAGCCTTCCTGATTT - 3′ | 5′ - TGGGGTGGAAAGGTTTGGAG - 3′ |
| CD44 | 5′ - TGGGTTCATAGAAGGGCACG - 3′ | 5′ - ATACTGGGAGGTGTTGGATGTG - 3′ |
| MAPK9 | 5′ - CTCTGCGTCACCCATACATCA - 3′ | 5′ - TCTTTCTTCCAACTGGGCATC - 3′ |
| PIK3CB | 5′ - CTGCGACAGATGAGTGATGAAG - 3′ | 5′ - CCCTATCCTCCGATTACCAAG - 3′ |
| MAPK8 | 5′ - TGCCACAAAATCCTCTTTCCAGG - 3′ | 5′ - AGGTCTGTTTTCAACGTAAGTCCT - 3′ |
| RAP1A | 5′ - CAGTGTATGCTCGAAATCCTGG - 3′ | 5′ - AAACCTTGGCCGTTCTTCAT - 3′ |
| IL6 | 5′ - AAAGGCTGTGCTCTTGGTGA - 3′ | 5′ - TGGGACTCCTGGGAATACTG - 3′ |
| FOS | 5′ - CAGACTACGAGGCGTCATCC - 3′ | 5′ - TCTGCGGGTGAGTGGTAGTA - 3′ |
| ITGA2 | 5′ - GGCGACGAAGTGCTACGAAA - 3′ | 5′ - CCCAAGAACTGCTATGCCAAAC - 3′ |
| MAP3K1 | 5′ - GGAGGAGACAGCCCAGACAATA - 3′ | 5′ - CACCCGGAGCATCACAAAT - 3′ |
| CXCL1 | 5′ - CCCCAAGAACATCCAAAGTGT - 3′ | 5′ - GGATGCAGGATTGAGGCAAG - 3′ |
| FABP4 | 5′ - GGAAAGTCAAGAGCACCATAACC - 3′ | 5′ - CGCATTCCACCACCAGTTTAT - 3′ |
| ANGPT1 | 5′ - CTTCAAGGCTTGGTTACTCGTC - 3′ | 5′ - GAAGGACACTGTTGTTGGTGGTA - 3′ |
| ARNT | 5′ - TCGTGAGCAGCTTTCCACTT - 3′ | 5′ - CCTCATTCGGCAAATAAACG - 3′ |
| APP | 5′ - GACCGAAACGAAAACCACCG - 3′ | 5′ - TCAGCCCCAAAAGAATGCC - 3′ |
| CYR61 | 5′ - TGAAGCGGCTCCCTGTTTT - 3′ | 5′ - GTCTTTGAGCACTGGGACCAT - 3′ |
| IL11 | 5′ - CAGGTGTGCTGACAAGGCT - 3′ | 5′ - GCTCCAGGGTCTTCAGGGAA - 3′ |
| INHBA | 5′ - CAGTGCCAATACCATGAAGAGG - 3′ | 5′ - TCTCTTTCTGGTCCCCACTCT - 3′ |
| MYC | 5′ - TGCTGCCAAGAGGGTCAAGT - 3′ | 5′ - GCTCCGTTTTAGCTCGTTCC - 3′ |
| NFKBIA | 5′ - CTCCATCCTGAAGGCTACCAA - 3′ | 5′ - GCACCCAAGGACACCAAAAG - 3′ |
| GAPDH | 5′ - TGACTTCAACAGCGACACCCA - 3′ | 5′ - CACCCTGTTGCTGTAGCCAAA - 3′ |
