# Supplementary material for: POLE2 promotes osteosarcoma progression by enhancing the stability of CD44
Source: Cell Death Discov. 2024 Apr 16;10:177. doi: 10.1038/s41420-024-01875-x (PMC11021398; doi:10.1038/s41420-024-01875-x)
Supplement: Supplementary file 5 — Supplemental Table 4 [file 41420_2024_1875_MOESM5_ESM.docx]

**Supplementary table 4** The distribution of all proteins in the Human Apoptosis Antibody Array.

|  | **1** | **2** | **3** | **4** | **5** | **6** | **7** | **8** | **9** | **10** | **11** | **12** | **13** | **14** |
| --- | --- | --- | --- | --- | --- | --- | --- | --- | --- | --- | --- | --- | --- | --- |
| **1** | Pos | Pos | Neg | Neg | BLANK | BLANK | Bad | Bax | Bcl-2 | Bcl-w | BID | BIM | Caspase 3 | Caspase 8 |
| **2** | Pos | Pos | Neg | Neg | BLANK | BLANK | Bad | Bax | Bcl-2 | Bcl-w | BID | BIM | Caspase 3 | Caspase 8 |
| **3** | CD40 | CD40L | clAP-2 | cytoC | DR6 | Fas | FasL | BLANK | HSP27 | HSP60 | HSP70 | HTRA | IGF-Ⅰ | IGF-Ⅱ |
| **4** | CD40 | CD40L | clAP-2 | cytoC | DR6 | Fas | FasL | BLANK | HSP27 | HSP60 | HSP70 | HTRA | IGF-Ⅰ | IGF-Ⅱ |
| **5** | IGFBP-1 | IGFBP-2 | IGFBP-3 | IGFBP-4 | IGFBP-5 | IGFBP-6 | IGF-1sR | Livin | p21 | p27 | p53 | SMAC | Survivin | sTNF-R1 |
| **6** | IGFBP-1 | IGFBP-2 | IGFBP-3 | IGFBP-4 | IGFBP-5 | IGFBP-6 | IGF-1sR | Livin | p21 | p27 | p53 | SMAC | Survivin | sTNF-R1 |
| **7** | sTNF-R2 | TNF-α | TNF-β | TRAILR-1 | TRAILR-2 | TRAILR-3 | TRAILR-4 | XIAP | BLANK | BLANK | Neg | Neg | Neg | Pos |
| **8** | sTNF-R2 | TNF-α | TNF-β | TRAILR-1 | TRAILR-2 | TRAILR-3 | TRAILR-4 | XIAP | BLANK | BLANK | Neg | Neg | Neg | Pos |

Note: Protein upregulation is marked in red, protein downregulation is marked in green.
