# Supplementary material for: POLE2 promotes osteosarcoma progression by enhancing the stability of CD44
Source: Cell Death Discov. 2024 Apr 16;10:177. doi: 10.1038/s41420-024-01875-x (PMC11021398; doi:10.1038/s41420-024-01875-x)
Supplement: Supplementary file 6 — Supplemental Table 5 [file 41420_2024_1875_MOESM6_ESM.docx]

**Supplementary table 5** All of the significantly differentially expressed genes in MNNG HOS cells transfected with shPOLE2.

| **Gene Symbol** | **Fold Change** | **logFC** | **P-value** | **FDR** |
| --- | --- | --- | --- | --- |
| REEP5 | -5.589322165 | -2.482673333 | 3.69802E-15 | 2.50694E-12 |
| HOPX | -4.126866206 | -2.045046667 | 1.19738E-14 | 5.67229E-12 |
| POLE2 | -3.285144128 | -1.715956667 | 4.88685E-16 | 5.53924E-13 |
| GPAT3 | -2.662220374 | -1.41263 | 5.91632E-18 | 2.23109E-14 |
| TMCC3 | -2.582914701 | -1.369 | 5.02509E-10 | 2.52573E-08 |
| G3BP1 | -2.546207509 | -1.34835 | 1.44083E-17 | 4.04656E-14 |
| ARMC8 | -2.455879883 | -1.29624 | 5.92535E-12 | 7.49128E-10 |
| HOXA10 | -2.401346071 | -1.263843333 | 7.38562E-14 | 2.45027E-11 |
| HIPK2 | -2.38157613 | -1.251916667 | 6.56919E-18 | 2.23109E-14 |
| PPM1A | -2.319375918 | -1.213736667 | 4.22404E-12 | 5.76684E-10 |
| VEZT | -2.311063125 | -1.208556667 | 3.74283E-10 | 1.98068E-08 |
| GPSM2 | -2.310892262 | -1.20845 | 4.07848E-15 | 2.62888E-12 |
| NAA30 | -2.286007283 | -1.19283 | 4.83463E-16 | 5.53924E-13 |
| EIF1AX | -2.221473092 | -1.151516667 | 2.13045E-14 | 9.20518E-12 |
| NPTX1 | -2.190150484 | -1.13103 | 4.16652E-13 | 9.98923E-11 |
| KCTD3 | -2.164069251 | -1.113746667 | 8.94392E-11 | 6.24629E-09 |
| MMP1 | -2.140285615 | -1.097803333 | 2.82533E-14 | 1.14525E-11 |
| HNRNPA1 | -2.134162455 | -1.09367 | 1.12926E-15 | 9.78621E-13 |
| DPH3 | -2.08266866 | -1.058433333 | 3.19601E-14 | 1.23324E-11 |
| ARHGAP29 | -2.034179043 | -1.024446667 | 3.10282E-11 | 2.71714E-09 |
| CD44 | -2.02749731 | -1.0197 | 5.34069E-15 | 3.05084E-12 |
| STK4 | -2.018434354 | -1.013236667 | 2.73554E-17 | 6.35743E-14 |
| VASP | -2.009254064 | -1.00666 | 1.87167E-14 | 8.26878E-12 |
| PKIB | -2.001224935 | -1.000883333 | 5.02974E-10 | 2.52573E-08 |
| TP53INP1 | -1.990728762 | -0.993296667 | 5.32439E-11 | 4.12106E-09 |
| PUDP | -1.987530041 | -0.990976667 | 1.22915E-12 | 2.21692E-10 |
| KCTD12 | -1.984212674 | -0.988566667 | 2.07136E-11 | 1.99129E-09 |
| SYNE3 | -1.974868602 | -0.981756667 | 7.71102E-11 | 5.56311E-09 |
| LINC00341 | -1.974868602 | -0.981756667 | 7.71102E-11 | 5.56311E-09 |
| RRAGD | -1.956208302 | -0.96806 | 2.08606E-08 | 5.27111E-07 |
| PCMTD1 | -1.94642488 | -0.960826667 | 4.61212E-13 | 1.07943E-10 |
| PPP2R5E | -1.907257981 | -0.9315 | 6.51634E-13 | 1.42465E-10 |
| TRHDE | -1.90657947 | -0.930986667 | 9.04773E-13 | 1.81504E-10 |
| ZNF367 | -1.899101042 | -0.925316667 | 4.57122E-11 | 3.6606E-09 |
| DNER | -1.883526938 | -0.913436667 | 6.39021E-09 | 2.03282E-07 |
| GTF3C6 | -1.87321955 | -0.90552 | 1.33647E-14 | 6.18222E-12 |
| KCTD9 | -1.869470881 | -0.90263 | 1.49731E-11 | 1.55748E-09 |
| ZFAND1 | -1.868227309 | -0.90167 | 1.0263E-13 | 3.12814E-11 |
| ZNF706 | -1.861845466 | -0.896733333 | 4.30158E-11 | 3.50173E-09 |
| AFAP1L1 | -1.8501514 | -0.887643333 | 1.77606E-08 | 4.62469E-07 |
| SOCS5 | -1.841081297 | -0.880553333 | 3.1082E-08 | 7.32384E-07 |
| NETO2 | -1.839537814 | -0.879343333 | 4.0408E-15 | 2.62888E-12 |
| CERS6 | -1.830168736 | -0.871976667 | 2.90274E-14 | 1.16462E-11 |
| GNAI1 | -1.823074266 | -0.866373333 | 1.10935E-09 | 4.80381E-08 |
| PTPRJ | -1.817064964 | -0.86161 | 1.83513E-10 | 1.12567E-08 |
| TPST2 | -1.801426763 | -0.84914 | 2.05909E-09 | 7.98984E-08 |
| SSX2IP | -1.774812558 | -0.827666667 | 1.1028E-11 | 1.2312E-09 |
| BCOR | -1.765397835 | -0.819993333 | 3.5866E-11 | 3.04119E-09 |
| TRDMT1 | -1.760058442 | -0.815623333 | 8.41133E-11 | 5.95901E-09 |
| RPIA | -1.759655894 | -0.815293333 | 7.87343E-14 | 2.55622E-11 |
| IRF4 | -1.758944546 | -0.81471 | 1.49519E-07 | 2.61286E-06 |
| MSANTD3-TMEFF1 | -1.744217558 | -0.80258 | 2.95087E-08 | 7.01483E-07 |
| TMEFF1 | -1.744217558 | -0.80258 | 2.95087E-08 | 7.01483E-07 |
| NEDD4 | -1.737070932 | -0.796656667 | 7.49036E-13 | 1.56473E-10 |
| CPEB2 | -1.728870729 | -0.78983 | 1.36435E-09 | 5.70078E-08 |
| AP1AR | -1.728095962 | -0.789183333 | 1.33336E-10 | 8.60857E-09 |
| IVNS1ABP | -1.724438487 | -0.786126667 | 1.67996E-09 | 6.77845E-08 |
| ITGA6 | -1.717654651 | -0.78044 | 1.04096E-12 | 2.01291E-10 |
| CDH11 | -1.710826351 | -0.774693333 | 1.80427E-07 | 3.01882E-06 |
| TBCEL | -1.704064567 | -0.76898 | 9.12325E-11 | 6.33776E-09 |
| TRIM13 | -1.699350366 | -0.764983333 | 2.13561E-09 | 8.20823E-08 |
| MPP5 | -1.698675171 | -0.76441 | 8.55944E-13 | 1.72957E-10 |
| WNT7B | -1.691011729 | -0.757886667 | 7.37452E-10 | 3.39928E-08 |
| TXNL4A | -1.687226118 | -0.754653333 | 4.93078E-16 | 5.53924E-13 |
| DCAF6 | -1.684729145 | -0.752516667 | 8.03618E-13 | 1.66302E-10 |
| MAP3K2 | -1.684055868 | -0.75194 | 5.75858E-10 | 2.8092E-08 |
| CTHRC1 | -1.680169364 | -0.748606667 | 2.77493E-15 | 1.94835E-12 |
| FAM107B | -1.678326418 | -0.747023333 | 3.52114E-12 | 5.01623E-10 |
| ZW10 | -1.673664086 | -0.74301 | 3.8587E-12 | 5.41858E-10 |
| ACADM | -1.672601003 | -0.742093333 | 1.12987E-09 | 4.86588E-08 |
| PRRX1 | -1.671704673 | -0.74132 | 1.87811E-07 | 3.11059E-06 |
| ASAP2 | -1.670994131 | -0.740706667 | 2.55273E-12 | 3.9516E-10 |
| USP47 | -1.668814193 | -0.738823333 | 1.5244E-08 | 4.10534E-07 |
| ZXDB | -1.668756358 | -0.738773333 | 4.71676E-07 | 6.58852E-06 |
| NET1 | -1.666872012 | -0.737143333 | 5.35385E-15 | 3.05084E-12 |
| NOG | -1.666691011 | -0.736986667 | 2.95463E-10 | 1.61576E-08 |
| ANKRD27 | -1.662856108 | -0.733663333 | 5.7705E-12 | 7.36657E-10 |
| MBOAT1 | -1.662087883 | -0.732996667 | 1.00893E-10 | 6.88716E-09 |
| ZBTB18 | -1.660076816 | -0.73125 | 2.07752E-11 | 1.99234E-09 |
| PDCD6IP | -1.658777059 | -0.73012 | 1.94141E-09 | 7.64873E-08 |
| CCSAP | -1.65419961 | -0.726133333 | 2.22234E-10 | 1.30588E-08 |
| SNAPC1 | -1.649207964 | -0.721773333 | 5.65436E-10 | 2.76522E-08 |
| MAN2A1 | -1.645447504 | -0.71848 | 9.48203E-14 | 2.95892E-11 |
| RCBTB1 | -1.636299308 | -0.710436667 | 7.2175E-10 | 3.34652E-08 |
| TFDP1 | -1.63002791 | -0.704896667 | 9.77896E-13 | 1.94191E-10 |
| PIGK | -1.629470613 | -0.704403333 | 1.11324E-09 | 4.81534E-08 |
| AKAP12 | -1.626502831 | -0.701773333 | 3.56183E-14 | 1.34998E-11 |
| CNOT6 | -1.624471012 | -0.69997 | 9.93807E-12 | 1.14928E-09 |
| CDC42SE2 | -1.623142879 | -0.69879 | 2.43274E-11 | 2.26348E-09 |
| SRSF1 | -1.621407438 | -0.697246667 | 5.15369E-12 | 6.73215E-10 |
| LINC00657 | -1.620081809 | -0.696066667 | 4.98866E-15 | 3.01768E-12 |
| PIK3CB | -1.618293555 | -0.694473333 | 6.39955E-11 | 4.78847E-09 |
| KLHL4 | -1.616387762 | -0.692773333 | 4.67846E-08 | 1.01631E-06 |
| PCMT1 | -1.612091167 | -0.688933333 | 7.47813E-14 | 2.45027E-11 |
| ARFGEF3 | -1.60767598 | -0.684976667 | 5.38414E-07 | 7.32776E-06 |
| TRMT1L | -1.60535608 | -0.682893333 | 8.429E-07 | 1.06395E-05 |
| SIKE1 | -1.604147349 | -0.681806667 | 1.37513E-11 | 1.45965E-09 |
| FAM169A | -1.604065811 | -0.681733333 | 2.18445E-08 | 5.48821E-07 |
| CCNJ | -1.60357667 | -0.681293333 | 1.1266E-06 | 1.35755E-05 |
| PODXL | -1.603565555 | -0.681283333 | 1.81538E-12 | 3.00312E-10 |
| ETV5 | -1.602861755 | -0.68065 | 2.23945E-10 | 1.30643E-08 |
| ARL6IP1 | -1.601455082 | -0.679383333 | 2.20414E-11 | 2.09335E-09 |
| ATP6V1H | -1.600238196 | -0.678286667 | 1.2434E-13 | 3.70373E-11 |
| RAD17 | -1.597176038 | -0.675523333 | 3.01015E-12 | 4.43281E-10 |
| GPR180 | -1.594469683 | -0.673076667 | 2.44347E-12 | 3.79742E-10 |
| PDP2 | -1.588233833 | -0.667423333 | 2.10672E-06 | 2.29076E-05 |
| BNIP3L | -1.588204476 | -0.667396667 | 2.33906E-13 | 6.29928E-11 |
| CYSTM1 | -1.585857711 | -0.665263333 | 2.0267E-13 | 5.61181E-11 |
| OTUD4 | -1.582559783 | -0.66226 | 1.161E-12 | 2.16073E-10 |
| ARRDC3 | -1.580046116 | -0.659966667 | 3.98196E-06 | 3.92595E-05 |
| SMYD2 | -1.577040816 | -0.65722 | 9.5085E-11 | 6.54755E-09 |
| RAP1A | -1.576916934 | -0.657106667 | 3.2662E-09 | 1.16643E-07 |
| PLGLB2 | -1.575798791 | -0.656083333 | 1.93014E-06 | 2.13297E-05 |
| PLGLA | -1.575798791 | -0.656083333 | 1.93014E-06 | 2.13297E-05 |
| PLGLB1 | -1.575798791 | -0.656083333 | 1.93014E-06 | 2.13297E-05 |
| MED10 | -1.574801509 | -0.65517 | 1.04436E-12 | 2.01291E-10 |
| LIFR | -1.572986912 | -0.653506667 | 6.64346E-07 | 8.71295E-06 |
| DCP2 | -1.570989277 | -0.651673333 | 1.54468E-11 | 1.58664E-09 |
| ITGA2 | -1.570092983 | -0.65085 | 3.23352E-12 | 4.70885E-10 |
| SESN1 | -1.568754938 | -0.64962 | 1.57601E-07 | 2.72264E-06 |
| COX15 | -1.566574449 | -0.647613333 | 7.20536E-09 | 2.22131E-07 |
| CASD1 | -1.56603161 | -0.647113333 | 2.8968E-11 | 2.56646E-09 |
| ROBO4 | -1.565517895 | -0.64664 | 2.03958E-09 | 7.93217E-08 |
| AASDHPPT | -1.563681479 | -0.644946667 | 1.85214E-12 | 3.02176E-10 |
| LOXL2 | -1.561266335 | -0.642716667 | 7.90614E-15 | 4.20083E-12 |
| ELMOD2 | -1.560898435 | -0.642376667 | 1.37396E-10 | 8.84171E-09 |
| LRRC20 | -1.560141266 | -0.641676667 | 1.12838E-10 | 7.50708E-09 |
| CCDC109B | -1.559456525 | -0.641043333 | 9.13707E-07 | 1.13834E-05 |
| SLC12A2 | -1.556461617 | -0.63827 | 6.59714E-09 | 2.08515E-07 |
| NEXN | -1.555242986 | -0.63714 | 0.000127502 | 0.000743478 |
| LARP6 | -1.555217833 | -0.637116667 | 2.72078E-10 | 1.5025E-08 |
| SLAIN1 | -1.553863742 | -0.63586 | 5.21105E-08 | 1.10813E-06 |
| SCYL2 | -1.550786413 | -0.633 | 2.33682E-10 | 1.3512E-08 |
| ARNT | -1.550141593 | -0.6324 | 3.03737E-08 | 7.18138E-07 |
| KCNN3 | -1.548816972 | -0.631166667 | 1.02404E-05 | 8.73219E-05 |
| CHAC2 | -1.548652368 | -0.631013333 | 1.07166E-10 | 7.21518E-09 |
| IRAK2 | -1.548105009 | -0.630503333 | 2.42681E-08 | 5.96002E-07 |
| ARF6 | -1.547060911 | -0.62953 | 3.45199E-13 | 8.62215E-11 |
| ANXA5 | -1.545896073 | -0.628443333 | 2.56633E-11 | 2.3576E-09 |
| LRRC8C | -1.544525118 | -0.627163333 | 1.2879E-08 | 3.56612E-07 |
| ANKRD13C | -1.544332425 | -0.626983333 | 2.8827E-08 | 6.8819E-07 |
| DNAJC9 | -1.544246792 | -0.626903333 | 1.79044E-06 | 2.00393E-05 |
| RBAK | -1.541010473 | -0.623876667 | 6.81855E-09 | 2.13308E-07 |
| SWAP70 | -1.540230923 | -0.623146667 | 3.28956E-10 | 1.76697E-08 |
| ORC2 | -1.536197102 | -0.619363333 | 1.27593E-09 | 5.37741E-08 |
| LIMS1 | -1.534260369 | -0.617543333 | 4.1694E-11 | 3.40825E-09 |
| TMTC4 | -1.532485404 | -0.615873333 | 5.1133E-07 | 7.02598E-06 |
| KLF4 | -1.531416458 | -0.614866667 | 1.08585E-10 | 7.28577E-09 |
| FAM45BP | -1.530613469 | -0.61411 | 8.91031E-08 | 1.71653E-06 |
| FAM45A | -1.530613469 | -0.61411 | 8.91031E-08 | 1.71653E-06 |
| NTN4 | -1.525786743 | -0.609553333 | 1.30007E-07 | 2.33094E-06 |
| RBM22 | -1.522638364 | -0.606573333 | 5.89149E-12 | 7.49128E-10 |
| IFT80 | -1.520694129 | -0.60473 | 6.59155E-11 | 4.89931E-09 |
| PAG1 | -1.519584253 | -0.603676667 | 3.30744E-06 | 3.35947E-05 |
| ANP32A | -1.519457862 | -0.603556667 | 1.54001E-09 | 6.28779E-08 |
| TMX1 | -1.518731323 | -0.602866667 | 1.97689E-12 | 3.19874E-10 |
| SH3PXD2A | -1.518064756 | -0.602233333 | 3.38458E-13 | 8.53066E-11 |
| FOXF1 | -1.517580802 | -0.601773333 | 1.13139E-10 | 7.51436E-09 |
| PPP4R2 | -1.517198657 | -0.60141 | 1.18003E-08 | 3.31486E-07 |
| PPM1B | -1.515492452 | -0.599786667 | 1.03881E-12 | 2.01291E-10 |
| PYGB | -1.515467941 | -0.599763333 | 7.11633E-13 | 1.52069E-10 |
| PM20D2 | -1.51503032 | -0.599346667 | 6.59053E-09 | 2.08474E-07 |
| MGAT2 | -1.512169645 | -0.59662 | 1.55387E-13 | 4.45962E-11 |
| BCL11A | -1.511471036 | -0.595953333 | 1.3383E-05 | 0.000109784 |
| GNAI3 | -1.510319036 | -0.594853333 | 9.26349E-11 | 6.40125E-09 |
| ADAM8 | -1.508202347 | -0.59283 | 1.6637E-08 | 4.38438E-07 |
| AGFG1 | -1.507878306 | -0.59252 | 7.92318E-11 | 5.68199E-09 |
| C10orf88 | -1.506499297 | -0.5912 | 9.01731E-08 | 1.73545E-06 |
| ZNF286A | -1.503981339 | -0.588786667 | 1.54789E-05 | 0.000124334 |
| ZNF286B | -1.503981339 | -0.588786667 | 1.54789E-05 | 0.000124334 |
| AMD1P3 | -1.502324708 | -0.587196667 | 1.73005E-08 | 4.52587E-07 |
| AMD1 | -1.502324708 | -0.587196667 | 1.73005E-08 | 4.52587E-07 |
| RCOR1 | -1.497974779 | -0.583013333 | 1.63566E-11 | 1.65553E-09 |
| ZC3HAV1L | -1.497898637 | -0.58294 | 1.8985E-09 | 7.51733E-08 |
| SRSF10 | -1.49769792 | -0.582746667 | 1.35529E-09 | 5.67505E-08 |
| MAF | -1.497037126 | -0.58211 | 1.74843E-06 | 1.96587E-05 |
| MDFIC | -1.496425027 | -0.58152 | 1.22931E-06 | 1.45631E-05 |
| RAB39B | -1.496259078 | -0.58136 | 1.7343E-07 | 2.93295E-06 |
| RAP2B | -1.495740604 | -0.58086 | 3.94626E-05 | 0.000275063 |
| RBPJ | -1.491978418 | -0.577226667 | 4.63944E-11 | 3.70016E-09 |
| BMF | -1.490545071 | -0.57584 | 1.14898E-09 | 4.93197E-08 |
| LNX2 | -1.490304018 | -0.575606667 | 2.74397E-09 | 1.00926E-07 |
| PGBD1 | -1.48955356 | -0.57488 | 6.54024E-07 | 8.6034E-06 |
| LURAP1L | -1.48770313 | -0.573086667 | 1.24431E-05 | 0.000103239 |
| FBXO45 | -1.487208238 | -0.572606667 | 3.2868E-07 | 4.89336E-06 |
| ZCCHC14 | -1.485916795 | -0.571353333 | 8.20339E-11 | 5.83272E-09 |
| SORL1 | -1.484931794 | -0.570396667 | 9.68247E-06 | 8.3287E-05 |
| TDP2 | -1.483875446 | -0.56937 | 2.25862E-08 | 5.62423E-07 |
| FAR1 | -1.482734202 | -0.56826 | 5.03284E-09 | 1.68845E-07 |
| EIF4EBP2 | -1.482199866 | -0.56774 | 1.00555E-06 | 1.23284E-05 |
| BFAR | -1.481689687 | -0.567243333 | 1.88755E-11 | 1.85287E-09 |
| HKDC1 | -1.480057611 | -0.565653333 | 1.24159E-06 | 1.46821E-05 |
| NOP9 | -1.47979432 | -0.565396667 | 3.81181E-08 | 8.59383E-07 |
| UHRF1BP1 | -1.479722522 | -0.565326667 | 2.78705E-12 | 4.19863E-10 |
| CNKSR3 | -1.479678077 | -0.565283333 | 6.72732E-10 | 3.16401E-08 |
| MRPL48 | -1.479626796 | -0.565233333 | 1.33581E-12 | 2.33434E-10 |
| ARMCX3 | -1.478509315 | -0.564143333 | 6.77717E-07 | 8.85876E-06 |
| ARHGAP5 | -1.477915037 | -0.563563333 | 1.5506E-06 | 1.78009E-05 |
| ADAM12 | -1.47788089 | -0.56353 | 1.09E-06 | 1.31951E-05 |
| KDSR | -1.477648713 | -0.563303333 | 1.54125E-08 | 4.13372E-07 |
| SLC25A36 | -1.477532639 | -0.56319 | 3.31543E-12 | 4.81032E-10 |
| THBD | -1.477518984 | -0.563176667 | 1.12868E-06 | 1.35964E-05 |
| DNAJC25-GNG10 | -1.475386902 | -0.561093333 | 1.13355E-14 | 5.64177E-12 |
| GNG10 | -1.475386902 | -0.561093333 | 1.13355E-14 | 5.64177E-12 |
| SHC2 | -1.474436135 | -0.560163333 | 4.41913E-06 | 4.28604E-05 |
| KIAA0319L | -1.472546644 | -0.558313333 | 4.31775E-10 | 2.23675E-08 |
| SYT1 | -1.470693555 | -0.556496667 | 7.26677E-07 | 9.41113E-06 |
| ZNF566 | -1.47048629 | -0.556293333 | 1.13451E-09 | 4.87994E-08 |
| NT5C1B-RDH14 | -1.470248481 | -0.55606 | 3.01406E-09 | 1.09025E-07 |
| RDH14 | -1.470248481 | -0.55606 | 3.01406E-09 | 1.09025E-07 |
| VGF | -1.469681292 | -0.555503333 | 1.72452E-13 | 4.87816E-11 |
| SPAG9 | -1.469436823 | -0.555263333 | 5.29559E-09 | 1.7512E-07 |
| CAPN2 | -1.468299898 | -0.554146667 | 6.02552E-09 | 1.94354E-07 |
| IL6R | -1.465957556 | -0.551843333 | 7.43993E-08 | 1.48644E-06 |
| SLCO4A1 | -1.465530846 | -0.551423333 | 4.56008E-12 | 6.09856E-10 |
| FAM208B | -1.46357837 | -0.5495 | 1.47114E-09 | 6.06965E-08 |
| TNFRSF19 | -1.462939391 | -0.54887 | 4.2863E-09 | 1.46169E-07 |
| NT5DC3 | -1.460335647 | -0.5463 | 1.34892E-08 | 3.72459E-07 |
| PCGF6 | -1.456554819 | -0.54256 | 3.4517E-08 | 7.95997E-07 |
| PPP1R15B | -1.455293357 | -0.54131 | 9.21761E-11 | 6.38076E-09 |
| LUZP1 | -1.454352178 | -0.540376667 | 7.65961E-09 | 2.33464E-07 |
| HEY1 | -1.452709935 | -0.538746667 | 3.59584E-09 | 1.26012E-07 |
| C12orf76 | -1.452428019 | -0.538466667 | 1.537E-08 | 4.12897E-07 |
| HS2ST1 | -1.451508815 | -0.537553333 | 1.22917E-11 | 1.33878E-09 |
| WNT5B | -1.449571672 | -0.535626667 | 2.09037E-09 | 8.07381E-08 |
| TRIM25 | -1.449568322 | -0.535623333 | 6.36337E-11 | 4.77483E-09 |
| EXOC5 | -1.449266924 | -0.535323333 | 6.98986E-06 | 6.29055E-05 |
| OXTR | -1.448597376 | -0.534656667 | 2.38685E-05 | 0.000178884 |
| SGCB | -1.446764396 | -0.53283 | 9.11243E-07 | 1.13635E-05 |
| HIPK1 | -1.446069274 | -0.532136667 | 1.04506E-08 | 2.98623E-07 |
| CDC42 | -1.445210859 | -0.53128 | 4.91752E-08 | 1.05599E-06 |
| MYLK | -1.443218763 | -0.52929 | 3.61763E-08 | 8.24588E-07 |
| TEX261 | -1.443062048 | -0.529133333 | 1.41891E-10 | 9.10114E-09 |
| SCD | -1.442695335 | -0.528766667 | 6.93116E-15 | 3.83358E-12 |
| KLHL2 | -1.442188757 | -0.52826 | 1.56972E-07 | 2.71296E-06 |
| PRDM8 | -1.441895556 | -0.527966667 | 5.52172E-10 | 2.71725E-08 |
| TTL | -1.441562446 | -0.527633333 | 5.76921E-10 | 2.8109E-08 |
| RBMX2 | -1.441266043 | -0.527336667 | 1.96319E-09 | 7.71137E-08 |
| S100P | -1.43990803 | -0.525976667 | 1.17949E-07 | 2.14997E-06 |
| RFC5 | -1.439621946 | -0.52569 | 8.98485E-12 | 1.0673E-09 |
| PDGFA | -1.43951551 | -0.525583333 | 5.43372E-07 | 7.37482E-06 |
| ANKRD36B | -1.439212877 | -0.52528 | 5.13375E-06 | 4.85109E-05 |
| ANKRD36 | -1.439212877 | -0.52528 | 5.13375E-06 | 4.85109E-05 |
| ADO | -1.439046622 | -0.525113333 | 1.24817E-10 | 8.13875E-09 |
| SPRED1 | -1.439026673 | -0.525093333 | 3.36759E-08 | 7.79342E-07 |
| EBNA1BP2 | -1.438272128 | -0.524336667 | 7.06566E-11 | 5.18311E-09 |
| IGFBP1 | -1.437172598 | -0.523233333 | 3.78601E-06 | 3.75535E-05 |
| KIFAP3 | -1.436887057 | -0.522946667 | 1.91718E-09 | 7.56627E-08 |
| KRT80 | -1.436784143 | -0.522843333 | 3.91571E-08 | 8.79278E-07 |
| PHOSPHO2-KLHL23 | -1.43593124 | -0.521986667 | 2.906E-08 | 6.92072E-07 |
| KLHL23 | -1.43593124 | -0.521986667 | 2.906E-08 | 6.92072E-07 |
| TIMP3 | -1.435576289 | -0.52163 | 6.76114E-11 | 4.9783E-09 |
| VWA9 | -1.435500003 | -0.521553333 | 1.75264E-11 | 1.73582E-09 |
| STEAP2 | -1.435171686 | -0.521223333 | 9.98454E-08 | 1.88108E-06 |
| PDHA1 | -1.43466775 | -0.520716667 | 1.44361E-10 | 9.2295E-09 |
| KBTBD7 | -1.433710097 | -0.519753333 | 6.58869E-07 | 8.66057E-06 |
| ELMO1 | -1.432799428 | -0.518836667 | 5.2021E-05 | 0.000348155 |
| PAQR3 | -1.431909189 | -0.51794 | 3.44641E-08 | 7.95242E-07 |
| CFL2 | -1.430391432 | -0.51641 | 2.92405E-11 | 2.58361E-09 |
| CHAMP1 | -1.429456449 | -0.515466667 | 2.96734E-11 | 2.61599E-09 |
| SUB1 | -1.429436633 | -0.515446667 | 6.13941E-09 | 1.9717E-07 |
| TIGAR | -1.429354068 | -0.515363333 | 4.8202E-07 | 6.69898E-06 |
| FOXK2 | -1.429099797 | -0.515106667 | 1.03995E-10 | 7.03785E-09 |
| MARK3 | -1.427357446 | -0.513346667 | 2.67013E-08 | 6.47268E-07 |
| ITGAV | -1.427242024 | -0.51323 | 2.91525E-06 | 3.01882E-05 |
| CIPC | -1.426866144 | -0.51285 | 3.34063E-08 | 7.74267E-07 |
| PPP2CB | -1.425413013 | -0.51138 | 2.56135E-09 | 9.54595E-08 |
| CCNE2 | -1.425192372 | -0.511156667 | 2.39162E-06 | 2.55394E-05 |
| ZC3H14 | -1.423859374 | -0.509806667 | 1.93911E-10 | 1.17118E-08 |
| CBR3 | -1.423819896 | -0.509766667 | 1.45757E-08 | 3.96063E-07 |
| NAGS | -1.422001842 | -0.507923333 | 2.01827E-10 | 1.20602E-08 |
| ZCCHC4 | -1.421574788 | -0.50749 | 0.000102355 | 0.00061499 |
| CEMIP | -1.421216819 | -0.507126667 | 1.60925E-05 | 0.000128502 |
| PRPS2 | -1.420104077 | -0.505996667 | 6.21312E-11 | 4.67996E-09 |
| CSGALNACT2 | -1.41983833 | -0.505726667 | 5.56057E-13 | 1.25653E-10 |
| TBXAS1 | -1.419490637 | -0.505373333 | 2.03966E-10 | 1.21511E-08 |
| BIVM | -1.418444792 | -0.50431 | 3.07058E-07 | 4.63998E-06 |
| WASF1 | -1.416358685 | -0.502186667 | 3.90575E-09 | 1.35185E-07 |
| TRIOBP | -1.41489991 | -0.5007 | 4.51173E-07 | 6.33786E-06 |
| CDKN2AIP | -1.414834529 | -0.500633333 | 6.09645E-05 | 0.000397589 |
| PDCL | -1.414099204 | -0.499883333 | 2.62083E-06 | 2.75236E-05 |
| ANP32E | -1.412218512 | -0.497963333 | 7.46005E-14 | 2.45027E-11 |
| FAM179B | -1.411442151 | -0.49717 | 1.04442E-07 | 1.94348E-06 |
| TCEA1 | -1.411070432 | -0.49679 | 4.39765E-12 | 5.94196E-10 |
| DYRK2 | -1.410998708 | -0.496716667 | 3.2885E-07 | 4.89404E-06 |
| H1F0 | -1.41086179 | -0.496576667 | 1.52577E-09 | 6.23936E-08 |
| SYNCRIP | -1.410780298 | -0.496493333 | 0.000166951 | 0.000935237 |
| TNRC6B | -1.410454376 | -0.49616 | 5.28911E-07 | 7.21341E-06 |
| ETV1 | -1.410082917 | -0.49578 | 1.76436E-06 | 1.98008E-05 |
| CCDC88C | -1.408663152 | -0.494326667 | 3.63647E-10 | 1.94534E-08 |
| PLS1 | -1.408572023 | -0.494233333 | 1.22591E-09 | 5.2166E-08 |
| TAF5L | -1.408513443 | -0.494173333 | 2.32371E-08 | 5.76078E-07 |
| MYO1B | -1.408448358 | -0.494106667 | 3.12867E-06 | 3.20438E-05 |
| CHORDC1 | -1.407843205 | -0.493486667 | 6.42099E-07 | 8.47488E-06 |
| FAM111B | -1.407316349 | -0.492946667 | 0.000153126 | 0.000869799 |
| UEVLD | -1.407121267 | -0.492746667 | 3.86726E-05 | 0.000270564 |
| AURKAPS1 | -1.407030238 | -0.492653333 | 3.50625E-11 | 2.98403E-09 |
| AURKA | -1.407030238 | -0.492653333 | 3.50625E-11 | 2.98403E-09 |
| RAB3GAP2 | -1.407030238 | -0.492653333 | 3.50625E-11 | 2.98403E-09 |
| TBC1D26 | -1.404970657 | -0.49054 | 1.03683E-05 | 8.82975E-05 |
| MEF2A | -1.404409182 | -0.489963333 | 3.54361E-09 | 1.24737E-07 |
| PHF5A | -1.404399447 | -0.489953333 | 0.000161961 | 0.000911689 |
| DDX52 | -1.403938754 | -0.48948 | 7.31528E-05 | 0.000464293 |
| IMPDH1 | -1.403121557 | -0.48864 | 1.22207E-12 | 2.21431E-10 |
| SLC1A4 | -1.402940023 | -0.488453333 | 1.52838E-08 | 4.11323E-07 |
| ZBTB1 | -1.402427962 | -0.487926667 | 0.001226128 | 0.005149553 |
| SAMHD1 | -1.401349359 | -0.486816667 | 3.17036E-06 | 3.2378E-05 |
| NUDCD1 | -1.401054749 | -0.486513333 | 3.60319E-07 | 5.26669E-06 |
| SOX21 | -1.400867009 | -0.48632 | 1.93335E-06 | 2.13532E-05 |
| NDRG3 | -1.400566029 | -0.48601 | 9.58479E-05 | 0.00058203 |
| AP3M2 | -1.39986076 | -0.485283333 | 3.10765E-09 | 1.11895E-07 |
| NCAPG2 | -1.398448055 | -0.483826667 | 9.07888E-09 | 2.68602E-07 |
| CEP63 | -1.398137904 | -0.483506667 | 5.80486E-06 | 5.38178E-05 |
| HNRNPU | -1.397873038 | -0.483233333 | 1.07647E-06 | 1.30637E-05 |
| STK32B | -1.396775346 | -0.4821 | 1.29097E-09 | 5.42884E-08 |
| ABCE1 | -1.396594633 | -0.481913333 | 5.22153E-11 | 4.06545E-09 |
| LYST | -1.395233579 | -0.480506667 | 4.65119E-08 | 1.01207E-06 |
| RAB21 | -1.394804896 | -0.480063333 | 1.50347E-09 | 6.1771E-08 |
| MT1P3 | -1.393638772 | -0.478856667 | 6.49702E-11 | 4.83819E-09 |
| MT1A | -1.393638772 | -0.478856667 | 6.49702E-11 | 4.83819E-09 |
| MT1JP | -1.393590473 | -0.478806667 | 1.30359E-11 | 1.40427E-09 |
| MT1M | -1.393590473 | -0.478806667 | 1.30359E-11 | 1.40427E-09 |
| YWHAG | -1.393400513 | -0.47861 | 1.08547E-10 | 7.28577E-09 |
| UBE4A | -1.392473623 | -0.47765 | 3.83926E-09 | 1.33236E-07 |
| VAPA | -1.392168014 | -0.477333333 | 2.01045E-05 | 0.000155028 |
| PAFAH1B1 | -1.391621301 | -0.476766667 | 0.001027821 | 0.004426383 |
| MAP3K1 | -1.391550565 | -0.476693333 | 1.10052E-08 | 3.12203E-07 |
| ZNF287 | -1.390277942 | -0.475373333 | 0.000180411 | 0.000999518 |
| DUSP14 | -1.390210487 | -0.475303333 | 4.29509E-10 | 2.22795E-08 |
| DENR | -1.389715917 | -0.47479 | 1.91538E-11 | 1.8734E-09 |
| ZIC5 | -1.38937239 | -0.474433333 | 5.03595E-05 | 0.000339172 |
| PITHD1 | -1.389166957 | -0.47422 | 1.41133E-07 | 2.48956E-06 |
| NRG1 | -1.388749764 | -0.473786667 | 2.3578E-09 | 8.90551E-08 |
| SEC22A | -1.386771411 | -0.47173 | 1.32436E-05 | 0.000108868 |
| SAE1 | -1.386697718 | -0.471653333 | 4.59084E-13 | 1.07943E-10 |
| TRIM8 | -1.386226817 | -0.471163333 | 1.46928E-10 | 9.29967E-09 |
| SAAL1 | -1.386105114 | -0.471036667 | 1.56169E-10 | 9.7933E-09 |
| RGCC | -1.38599623 | -0.470923333 | 1.9189E-10 | 1.16254E-08 |
| ARMT1 | -1.385858537 | -0.47078 | 3.20455E-07 | 4.79451E-06 |
| TBC1D13 | -1.385704849 | -0.47062 | 1.49425E-07 | 2.61273E-06 |
| XPO5 | -1.384907865 | -0.46979 | 3.58845E-08 | 8.2002E-07 |
| CFLAR | -1.384565527 | -0.469433333 | 7.75866E-06 | 6.89029E-05 |
| CCDC112 | -1.384450367 | -0.469313333 | 1.31036E-07 | 2.34617E-06 |
| DDX5 | -1.384338415 | -0.469196667 | 1.97274E-07 | 3.23192E-06 |
| SEH1L | -1.384271248 | -0.469126667 | 1.6615E-10 | 1.0288E-08 |
| RPF2 | -1.384120934 | -0.46897 | 0.00030759 | 0.001577222 |
| ZBTB14 | -1.383718045 | -0.46855 | 4.82892E-05 | 0.000326908 |
| STX12 | -1.383356823 | -0.468173333 | 1.61205E-07 | 2.77149E-06 |
| SUCLA2 | -1.383331254 | -0.468146667 | 2.78525E-08 | 6.69446E-07 |
| LSMEM1 | -1.383203412 | -0.468013333 | 1.76317E-05 | 0.000139024 |
| HNRNPR | -1.383065997 | -0.46787 | 3.92239E-06 | 3.87304E-05 |
| FOXD3 | -1.383024455 | -0.467826667 | 0.000238078 | 0.001269549 |
| SPTBN1 | -1.383008478 | -0.46781 | 2.76073E-08 | 6.65537E-07 |
| KIAA1804 | -1.382909423 | -0.467706667 | 9.24118E-05 | 0.000565356 |
| SLC14A1 | -1.382768841 | -0.46756 | 4.51243E-12 | 6.07617E-10 |
| RASA2 | -1.382225819 | -0.466993333 | 1.4151E-05 | 0.000115174 |
| ZNF622 | -1.381497866 | -0.466233333 | 5.02962E-12 | 6.63623E-10 |
| CHN2 | -1.380709682 | -0.46541 | 0.000264122 | 0.001385592 |
| LOC101928168 | -1.380709682 | -0.46541 | 0.000264122 | 0.001385592 |
| CRKL | -1.380524668 | -0.465216667 | 1.26927E-09 | 5.3663E-08 |
| GOSR1 | -1.380457686 | -0.465146667 | 2.27052E-06 | 2.4372E-05 |
| FAM129A | -1.38025676 | -0.464936667 | 8.10816E-11 | 5.7948E-09 |
| PCMTD2 | -1.379925136 | -0.46459 | 3.28196E-09 | 1.16993E-07 |
| ZNF780A | -1.379319491 | -0.463956667 | 0.000347288 | 0.001753068 |
| CCP110 | -1.379144223 | -0.463773333 | 7.30925E-07 | 9.45681E-06 |
| EMP1 | -1.379080494 | -0.463706667 | 2.95973E-06 | 3.05363E-05 |
| ARHGAP22 | -1.378761896 | -0.463373333 | 0.002202175 | 0.008474828 |
| ANTXR2 | -1.378099447 | -0.46268 | 5.25203E-09 | 1.74265E-07 |
| MYO10 | -1.377847927 | -0.462416667 | 9.40425E-10 | 4.19051E-08 |
| MTSS1 | -1.377564624 | -0.46212 | 3.14049E-05 | 0.000225742 |
| CEP170 | -1.37723683 | -0.461776667 | 3.15429E-06 | 3.22642E-05 |
| CEP170P1 | -1.37723683 | -0.461776667 | 3.15429E-06 | 3.22642E-05 |
| MGAT4B | -1.377170007 | -0.461706667 | 2.94567E-12 | 4.35829E-10 |
| ENAH | -1.376619642 | -0.46113 | 1.99089E-05 | 0.000153882 |
| DNAJC13 | -1.376549669 | -0.461056667 | 5.96675E-08 | 1.24262E-06 |
| SNX24 | -1.376190319 | -0.46068 | 0.000138715 | 0.000799491 |
| ST20 | -1.375678487 | -0.460143333 | 1.25465E-06 | 1.48054E-05 |
| SNX30 | -1.375414697 | -0.459866667 | 1.29039E-06 | 1.51906E-05 |
| YBEY | -1.375230392 | -0.459673333 | 1.22038E-06 | 1.44923E-05 |
| PI4K2B | -1.37483962 | -0.459263333 | 1.36302E-05 | 0.000111511 |
| CCNC | -1.374709388 | -0.459126667 | 2.73562E-08 | 6.60698E-07 |
| AFF3 | -1.37266223 | -0.456976667 | 5.44005E-05 | 0.000361496 |
| PIGS | -1.372608316 | -0.45692 | 6.05121E-10 | 2.91152E-08 |
| SSFA2 | -1.37236414 | -0.456663333 | 4.42537E-07 | 6.22767E-06 |
| LRRFIP1 | -1.3722278 | -0.45652 | 2.9471E-05 | 0.000213874 |
| RAB23 | -1.371936144 | -0.456213333 | 1.17178E-05 | 9.78405E-05 |
| PDS5A | -1.371134407 | -0.45537 | 4.23927E-09 | 1.44691E-07 |
| SSR3 | -1.370643456 | -0.454853333 | 4.4669E-06 | 4.32383E-05 |
| HDHD2 | -1.370352136 | -0.454546667 | 1.1803E-08 | 3.31486E-07 |
| SLC30A9 | -1.369908942 | -0.45408 | 2.29579E-10 | 1.33048E-08 |
| TIPARP | -1.369874125 | -0.454043333 | 5.10952E-11 | 3.99406E-09 |
| C1orf174 | -1.369348823 | -0.45349 | 5.18532E-07 | 7.10687E-06 |
| ABHD2 | -1.368627651 | -0.45273 | 4.87777E-07 | 6.76027E-06 |
| IL1RAP | -1.368441094 | -0.452533333 | 1.51078E-08 | 4.07422E-07 |
| NAMPT | -1.366895861 | -0.450903333 | 3.60506E-14 | 1.34998E-11 |
| ABCB10 | -1.366542188 | -0.45053 | 3.93184E-08 | 8.81894E-07 |
| TIFA | -1.365667873 | -0.449606667 | 0.000128938 | 0.000750401 |
| MLPH | -1.365579526 | -0.449513333 | 4.42035E-06 | 4.28616E-05 |
| AMMECR1 | -1.364917103 | -0.448813333 | 8.54606E-09 | 2.55336E-07 |
| ERI2 | -1.364772044 | -0.44866 | 1.97937E-07 | 3.23874E-06 |
| RBMX | -1.364532415 | -0.448406667 | 4.10081E-10 | 2.14415E-08 |
| RNF144B | -1.364444141 | -0.448313333 | 2.882E-05 | 0.000209963 |
| NAP1L1 | -1.364270763 | -0.44813 | 7.94295E-07 | 1.01399E-05 |
| NSL1 | -1.364185658 | -0.44804 | 9.72448E-05 | 0.000589057 |
| EBF1 | -1.362601154 | -0.446363333 | 3.10423E-05 | 0.000223545 |
| RBM8A | -1.362248592 | -0.44599 | 1.5248E-11 | 1.57773E-09 |
| NMT2 | -1.362169908 | -0.445906667 | 4.16245E-09 | 1.42564E-07 |
| AMOTL1 | -1.36170419 | -0.445413333 | 9.19111E-08 | 1.75942E-06 |
| BRCC3 | -1.361697898 | -0.445406667 | 7.84821E-08 | 1.55302E-06 |
| MFSD6 | -1.36101849 | -0.444686667 | 0.000836444 | 0.003715754 |
| GMCL1P1 | -1.360911577 | -0.444573333 | 5.41499E-05 | 0.000360074 |
| GMCL1 | -1.360911577 | -0.444573333 | 5.41499E-05 | 0.000360074 |
| ENOSF1 | -1.360810961 | -0.444466667 | 2.18015E-12 | 3.4705E-10 |
| NUDT4P1 | -1.35980835 | -0.443403333 | 7.23062E-08 | 1.45274E-06 |
| NUDT4P2 | -1.35980835 | -0.443403333 | 7.23062E-08 | 1.45274E-06 |
| NUDT4 | -1.35980835 | -0.443403333 | 7.23062E-08 | 1.45274E-06 |
| AIDA | -1.359742373 | -0.443333333 | 1.17779E-08 | 3.31257E-07 |
| ZNF449 | -1.359714098 | -0.443303333 | 2.11408E-05 | 0.000161719 |
| TFAP2C | -1.359450229 | -0.443023333 | 4.44168E-10 | 2.28889E-08 |
| NEDD4L | -1.359425101 | -0.442996667 | 1.36483E-09 | 5.70078E-08 |
| SLC4A11 | -1.358998 | -0.442543333 | 8.66358E-10 | 3.91544E-08 |
| FAM136A | -1.358944622 | -0.442486667 | 2.62256E-10 | 1.46681E-08 |
| GPAM | -1.3585522 | -0.44207 | 3.9623E-06 | 3.90951E-05 |
| CAMK2N1 | -1.357049488 | -0.440473333 | 1.76437E-08 | 4.60104E-07 |
| GLUD1 | -1.356585521 | -0.43998 | 1.43586E-06 | 1.66834E-05 |
| PWAR6 | -1.35608098 | -0.439443333 | 0.000112992 | 0.00066969 |
| BEND6 | -1.355886735 | -0.439236667 | 5.73813E-05 | 0.000378109 |
| SACS | -1.355492064 | -0.438816667 | 1.42592E-11 | 1.49908E-09 |
| MAGI1 | -1.35513508 | -0.438436667 | 5.27258E-07 | 7.19585E-06 |
| MTCH2 | -1.354449559 | -0.437706667 | 1.26583E-07 | 2.27786E-06 |
| ETNK1 | -1.35352356 | -0.43672 | 3.24731E-07 | 4.84558E-06 |
| SLFN5 | -1.35348916 | -0.436683333 | 0.000102065 | 0.000613623 |
| C1orf50 | -1.353357823 | -0.436543333 | 1.35938E-08 | 3.7482E-07 |
| DRAM1 | -1.352982645 | -0.436143333 | 1.13224E-06 | 1.36101E-05 |
| TULP3 | -1.352860734 | -0.436013333 | 1.43306E-08 | 3.91022E-07 |
| LPXN | -1.352848231 | -0.436 | 2.22855E-10 | 1.30588E-08 |
| RMI2 | -1.352613821 | -0.43575 | 6.20986E-10 | 2.96649E-08 |
| INHBB | -1.351532933 | -0.434596667 | 5.99549E-07 | 8.01008E-06 |
| FAM24B | -1.351180113 | -0.43422 | 6.32056E-07 | 8.35074E-06 |
| SGMS2 | -1.350371786 | -0.433356667 | 3.00761E-05 | 0.000217542 |
| STAU1 | -1.348787745 | -0.431663333 | 9.74241E-09 | 2.8333E-07 |
| ALG13 | -1.348666212 | -0.431533333 | 4.76284E-07 | 6.63781E-06 |
| ETS1 | -1.348510417 | -0.431366667 | 2.55373E-05 | 0.000189153 |
| CHIC1 | -1.34850107 | -0.431356667 | 6.13541E-05 | 0.0003996 |
| USF2 | -1.348052484 | -0.430876667 | 1.63361E-05 | 0.000130288 |
| DOCK4 | -1.347893646 | -0.430706667 | 2.06835E-09 | 8.01237E-08 |
| ERGIC2 | -1.347744168 | -0.430546667 | 0.001213651 | 0.005103694 |
| DCBLD2 | -1.347744168 | -0.430546667 | 3.04778E-12 | 4.45486E-10 |
| MT1F | -1.347625843 | -0.43042 | 1.31479E-10 | 8.53072E-09 |
| SLC35E3 | -1.34730206 | -0.430073333 | 2.37117E-10 | 1.36333E-08 |
| RNF11 | -1.346732514 | -0.429463333 | 6.04746E-07 | 8.06295E-06 |
| INTS7 | -1.3464245 | -0.429133333 | 4.9428E-05 | 0.000333756 |
| SPATA33 | -1.346088564 | -0.428773333 | 1.05334E-05 | 8.94331E-05 |
| SP100 | -1.345892641 | -0.428563333 | 7.0304E-05 | 0.000448456 |
| RPL7L1 | -1.345870873 | -0.42854 | 9.9542E-10 | 4.3957E-08 |
| GRB2 | -1.34552264 | -0.428166667 | 9.09512E-11 | 6.3294E-09 |
| PRKCQ | -1.345485335 | -0.428126667 | 0.001024331 | 0.004413791 |
| SCAMP1 | -1.345339233 | -0.42797 | 7.45475E-07 | 9.6229E-06 |
| ABHD17C | -1.345317474 | -0.427946667 | 2.04416E-08 | 5.18878E-07 |
| MT1G | -1.344845089 | -0.42744 | 6.2854E-08 | 1.29526E-06 |
| FOXP1 | -1.34466488 | -0.427246667 | 1.52656E-09 | 6.23936E-08 |
| HNRNPD | -1.344130611 | -0.426673333 | 5.18702E-09 | 1.72545E-07 |
| CDCP1 | -1.344043657 | -0.42658 | 6.87008E-09 | 2.14095E-07 |
| ABCG1 | -1.343255117 | -0.425733333 | 1.1162E-08 | 3.1574E-07 |
| SHISA2 | -1.342817583 | -0.425263333 | 1.07353E-07 | 1.98822E-06 |
| LOC101927446 | -1.342708998 | -0.425146667 | 0.00497287 | 0.017040985 |
| GTPBP10 | -1.342708998 | -0.425146667 | 0.00497287 | 0.017040985 |
| TSPAN5 | -1.342588013 | -0.425016667 | 1.72966E-07 | 2.92761E-06 |
| DSN1 | -1.342513566 | -0.424936667 | 3.57929E-09 | 1.25584E-07 |
| TSPO | -1.342249933 | -0.424653333 | 5.98973E-13 | 1.33057E-10 |
| THOC1 | -1.341977051 | -0.42436 | 1.96191E-07 | 3.21687E-06 |
| ACSL1 | -1.341775525 | -0.424143333 | 3.66109E-07 | 5.32952E-06 |
| ZNF140 | -1.341403558 | -0.423743333 | 1.97989E-06 | 2.18243E-05 |
| PPM1E | -1.341254799 | -0.423583333 | 7.5901E-05 | 0.000479108 |
| ZNF569 | -1.340374984 | -0.422636667 | 0.001948181 | 0.007628017 |
| ZNF507 | -1.33970312 | -0.421913333 | 0.00269827 | 0.01009643 |
| C1QTNF9B-AS1 | -1.33964431 | -0.42185 | 2.2156E-05 | 0.000168241 |
| LAT | -1.337773 | -0.419833333 | 1.65107E-07 | 2.82499E-06 |
| GABPB1 | -1.33769882 | -0.419753333 | 3.64933E-06 | 3.64738E-05 |
| FLJ10038 | -1.33769882 | -0.419753333 | 3.64933E-06 | 3.64738E-05 |
| ZNF770 | -1.337491757 | -0.41953 | 1.28803E-05 | 0.000106194 |
| TBX3 | -1.337309444 | -0.419333333 | 5.57001E-09 | 1.81749E-07 |
| PTX3 | -1.337176587 | -0.41919 | 3.40677E-07 | 5.02629E-06 |
| FTSJ1 | -1.33716114 | -0.419173333 | 1.20274E-11 | 1.32466E-09 |
| LIMD1 | -1.33705919 | -0.419063333 | 8.25113E-07 | 1.04586E-05 |
| SAP30L | -1.336907825 | -0.4189 | 3.20778E-07 | 4.79714E-06 |
| GBAS | -1.33686767 | -0.418856667 | 1.78211E-08 | 4.63737E-07 |
| PCGF5 | -1.33628401 | -0.418226667 | 2.32357E-07 | 3.70931E-06 |
| PSMC3IP | -1.336253136 | -0.418193333 | 3.16124E-09 | 1.13617E-07 |
| SPIRE1 | -1.335524708 | -0.417406667 | 2.43446E-06 | 2.58914E-05 |
| HPCAL4 | -1.335524708 | -0.417406667 | 0.000135737 | 0.000785206 |
| VAV3 | -1.334920044 | -0.416753333 | 5.53597E-06 | 5.17273E-05 |
| FKBP9 | -1.334435894 | -0.41623 | 0.000992768 | 0.004298969 |
| DCTN4 | -1.334032055 | -0.415793333 | 3.34567E-07 | 4.96741E-06 |
| DOCK7 | -1.333622177 | -0.41535 | 0.000405463 | 0.001998044 |
| TUBA1C | -1.333566714 | -0.41529 | 9.14671E-11 | 6.34285E-09 |
| MRPS10 | -1.333542065 | -0.415263333 | 1.34286E-09 | 5.62898E-08 |
| SELT | -1.333329483 | -0.415033333 | 4.38348E-08 | 9.70462E-07 |
| MIPEP | -1.332614965 | -0.41426 | 3.28105E-09 | 1.16993E-07 |
| MOCS2 | -1.332451788 | -0.414083333 | 2.24291E-08 | 5.59574E-07 |
| USP24 | -1.332331728 | -0.413953333 | 2.01702E-10 | 1.20602E-08 |
| NUP58 | -1.332196288 | -0.413806667 | 1.825E-06 | 2.03624E-05 |
| ZNF468 | -1.331919294 | -0.413506667 | 9.58215E-05 | 0.000581959 |
| ERCC6L | -1.331651588 | -0.413216667 | 2.5811E-07 | 4.04006E-06 |
| CLDN12 | -1.331460842 | -0.41301 | 1.44834E-07 | 2.5457E-06 |
| TCP10 | -1.331297807 | -0.412833333 | 0.000339407 | 0.001718406 |
| CYP2U1 | -1.330977946 | -0.412486667 | 0.001252156 | 0.005239838 |
| USP37 | -1.330701206 | -0.412186667 | 1.78345E-07 | 2.99417E-06 |
| DAP | -1.330000388 | -0.411426667 | 5.92405E-12 | 7.49128E-10 |
| SH3KBP1 | -1.329745357 | -0.41115 | 9.59257E-07 | 1.18458E-05 |
| PEX1 | -1.329597892 | -0.41099 | 8.39293E-07 | 1.06008E-05 |
| SNRPA1 | -1.329447371 | -0.410826667 | 2.86331E-11 | 2.55869E-09 |
| YIPF6 | -1.328903796 | -0.410236667 | 5.46747E-08 | 1.1533E-06 |
| GPNMB | -1.328655115 | -0.409966667 | 5.3128E-06 | 4.99986E-05 |
| ATP9A | -1.32859372 | -0.4099 | 1.16899E-05 | 9.76906E-05 |
| PLAU | -1.328004467 | -0.40926 | 1.58904E-11 | 1.61864E-09 |
| CLIP4 | -1.327989126 | -0.409243333 | 7.51358E-08 | 1.49887E-06 |
| MCM9 | -1.327737549 | -0.40897 | 1.39549E-05 | 0.000113955 |
| SIM2 | -1.327550431 | -0.408766667 | 6.2058E-05 | 0.000403582 |
| OSBPL3 | -1.327372539 | -0.408573333 | 1.86108E-06 | 2.0677E-05 |
| TPM3 | -1.327246803 | -0.408436667 | 6.3952E-06 | 5.83553E-05 |
| PPP4R3A | -1.327118013 | -0.408296667 | 2.73E-11 | 2.45071E-09 |
| OTUD1 | -1.326468118 | -0.40759 | 3.85572E-06 | 3.81583E-05 |
| TMEM87B | -1.326241342 | -0.407343333 | 1.0383E-05 | 8.84043E-05 |
| BTBD11 | -1.325996224 | -0.407076667 | 4.15099E-09 | 1.4242E-07 |
| SLC30A5 | -1.325919633 | -0.406993333 | 1.11398E-07 | 2.04485E-06 |
| SULF2 | -1.325839984 | -0.406906667 | 6.1556E-10 | 2.94443E-08 |
| UHRF1BP1L | -1.325460184 | -0.406493333 | 8.91232E-06 | 7.75102E-05 |
| RARS2 | -1.325288697 | -0.406306667 | 1.31864E-10 | 8.54163E-09 |
| ASRGL1 | -1.325258077 | -0.406273333 | 2.02687E-06 | 2.22353E-05 |
| CCND1 | -1.325251953 | -0.406266667 | 1.33978E-07 | 2.38934E-06 |
| AEBP2 | -1.325242767 | -0.406256667 | 1.56132E-07 | 2.70201E-06 |
| TRIM9 | -1.32465194 | -0.405613333 | 0.0003637 | 0.001822624 |
| CYB5B | -1.324544824 | -0.405496667 | 5.29784E-06 | 4.98817E-05 |
| DOCK11 | -1.324147038 | -0.405063333 | 6.30977E-07 | 8.33929E-06 |
| SYNJ2 | -1.323798309 | -0.404683333 | 2.609E-11 | 2.39122E-09 |
| DSC2 | -1.323379344 | -0.404226667 | 0.000104094 | 0.000624102 |
| FOXQ1 | -1.323082785 | -0.403903333 | 0.000656377 | 0.003028761 |
| RCOR3 | -1.322951342 | -0.40376 | 0.000575945 | 0.002702983 |
| NRP1 | -1.322777123 | -0.40357 | 4.43149E-06 | 4.29379E-05 |
| TM4SF1 | -1.322697663 | -0.403483333 | 1.57957E-11 | 1.61318E-09 |
| SRPRB | -1.322679326 | -0.403463333 | 1.55484E-11 | 1.59205E-09 |
| METAP1 | -1.322480699 | -0.403246667 | 2.2597E-11 | 2.12558E-09 |
| RSRP1 | -1.321744509 | -0.402443333 | 0.000912701 | 0.003991381 |
| C1orf109 | -1.321323141 | -0.401983333 | 6.10532E-05 | 0.000397969 |
| SPNS2 | -1.32074932 | -0.401356667 | 8.10387E-05 | 0.000506932 |
| ZNF131 | -1.320706598 | -0.40131 | 7.11644E-08 | 1.43419E-06 |
| CUL4A | -1.320697444 | -0.4013 | 1.68607E-06 | 1.90776E-05 |
| LYRM2 | -1.32064557 | -0.401243333 | 7.6677E-05 | 0.000483461 |
| CTBS | -1.320142195 | -0.400693333 | 0.000136856 | 0.000790862 |
| FKTN | -1.319730485 | -0.400243333 | 1.05308E-07 | 1.95589E-06 |
| CMIP | -1.31967865 | -0.400186667 | 1.00461E-07 | 1.88852E-06 |
| TNFRSF11B | -1.319617669 | -0.40012 | 1.73127E-08 | 4.52604E-07 |
| FMNL2 | -1.319446938 | -0.399933333 | 3.09548E-06 | 3.17535E-05 |
| DCAF16 | -1.319004969 | -0.39945 | 9.48467E-05 | 0.000577198 |
| KLHL7 | -1.318937925 | -0.399376667 | 4.77176E-05 | 0.000323708 |
| APPL1 | -1.318819082 | -0.399246667 | 8.98223E-06 | 7.80174E-05 |
| RHOB | -1.318685016 | -0.3991 | 1.55705E-09 | 6.3508E-08 |
| NANP | -1.318554009 | -0.398956667 | 8.73139E-06 | 7.61049E-05 |
| SNED1 | -1.318362093 | -0.398746667 | 0.001254276 | 0.005248043 |
| MYEF2 | -1.318048386 | -0.398403333 | 2.82245E-06 | 2.93431E-05 |
| RGS17 | -1.317810871 | -0.398143333 | 1.70443E-09 | 6.8524E-08 |
| PICALM | -1.317521648 | -0.397826667 | 2.12983E-06 | 2.30951E-05 |
| SMS | -1.317320751 | -0.397606667 | 5.71868E-13 | 1.28487E-10 |
| DIMT1 | -1.317262923 | -0.397543333 | 0.002187611 | 0.008427855 |
| TPD52 | -1.316517471 | -0.396726667 | 0.0004043 | 0.001993311 |
| ECHDC1 | -1.316395804 | -0.396593333 | 8.55612E-10 | 3.87578E-08 |
| SOAT1 | -1.316331934 | -0.396523333 | 2.43216E-08 | 5.9657E-07 |
| NDRG1 | -1.316292397 | -0.39648 | 1.68086E-09 | 6.77845E-08 |
| METAP2 | -1.314957951 | -0.395016667 | 1.44237E-08 | 3.93019E-07 |
| FAM63B | -1.314909341 | -0.394963333 | 5.56819E-06 | 5.19748E-05 |
| COL13A1 | -1.314717955 | -0.394753333 | 1.1037E-10 | 7.36782E-09 |
| PHLDB2 | -1.314241132 | -0.39423 | 0.000523732 | 0.00249064 |
| TMEM136 | -1.314083241 | -0.394056667 | 4.80604E-05 | 0.000325558 |
| GOLT1B | -1.314058952 | -0.39403 | 2.69721E-07 | 4.18164E-06 |
| PNMA2 | -1.313312278 | -0.39321 | 2.65312E-07 | 4.13168E-06 |
| CREBZF | -1.313260695 | -0.393153333 | 0.001176338 | 0.004968037 |
| MAP1B | -1.312942134 | -0.392803333 | 1.39054E-07 | 2.46504E-06 |
| BVES | -1.312517507 | -0.392336667 | 8.3739E-05 | 0.00052023 |
| CWC27 | -1.312274925 | -0.39207 | 1.5408E-06 | 1.76936E-05 |
| AP1S2 | -1.31222035 | -0.39201 | 0.000670842 | 0.00308826 |
| WDR55 | -1.312211255 | -0.392 | 2.21966E-06 | 2.39306E-05 |
| GOLPH3 | -1.312111207 | -0.39189 | 1.23601E-07 | 2.23405E-06 |
| CTNNAL1 | -1.311811111 | -0.39156 | 1.09563E-10 | 7.32637E-09 |
| C2orf47 | -1.311780802 | -0.391526667 | 1.06981E-10 | 7.21506E-09 |
| PCF11 | -1.311608055 | -0.391336667 | 2.10948E-06 | 2.29293E-05 |
| AKAP2 | -1.311359581 | -0.391063333 | 5.10923E-06 | 4.83373E-05 |
| PALM2-AKAP2 | -1.311359581 | -0.391063333 | 5.10923E-06 | 4.83373E-05 |
| MSX1 | -1.311338372 | -0.39104 | 1.65579E-09 | 6.69105E-08 |
| NPAS2 | -1.311320193 | -0.39102 | 5.93227E-09 | 1.9166E-07 |
| CCDC82 | -1.311314133 | -0.391013333 | 0.000231523 | 0.001239382 |
| TLE1 | -1.311156594 | -0.39084 | 4.49387E-07 | 6.31502E-06 |
| GNA13 | -1.310229921 | -0.38982 | 5.42457E-10 | 2.68288E-08 |
| DENND3 | -1.310196621 | -0.389783333 | 5.67794E-07 | 7.66133E-06 |
| KAT2B | -1.310042243 | -0.389613333 | 5.60541E-06 | 5.22273E-05 |
| CRNKL1 | -1.309812224 | -0.38936 | 3.2662E-06 | 3.3236E-05 |
| S100A4 | -1.309509628 | -0.389026667 | 1.31926E-11 | 1.41341E-09 |
| ITGA3 | -1.309427939 | -0.388936667 | 3.54122E-05 | 0.000250292 |
| YKT6 | -1.309207102 | -0.388693333 | 2.09899E-06 | 2.28426E-05 |
| LOC339166 | -1.308865331 | -0.388316667 | 4.27713E-06 | 4.17111E-05 |
| WSCD1 | -1.308865331 | -0.388316667 | 4.27713E-06 | 4.17111E-05 |
| MBP | -1.308632495 | -0.38806 | 5.09874E-08 | 1.08955E-06 |
| KPNA1 | -1.30791308 | -0.387266667 | 1.59447E-05 | 0.00012745 |
| PPP2CA | -1.30767437 | -0.387003333 | 1.50736E-05 | 0.0001215 |
| MRS2 | -1.307236345 | -0.38652 | 1.96009E-05 | 0.000151949 |
| ABI2 | -1.30688603 | -0.386133333 | 2.27975E-09 | 8.65226E-08 |
| RAP1GAP2 | -1.306744119 | -0.385976667 | 0.00212726 | 0.00822355 |
| APBB2 | -1.306267169 | -0.38545 | 6.04493E-10 | 2.91152E-08 |
| FAM46C | -1.305935219 | -0.385083333 | 3.28413E-05 | 0.000234578 |
| TMEM161B | -1.305917115 | -0.385063333 | 6.21088E-05 | 0.000403779 |
| ANKLE2 | -1.305892976 | -0.385036667 | 5.27794E-08 | 1.12044E-06 |
| CAMK1D | -1.305675752 | -0.384796667 | 4.18551E-05 | 0.000288973 |
| HES6 | -1.305549055 | -0.384656667 | 7.73417E-07 | 9.91522E-06 |
| GRSF1 | -1.305416337 | -0.38451 | 1.49207E-06 | 1.72194E-05 |
| UPRT | -1.305033342 | -0.384086667 | 4.01262E-05 | 0.000278848 |
| CPT1A | -1.304870528 | -0.383906667 | 1.51528E-09 | 6.20618E-08 |
| HMGN2 | -1.304786114 | -0.383813333 | 4.24504E-14 | 1.53129E-11 |
| TMEM65 | -1.304267689 | -0.38324 | 3.24032E-08 | 7.56648E-07 |
| RAB10 | -1.303610912 | -0.382513333 | 2.30212E-05 | 0.000173704 |
| FAM84B | -1.302301358 | -0.381063333 | 0.002722364 | 0.010173996 |
| TMPO | -1.302093757 | -0.380833333 | 6.1035E-06 | 5.61234E-05 |
| NIP7 | -1.301964399 | -0.38069 | 5.64255E-11 | 4.33319E-09 |
| SLC16A10 | -1.30182603 | -0.380536667 | 0.001009822 | 0.004359374 |
| ALG10B | -1.301308782 | -0.379963333 | 1.8125E-05 | 0.000142275 |
| SLC43A3 | -1.301167476 | -0.379806667 | 6.93395E-08 | 1.40245E-06 |
| OPA1 | -1.300872889 | -0.37948 | 2.21592E-08 | 5.54954E-07 |
| UBE2D2 | -1.300866878 | -0.379473333 | 0.001350058 | 0.005584737 |
| ANGEL2 | -1.300866878 | -0.379473333 | 4.72976E-05 | 0.000321191 |
| SLC2A14 | -1.300815783 | -0.379416667 | 1.62002E-06 | 1.84844E-05 |
| SLC2A3 | -1.300815783 | -0.379416667 | 1.62002E-06 | 1.84844E-05 |
| CKB | -1.300542309 | -0.379113333 | 7.8645E-11 | 5.6531E-09 |
| NPM1 | -1.300497236 | -0.379063333 | 1.62169E-12 | 2.72492E-10 |
| CMBL | -1.300419114 | -0.378976667 | 4.89068E-08 | 1.0531E-06 |
| NPM3 | 1.300223829 | 0.37876 | 9.45147E-09 | 2.76711E-07 |
| CEACAM1 | 1.300380055 | 0.378933333 | 0.000296382 | 0.001528307 |
| ANKRD40 | 1.300653495 | 0.379236667 | 2.89308E-09 | 1.0523E-07 |
| CXXC5 | 1.30083081 | 0.379433333 | 3.61169E-10 | 1.93735E-08 |
| TNIK | 1.300875894 | 0.379483333 | 0.000326236 | 0.001661736 |
| SCAMP5 | 1.300981097 | 0.3796 | 1.74035E-05 | 0.000137573 |
| ZNF85 | 1.301005144 | 0.379626667 | 2.08424E-06 | 2.27424E-05 |
| KCTD20 | 1.30103821 | 0.379663333 | 8.63011E-07 | 1.08515E-05 |
| MIR20A | 1.301194534 | 0.379836667 | 0.000216036 | 0.001166641 |
| MIR19B1 | 1.301194534 | 0.379836667 | 0.000216036 | 0.001166641 |
| MIR17HG | 1.301194534 | 0.379836667 | 0.000216036 | 0.001166641 |
| MIR18A | 1.301194534 | 0.379836667 | 0.000216036 | 0.001166641 |
| MIR92A1 | 1.301194534 | 0.379836667 | 0.000216036 | 0.001166641 |
| MIR19A | 1.301194534 | 0.379836667 | 0.000216036 | 0.001166641 |
| OSBPL5 | 1.301320808 | 0.379976667 | 0.002271278 | 0.008701904 |
| B4GALT1 | 1.302030581 | 0.380763333 | 0.000532345 | 0.002524576 |
| NR2E1 | 1.302515012 | 0.3813 | 0.000367836 | 0.001840769 |
| ASPHD1 | 1.30259025 | 0.381383333 | 1.03701E-06 | 1.26629E-05 |
| EPSTI1 | 1.302671513 | 0.381473333 | 0.000907357 | 0.003971984 |
| KDELR3 | 1.302951455 | 0.381783333 | 2.12219E-07 | 3.43809E-06 |
| ZBTB4 | 1.302960486 | 0.381793333 | 4.23699E-10 | 2.20265E-08 |
| LGALS3BP | 1.303704287 | 0.382616667 | 7.39779E-08 | 1.48027E-06 |
| ERGIC1 | 1.303710311 | 0.382623333 | 5.83171E-08 | 1.22028E-06 |
| GREM1 | 1.303990477 | 0.382933333 | 3.22668E-11 | 2.79087E-09 |
| GNAI2 | 1.304153181 | 0.383113333 | 2.04671E-11 | 1.97242E-09 |
| CARD6 | 1.304195367 | 0.38316 | 4.78566E-05 | 0.000324482 |
| RTN2 | 1.304249608 | 0.38322 | 3.45631E-05 | 0.000245084 |
| PLD1 | 1.304457553 | 0.38345 | 1.04423E-07 | 1.94348E-06 |
| OK/SW-CL.58 | 1.304475637 | 0.38347 | 0.000202905 | 0.001104989 |
| LITAF | 1.304976053 | 0.384023333 | 7.02687E-09 | 2.17737E-07 |
| GXYLT2 | 1.305000175 | 0.38405 | 3.18115E-07 | 4.7704E-06 |
| CPNE2 | 1.305172052 | 0.38424 | 2.0599E-08 | 5.21846E-07 |
| FAM127C | 1.30518713 | 0.384256667 | 8.81792E-07 | 1.10594E-05 |
| HOXB8 | 1.305301729 | 0.384383333 | 7.22622E-06 | 6.47658E-05 |
| RALB | 1.305524923 | 0.38463 | 5.42833E-05 | 0.000360901 |
| ZNF136 | 1.305711954 | 0.384836667 | 0.002380114 | 0.009066431 |
| BEND7 | 1.306206809 | 0.385383333 | 0.000118252 | 0.000696648 |
| GANAB | 1.306360734 | 0.385553333 | 2.53117E-07 | 3.97774E-06 |
| EP400NL | 1.30642714 | 0.385626667 | 0.000213439 | 0.001154139 |
| CEP85 | 1.306490529 | 0.385696667 | 8.69363E-05 | 0.000536954 |
| TGM2 | 1.30655996 | 0.385773333 | 0.000958014 | 0.004164067 |
| CLPTM1 | 1.3067411 | 0.385973333 | 1.96899E-09 | 7.72643E-08 |
| RMDN2 | 1.306750158 | 0.385983333 | 9.54853E-06 | 8.22969E-05 |
| PDK2 | 1.306771292 | 0.386006667 | 1.1814E-05 | 9.8602E-05 |
| LSM14B | 1.306774312 | 0.38601 | 0.000385959 | 0.001917072 |
| SERPINA1 | 1.306795447 | 0.386033333 | 5.64568E-06 | 5.25651E-05 |
| SSPN | 1.306910187 | 0.38616 | 0.00019128 | 0.001049678 |
| IFITM1 | 1.307033996 | 0.386296667 | 1.60362E-10 | 9.9452E-09 |
| PYCARD | 1.307061176 | 0.386326667 | 5.19644E-07 | 7.11572E-06 |
| PFDN6 | 1.307100436 | 0.38637 | 2.58772E-09 | 9.61689E-08 |
| RAB2B | 1.307326959 | 0.38662 | 2.02776E-09 | 7.918E-08 |
| MDK | 1.307544457 | 0.38686 | 6.87167E-09 | 2.14095E-07 |
| C14orf159 | 1.307704584 | 0.387036667 | 9.83022E-06 | 8.43365E-05 |
| ZDHHC14 | 1.307804295 | 0.387146667 | 8.64097E-05 | 0.000534121 |
| DDIT3 | 1.308257624 | 0.387646667 | 5.37085E-10 | 2.67007E-08 |
| NR1H2 | 1.308423884 | 0.38783 | 7.89529E-09 | 2.38429E-07 |
| PNRC2 | 1.3089984 | 0.388463333 | 1.20763E-10 | 7.9138E-09 |
| CHAC1 | 1.309119382 | 0.388596667 | 1.05632E-08 | 3.01193E-07 |
| ZSWIM6 | 1.309960521 | 0.389523333 | 1.29843E-09 | 5.45439E-08 |
| SLC9B2 | 1.310199648 | 0.389786667 | 1.6159E-08 | 4.29584E-07 |
| BDH2 | 1.310199648 | 0.389786667 | 1.6159E-08 | 4.29584E-07 |
| ZNF93 | 1.31020873 | 0.389796667 | 6.20713E-06 | 5.69422E-05 |
| C11orf71 | 1.310635638 | 0.390266667 | 0.00024239 | 0.001289301 |
| ID2 | 1.310687119 | 0.390323333 | 2.33842E-07 | 3.73E-06 |
| HFE | 1.311105095 | 0.390783333 | 0.000222616 | 0.001197408 |
| GABARAP | 1.311302014 | 0.391 | 3.40233E-12 | 4.87046E-10 |
| ZNF680 | 1.311526235 | 0.391246667 | 5.0136E-05 | 0.000337898 |
| EDNRA | 1.3118687 | 0.391623333 | 0.000177169 | 0.000984748 |
| PELI1 | 1.312050576 | 0.391823333 | 0.009319716 | 0.029104358 |
| NEK6 | 1.312444728 | 0.392256667 | 3.16865E-06 | 3.2369E-05 |
| PFN2 | 1.312620618 | 0.39245 | 5.15542E-08 | 1.09868E-06 |
| SPR | 1.312875398 | 0.39273 | 1.72741E-07 | 2.92506E-06 |
| METTL7B | 1.312966403 | 0.39283 | 6.32733E-05 | 0.000410467 |
| SNN | 1.313200011 | 0.393086667 | 3.06174E-05 | 0.000220889 |
| DENND5B | 1.31349739 | 0.393413333 | 0.000633471 | 0.002936504 |
| FKBP5 | 1.313597543 | 0.393523333 | 3.1626E-07 | 4.74945E-06 |
| SH3BP5 | 1.313843405 | 0.393793333 | 8.28701E-08 | 1.62108E-06 |
| CDK5RAP2 | 1.314736181 | 0.394773333 | 7.42513E-08 | 1.48499E-06 |
| ZNF597 | 1.315039985 | 0.395106667 | 0.000280143 | 0.001456616 |
| SRP19 | 1.315310429 | 0.395403333 | 1.38732E-10 | 8.9131E-09 |
| RGS16 | 1.315483664 | 0.395593333 | 0.001408754 | 0.005783129 |
| SLC19A1 | 1.31574508 | 0.39588 | 5.87249E-06 | 5.43776E-05 |
| ICAM1 | 1.315824123 | 0.395966667 | 4.49063E-06 | 4.34466E-05 |
| RAB13 | 1.315857565 | 0.396003333 | 2.17672E-12 | 3.4705E-10 |
| TRIM34 | 1.315951817 | 0.396106667 | 0.002590028 | 0.009747997 |
| TRIM6-TRIM34 | 1.315951817 | 0.396106667 | 0.002590028 | 0.009747997 |
| NR4A2 | 1.315957898 | 0.396113333 | 0.000175926 | 0.000978667 |
| CKMT1A | 1.316526596 | 0.396736667 | 2.4179E-06 | 2.57571E-05 |
| CKMT1B | 1.316526596 | 0.396736667 | 2.4179E-06 | 2.57571E-05 |
| FILIP1L | 1.316541805 | 0.396753333 | 0.001648801 | 0.006613875 |
| PAIP1 | 1.31676388 | 0.396996667 | 5.67391E-07 | 7.66114E-06 |
| DZIP1 | 1.317083367 | 0.397346667 | 1.0401E-07 | 1.93819E-06 |
| TMEM50B | 1.317098583 | 0.397363333 | 4.06802E-10 | 2.13267E-08 |
| SPP1 | 1.317339013 | 0.397626667 | 0.000310641 | 0.001589964 |
| TMEM138 | 1.317698219 | 0.39802 | 4.62307E-09 | 1.56432E-07 |
| RBPMS2 | 1.318106249 | 0.398466667 | 1.79752E-07 | 3.01007E-06 |
| RASL12 | 1.318203708 | 0.398573333 | 6.11589E-06 | 5.6211E-05 |
| TRPV4 | 1.319099447 | 0.399553333 | 0.006221682 | 0.020657854 |
| FAM89A | 1.319111638 | 0.399566667 | 2.25278E-06 | 2.42213E-05 |
| PPME1 | 1.319190883 | 0.399653333 | 1.25456E-06 | 1.48054E-05 |
| IPO7 | 1.319248796 | 0.399716667 | 9.55234E-07 | 1.18035E-05 |
| UCP2 | 1.319288422 | 0.39976 | 2.12983E-07 | 3.44621E-06 |
| SLC45A4 | 1.319440841 | 0.399926667 | 4.76408E-07 | 6.63781E-06 |
| MTHFR | 1.319605473 | 0.400106667 | 2.56015E-05 | 0.000189429 |
| ABCA1 | 1.320050693 | 0.400593333 | 1.04311E-08 | 2.98602E-07 |
| GBP2 | 1.320172697 | 0.400726667 | 0.000817361 | 0.003643332 |
| TMEM45A | 1.320550982 | 0.40114 | 3.86818E-05 | 0.00027058 |
| SLC39A10 | 1.320651673 | 0.40125 | 7.36482E-08 | 1.47593E-06 |
| ADM | 1.320764578 | 0.401373333 | 8.18856E-08 | 1.60742E-06 |
| TMEM234 | 1.320810353 | 0.401423333 | 0.000272836 | 0.001425221 |
| PDIA4 | 1.321301771 | 0.40196 | 3.15467E-13 | 8.13769E-11 |
| RRBP1 | 1.32158877 | 0.402273333 | 3.91885E-07 | 5.64415E-06 |
| G0S2 | 1.322056041 | 0.402783333 | 8.50159E-06 | 7.45315E-05 |
| SOD2 | 1.322373758 | 0.40313 | 0.000256473 | 0.001351996 |
| LOC100129518 | 1.322373758 | 0.40313 | 0.000256473 | 0.001351996 |
| TPM4 | 1.322532645 | 0.403303333 | 7.32782E-13 | 1.54076E-10 |
| IFI6 | 1.322712943 | 0.4035 | 1.42709E-05 | 0.000115981 |
| ZBED1 | 1.323412979 | 0.404263333 | 3.29352E-08 | 7.65355E-07 |
| TTC39C | 1.323810544 | 0.404696667 | 1.84848E-05 | 0.000144577 |
| C4orf19 | 1.323868659 | 0.40476 | 0.001658088 | 0.006644505 |
| NPY | 1.324572367 | 0.405526667 | 0.000109143 | 0.00065041 |
| C11orf86 | 1.324581549 | 0.405536667 | 0.000740465 | 0.003351485 |
| STAT1 | 1.324734579 | 0.405703333 | 2.80122E-07 | 4.31081E-06 |
| CATSPER2 | 1.325187653 | 0.406196667 | 6.67748E-05 | 0.00043006 |
| ZNF708 | 1.325432622 | 0.406463333 | 3.20784E-05 | 0.000229743 |
| ADRB1 | 1.326189251 | 0.407286667 | 0.000818225 | 0.003646355 |
| WRB | 1.327801972 | 0.40904 | 8.52403E-11 | 6.01717E-09 |
| TSPAN31 | 1.329164807 | 0.41052 | 0.000164958 | 0.000926038 |
| BNC1 | 1.329435085 | 0.410813333 | 0.000158521 | 0.000896044 |
| ZNF222 | 1.329683912 | 0.411083333 | 0.001359767 | 0.005614504 |
| ALG9 | 1.330049556 | 0.41148 | 0.000292602 | 0.001511607 |
| TCF7L1 | 1.330135605 | 0.411573333 | 8.15057E-05 | 0.000508707 |
| PRTG | 1.330203218 | 0.411646667 | 9.11079E-07 | 1.13635E-05 |
| TEAD2 | 1.330584377 | 0.41206 | 2.65738E-07 | 4.13168E-06 |
| C19orf66 | 1.331033301 | 0.412546667 | 5.03391E-06 | 4.77627E-05 |
| SNORA12 | 1.331350099 | 0.41289 | 6.67597E-06 | 6.04682E-05 |
| TCF4 | 1.331460842 | 0.41301 | 2.11481E-05 | 0.000161743 |
| ADAMTS6 | 1.331873134 | 0.413456667 | 8.28902E-05 | 0.000516425 |
| SUGCT | 1.33190083 | 0.413486667 | 6.56568E-05 | 0.000423971 |
| SPECC1 | 1.331922371 | 0.41351 | 0.000440946 | 0.002148928 |
| CCDC3 | 1.332190132 | 0.4138 | 4.53357E-06 | 4.37488E-05 |
| NPW | 1.332254771 | 0.41387 | 5.42297E-07 | 7.36786E-06 |
| LRRC8B | 1.332553387 | 0.414193333 | 4.01383E-08 | 8.97722E-07 |
| BIRC3 | 1.332735051 | 0.41439 | 0.00039322 | 0.001947232 |
| RBM15B | 1.33273813 | 0.414393333 | 8.50928E-09 | 2.54818E-07 |
| LRIG2 | 1.332830512 | 0.414493333 | 4.95074E-06 | 4.70416E-05 |
| LCP1 | 1.333012214 | 0.41469 | 4.56304E-05 | 0.000311483 |
| ZNF493 | 1.333625258 | 0.415353333 | 0.000200364 | 0.001092666 |
| HOXC8 | 1.334226252 | 0.416003333 | 6.77985E-08 | 1.37837E-06 |
| ZNF652 | 1.334445143 | 0.41624 | 0.000107187 | 0.000640403 |
| ARHGEF39 | 1.334617815 | 0.416426667 | 4.5341E-06 | 4.37488E-05 |
| HSD17B7P2 | 1.334623982 | 0.416433333 | 0.000377154 | 0.001879272 |
| UNC119 | 1.334660986 | 0.416473333 | 1.38799E-06 | 1.61893E-05 |
| CTSS | 1.334747333 | 0.416566667 | 0.00013275 | 0.000769626 |
| TAF15 | 1.334892285 | 0.416723333 | 3.85033E-07 | 5.56015E-06 |
| ZNF346 | 1.334910791 | 0.416743333 | 4.15678E-10 | 2.17052E-08 |
| LOC102723841 | 1.335179152 | 0.417033333 | 8.47069E-06 | 7.4327E-05 |
| LOC101929031 | 1.335179152 | 0.417033333 | 8.47069E-06 | 7.4327E-05 |
| CHEK2 | 1.335179152 | 0.417033333 | 8.47069E-06 | 7.4327E-05 |
| LOC102723890 | 1.335179152 | 0.417033333 | 8.47069E-06 | 7.4327E-05 |
| EFHD1 | 1.335210002 | 0.417066667 | 0.000284433 | 0.0014758 |
| ATF7IP | 1.335771588 | 0.417673333 | 5.26775E-09 | 1.7464E-07 |
| KBTBD8 | 1.335793192 | 0.417696667 | 0.000242298 | 0.001288987 |
| CCNO | 1.336138907 | 0.41807 | 3.09292E-06 | 3.17355E-05 |
| FCMR | 1.337040655 | 0.419043333 | 6.87552E-05 | 0.000440506 |
| CCDC59 | 1.337068458 | 0.419073333 | 2.07937E-05 | 0.000159343 |
| PDZD2 | 1.337399052 | 0.41943 | 0.000605133 | 0.002821441 |
| MTL5 | 1.337921372 | 0.419993333 | 0.00044054 | 0.002147749 |
| TFPI2 | 1.338171787 | 0.420263333 | 0.000410533 | 0.00201974 |
| ARID3A | 1.339331727 | 0.421513333 | 6.4565E-08 | 1.32427E-06 |
| DUSP6 | 1.339916718 | 0.422143333 | 1.33105E-12 | 2.33434E-10 |
| LATS2 | 1.340090098 | 0.42233 | 2.23756E-07 | 3.58804E-06 |
| ISG15 | 1.340390469 | 0.422653333 | 2.62344E-09 | 9.7404E-08 |
| NMB | 1.342194112 | 0.424593333 | 3.50872E-07 | 5.16315E-06 |
| BANF1 | 1.342597319 | 0.425026667 | 1.42455E-05 | 0.000115823 |
| CXADR | 1.343099947 | 0.425566667 | 1.40947E-07 | 2.48738E-06 |
| CTSO | 1.34361518 | 0.42612 | 3.29704E-05 | 0.000235232 |
| LAMTOR4 | 1.343969129 | 0.4265 | 5.98704E-12 | 7.50219E-10 |
| TAGLN | 1.344379082 | 0.42694 | 6.99609E-05 | 0.000446702 |
| TRABD2A | 1.344826445 | 0.42742 | 5.47561E-06 | 5.1252E-05 |
| ALDH5A1 | 1.345292608 | 0.42792 | 0.000534044 | 0.00253233 |
| SEMA6D | 1.345587927 | 0.428236667 | 0.000696554 | 0.003185 |
| RPL28 | 1.345737166 | 0.428396667 | 1.57267E-08 | 4.20939E-07 |
| PAPLN | 1.346688952 | 0.429416667 | 3.57588E-08 | 8.17917E-07 |
| CLCF1 | 1.346965905 | 0.429713333 | 1.01344E-07 | 1.89638E-06 |
| SPARC | 1.347915446 | 0.43073 | 8.48345E-11 | 5.99929E-09 |
| C9orf3 | 1.348769047 | 0.431643333 | 5.83531E-06 | 5.40746E-05 |
| RPSA | 1.349027726 | 0.43192 | 3.16856E-06 | 3.2369E-05 |
| SNORA6 | 1.349027726 | 0.43192 | 3.16856E-06 | 3.2369E-05 |
| UTP14C | 1.34944234 | 0.432363333 | 5.39572E-07 | 7.33844E-06 |
| ALG11 | 1.34944234 | 0.432363333 | 5.39572E-07 | 7.33844E-06 |
| HSPB1 | 1.349822774 | 0.43277 | 6.77243E-13 | 1.47119E-10 |
| UGCG | 1.34991634 | 0.43287 | 4.81025E-10 | 2.44675E-08 |
| HTRA1 | 1.350003674 | 0.432963333 | 4.35887E-06 | 4.23909E-05 |
| SLC39A11 | 1.351002178 | 0.43403 | 5.59135E-08 | 1.17565E-06 |
| NRIP3 | 1.35170157 | 0.434776667 | 5.86732E-06 | 5.43456E-05 |
| SDF2L1 | 1.351707816 | 0.434783333 | 1.25371E-13 | 3.70637E-11 |
| HCCS | 1.352010791 | 0.435106667 | 1.87111E-07 | 3.10513E-06 |
| ANGEL1 | 1.352220103 | 0.43533 | 2.03343E-07 | 3.31477E-06 |
| CDIP1 | 1.352476319 | 0.435603333 | 0.000549142 | 0.002594844 |
| MXD4 | 1.352673201 | 0.435813333 | 5.28804E-07 | 7.21341E-06 |
| AKT1 | 1.356623134 | 0.44002 | 3.97843E-09 | 1.36977E-07 |
| GALNT3 | 1.35664821 | 0.440046667 | 2.93105E-06 | 3.03119E-05 |
| ZNF804A | 1.357394432 | 0.44084 | 1.86209E-09 | 7.418E-08 |
| VPS36 | 1.357428931 | 0.440876667 | 2.228E-10 | 1.30588E-08 |
| CC2D2A | 1.357435203 | 0.440883333 | 2.97139E-08 | 7.05083E-07 |
| ZNF134 | 1.358276003 | 0.441776667 | 3.46449E-06 | 3.49017E-05 |
| FAM50B | 1.358395263 | 0.441903333 | 4.60687E-10 | 2.36164E-08 |
| TMEM54 | 1.358445481 | 0.441956667 | 3.70596E-06 | 3.68991E-05 |
| LMCD1 | 1.359748656 | 0.44334 | 1.02189E-06 | 1.25171E-05 |
| SCARNA20 | 1.360424287 | 0.444056667 | 7.13808E-07 | 9.2873E-06 |
| FBXL17 | 1.361049937 | 0.44472 | 0.001373345 | 0.005659092 |
| ATM | 1.361053081 | 0.444723333 | 6.59251E-07 | 8.66057E-06 |
| ZNRF1 | 1.361263793 | 0.444946667 | 6.49964E-07 | 8.56654E-06 |
| BTN3A3 | 1.361896122 | 0.445616667 | 2.82557E-05 | 0.00020635 |
| MATN3 | 1.362796361 | 0.44657 | 3.02111E-05 | 0.000218479 |
| TLCD1 | 1.363297101 | 0.4471 | 2.90997E-08 | 6.92597E-07 |
| MALAT1 | 1.363460904 | 0.447273333 | 2.36329E-09 | 8.91769E-08 |
| CEP126 | 1.364091103 | 0.44794 | 1.78768E-05 | 0.000140579 |
| TRIM16 | 1.364601777 | 0.44848 | 1.61599E-09 | 6.55718E-08 |
| TRIM16L | 1.364601777 | 0.44848 | 1.61599E-09 | 6.55718E-08 |
| AGPAT4 | 1.365065332 | 0.44897 | 1.2268E-06 | 1.45422E-05 |
| CDC14B | 1.366845331 | 0.45085 | 1.10983E-05 | 9.35022E-05 |
| SPATA2 | 1.367685639 | 0.451736667 | 2.54536E-05 | 0.000188654 |
| IGFBP5 | 1.368036447 | 0.452106667 | 6.99E-05 | 0.000446561 |
| TPST1 | 1.368286176 | 0.45237 | 4.55973E-10 | 2.34358E-08 |
| HNMT | 1.368912279 | 0.45303 | 6.46597E-06 | 5.89327E-05 |
| AIFM2 | 1.36894707 | 0.453066667 | 3.77169E-08 | 8.52784E-07 |
| BLCAP | 1.369984908 | 0.45416 | 6.39082E-12 | 7.97717E-10 |
| RARRES1 | 1.370355302 | 0.45455 | 0.000677864 | 0.003115479 |
| KRTAP2-3 | 1.370437626 | 0.454636667 | 8.58754E-06 | 7.50881E-05 |
| TFAP2A | 1.371064713 | 0.455296667 | 2.95537E-05 | 0.000214356 |
| TENM3 | 1.371150247 | 0.455386667 | 6.37887E-06 | 5.82468E-05 |
| GALNT2 | 1.372513177 | 0.45682 | 9.37007E-09 | 2.74942E-07 |
| TCFL5 | 1.372716147 | 0.457033333 | 1.03116E-07 | 1.92518E-06 |
| MAML2 | 1.373290336 | 0.457636667 | 9.37238E-07 | 1.16242E-05 |
| AKIP1 | 1.373496594 | 0.457853333 | 3.39004E-07 | 5.01102E-06 |
| NUAK2 | 1.373496594 | 0.457853333 | 3.39004E-07 | 5.01102E-06 |
| C3 | 1.373928252 | 0.458306667 | 3.34872E-05 | 0.000238487 |
| RTFDC1 | 1.374458487 | 0.458863333 | 3.55973E-05 | 0.000251433 |
| IL1R1 | 1.374712564 | 0.45913 | 0.000334834 | 0.001699851 |
| PDCD1LG2 | 1.374871386 | 0.459296667 | 0.000223564 | 0.001202012 |
| RIT1 | 1.376651449 | 0.461163333 | 8.39743E-07 | 1.0603E-05 |
| CMTM6 | 1.376867756 | 0.46139 | 2.23777E-11 | 2.11506E-09 |
| RCC1 | 1.376975923 | 0.461503333 | 6.29765E-09 | 2.00825E-07 |
| NR4A1 | 1.377068189 | 0.4616 | 1.93999E-05 | 0.000150658 |
| TMEM220 | 1.377119097 | 0.461653333 | 3.52761E-08 | 8.08759E-07 |
| EXTL2 | 1.377233648 | 0.461773333 | 1.4561E-10 | 9.24915E-09 |
| PTGER4 | 1.377322749 | 0.461866667 | 8.49197E-05 | 0.000526401 |
| GABPB2 | 1.377816092 | 0.462383333 | 2.17431E-06 | 2.35061E-05 |
| ZNF609 | 1.378988093 | 0.46361 | 0.000853762 | 0.003779451 |
| CYS1 | 1.379010396 | 0.463633333 | 6.20205E-05 | 0.000403405 |
| KIAA1217 | 1.380237625 | 0.464916667 | 4.10076E-05 | 0.000284069 |
| CPA4 | 1.380741584 | 0.465443333 | 5.33879E-07 | 7.27612E-06 |
| CYP1B1 | 1.381028731 | 0.465743333 | 9.81009E-07 | 1.20727E-05 |
| CREBL2 | 1.381197856 | 0.46592 | 1.07317E-08 | 3.05325E-07 |
| CREBRF | 1.381555322 | 0.466293333 | 2.60363E-06 | 2.73722E-05 |
| COL5A2 | 1.38283913 | 0.467633333 | 9.1677E-10 | 4.1102E-08 |
| RYR1 | 1.382880666 | 0.467676667 | 5.00621E-06 | 4.75342E-05 |
| KRT8 | 1.383053214 | 0.467856667 | 4.23819E-13 | 1.00995E-10 |
| LYRM9 | 1.383161867 | 0.46797 | 1.21707E-06 | 1.44618E-05 |
| BTN3A2 | 1.383727636 | 0.46856 | 0.000310308 | 0.001588674 |
| TBC1D22B | 1.384220075 | 0.469073333 | 4.63654E-05 | 0.0003158 |
| GABRE | 1.384962263 | 0.469846667 | 5.11415E-07 | 7.02598E-06 |
| SPOCD1 | 1.386672087 | 0.471626667 | 2.78804E-07 | 4.29221E-06 |
| DNAJC27 | 1.386960467 | 0.471926667 | 8.54264E-06 | 7.47914E-05 |
| KLHL15 | 1.387082246 | 0.472053333 | 1.14153E-07 | 2.09054E-06 |
| IFNLR1 | 1.387117499 | 0.47209 | 1.75159E-05 | 0.000138378 |
| GJA1 | 1.387181599 | 0.472156667 | 2.65855E-07 | 4.13168E-06 |
| ATF5 | 1.387325835 | 0.472306667 | 9.39868E-08 | 1.79131E-06 |
| HACD2 | 1.387809935 | 0.47281 | 1.91656E-06 | 2.12094E-05 |
| HERPUD1 | 1.38803441 | 0.473043333 | 2.72054E-15 | 1.94489E-12 |
| PLLP | 1.388307035 | 0.473326667 | 1.87043E-05 | 0.00014592 |
| AKIRIN1 | 1.390380737 | 0.47548 | 8.76284E-08 | 1.69448E-06 |
| HHLA3 | 1.390628119 | 0.475736667 | 4.71698E-07 | 6.58852E-06 |
| ODC1 | 1.392341721 | 0.477513333 | 2.71655E-10 | 1.50228E-08 |
| LONRF1 | 1.3929209 | 0.478113333 | 1.59378E-08 | 4.25142E-07 |
| IER3IP1 | 1.393136544 | 0.478336667 | 4.36596E-09 | 1.48628E-07 |
| ZNF629 | 1.393761137 | 0.478983333 | 1.34871E-06 | 1.58063E-05 |
| MRPL33 | 1.393918939 | 0.479146667 | 1.46446E-10 | 9.28725E-09 |
| IL2RB | 1.394711441 | 0.479966667 | 1.54726E-10 | 9.71833E-09 |
| PON2 | 1.398202513 | 0.483573333 | 7.18683E-13 | 1.52745E-10 |
| LXN | 1.398428669 | 0.483806667 | 3.01505E-07 | 4.5754E-06 |
| BID | 1.399498558 | 0.48491 | 4.18376E-08 | 9.29912E-07 |
| PSMB8 | 1.399540594 | 0.484953333 | 3.06129E-11 | 2.69277E-09 |
| PRSS16 | 1.400763438 | 0.486213333 | 0.000114342 | 0.000676674 |
| AKAP7 | 1.400808749 | 0.48626 | 2.91526E-05 | 0.000211916 |
| ZC3H12A | 1.400850825 | 0.486303333 | 2.5741E-07 | 4.03365E-06 |
| EVI2B | 1.401482115 | 0.486953333 | 9.89932E-08 | 1.86949E-06 |
| PLA2G15 | 1.402794164 | 0.488303333 | 2.37619E-05 | 0.000178301 |
| PPP1R8 | 1.402995129 | 0.48851 | 1.17365E-07 | 2.14139E-06 |
| FAM3C | 1.403598197 | 0.48913 | 2.00366E-11 | 1.94496E-09 |
| BTF3L4 | 1.403809008 | 0.489346667 | 4.39837E-09 | 1.49472E-07 |
| RNASEL | 1.403828469 | 0.489366667 | 2.27779E-05 | 0.000172132 |
| GBP3 | 1.404672041 | 0.490233333 | 2.72539E-06 | 2.84621E-05 |
| ZNF256 | 1.405236868 | 0.490813333 | 2.40352E-07 | 3.81633E-06 |
| RCSD1 | 1.405347263 | 0.490926667 | 4.26042E-05 | 0.000293424 |
| KLRC2 | 1.406432194 | 0.49204 | 7.35308E-06 | 6.57737E-05 |
| KLRC1 | 1.406432194 | 0.49204 | 7.35308E-06 | 6.57737E-05 |
| UBE2L6 | 1.407108262 | 0.492733333 | 2.45936E-05 | 0.000183456 |
| P2RX4 | 1.408025374 | 0.493673333 | 1.85102E-06 | 2.05826E-05 |
| FST | 1.408145749 | 0.493796667 | 9.34464E-05 | 0.000570181 |
| HPSE | 1.408471137 | 0.49413 | 2.87265E-05 | 0.00020936 |
| IL1A | 1.410744443 | 0.496456667 | 3.31393E-06 | 3.3652E-05 |
| RCAN1 | 1.412003176 | 0.497743333 | 3.78815E-11 | 3.16907E-09 |
| RNF44 | 1.412094526 | 0.497836667 | 1.06366E-12 | 2.03019E-10 |
| AFF4 | 1.412358824 | 0.498106667 | 0.002192545 | 0.008442728 |
| ABCC5 | 1.413178136 | 0.498943333 | 1.83113E-08 | 4.74609E-07 |
| CROT | 1.413204257 | 0.49897 | 8.60045E-06 | 7.51803E-05 |
| NUBPL | 1.413308747 | 0.499076667 | 1.77157E-07 | 2.97931E-06 |
| MGST2 | 1.413458965 | 0.49923 | 6.7495E-10 | 3.17065E-08 |
| MCM6 | 1.413560208 | 0.499333333 | 1.56003E-08 | 4.18123E-07 |
| LDLR | 1.414458648 | 0.50025 | 9.30875E-09 | 2.73755E-07 |
| TESK2 | 1.414788764 | 0.500586667 | 4.65099E-06 | 4.46465E-05 |
| EGR4 | 1.41489991 | 0.5007 | 2.50115E-05 | 0.000186014 |
| SGK3 | 1.415014333 | 0.500816667 | 4.1096E-06 | 4.02855E-05 |
| C8orf44-SGK3 | 1.415014333 | 0.500816667 | 4.1096E-06 | 4.02855E-05 |
| GPC2 | 1.416692518 | 0.502526667 | 1.03958E-05 | 8.84742E-05 |
| GRAMD1B | 1.416859464 | 0.502696667 | 1.31632E-07 | 2.35255E-06 |
| TMEM144 | 1.417177043 | 0.50302 | 0.000577455 | 0.002709099 |
| YOD1 | 1.418667666 | 0.504536667 | 1.10778E-09 | 4.80368E-08 |
| NID2 | 1.4192414 | 0.50512 | 4.13598E-07 | 5.89854E-06 |
| ABL2 | 1.420392847 | 0.50629 | 6.45814E-11 | 4.81836E-09 |
| ST8SIA4 | 1.421023093 | 0.50693 | 0.00024155 | 0.001285703 |
| MEST | 1.421256224 | 0.507166667 | 2.62611E-11 | 2.40009E-09 |
| CA13 | 1.422129983 | 0.508053333 | 8.0406E-06 | 7.10606E-05 |
| USF3 | 1.423086477 | 0.509023333 | 1.95974E-09 | 7.70551E-08 |
| CD24 | 1.423648841 | 0.509593333 | 1.49166E-10 | 9.39914E-09 |
| PDZK1 | 1.424826907 | 0.510786667 | 2.52313E-05 | 0.000187325 |
| PDZK1P1 | 1.424826907 | 0.510786667 | 2.52313E-05 | 0.000187325 |
| ITGB1BP1 | 1.425093588 | 0.511056667 | 5.08166E-05 | 0.000341665 |
| TRAPPC1 | 1.425195665 | 0.51116 | 7.67939E-10 | 3.51919E-08 |
| GNG7 | 1.426259669 | 0.512236667 | 1.32461E-05 | 0.000108868 |
| LOC101929097 | 1.426259669 | 0.512236667 | 1.32461E-05 | 0.000108868 |
| EGR2 | 1.429403606 | 0.515413333 | 2.18481E-10 | 1.286E-08 |
| CREG1 | 1.431370019 | 0.517396667 | 2.86936E-09 | 1.04657E-07 |
| TP53I3 | 1.431714005 | 0.517743333 | 3.37695E-08 | 7.80589E-07 |
| PCDHB2 | 1.431975358 | 0.518006667 | 0.000169682 | 0.000949031 |
| COL1A1 | 1.432355894 | 0.51839 | 4.78381E-07 | 6.65822E-06 |
| SLC9A6 | 1.432428704 | 0.518463333 | 2.67345E-10 | 1.48672E-08 |
| TMEM41B | 1.432597504 | 0.518633333 | 1.1408E-07 | 2.09017E-06 |
| F3 | 1.433084157 | 0.519123333 | 2.22659E-05 | 0.000168981 |
| HMGA2 | 1.433484859 | 0.519526667 | 6.38442E-14 | 2.19564E-11 |
| PIR | 1.433564351 | 0.519606667 | 2.63408E-05 | 0.000194205 |
| IGF1R | 1.434220324 | 0.520266667 | 7.45105E-09 | 2.28881E-07 |
| TAPBPL | 1.434618029 | 0.520666667 | 2.63823E-07 | 4.11148E-06 |
| KANK1 | 1.435257903 | 0.52131 | 3.51112E-07 | 5.16475E-06 |
| NFKBIZ | 1.435334177 | 0.521386667 | 1.59886E-12 | 2.71298E-10 |
| MICA | 1.436176771 | 0.522233333 | 2.57906E-10 | 1.45391E-08 |
| STOX2 | 1.436674598 | 0.522733333 | 2.994E-07 | 4.55221E-06 |
| MR1 | 1.437331994 | 0.523393333 | 3.59178E-07 | 5.2523E-06 |
| FOXRED2 | 1.4379465 | 0.52401 | 9.00372E-11 | 6.2769E-09 |
| RILPL2 | 1.438308683 | 0.524373333 | 1.58843E-05 | 0.000127045 |
| OSMR | 1.438621098 | 0.524686667 | 3.47753E-08 | 7.99609E-07 |
| MICB | 1.439109797 | 0.525176667 | 7.97949E-07 | 1.01766E-05 |
| ZBTB5 | 1.439232829 | 0.5253 | 6.75576E-11 | 4.9783E-09 |
| NNMT | 1.439688472 | 0.525756667 | 9.98016E-13 | 1.97191E-10 |
| ADPGK | 1.44000784 | 0.526076667 | 2.00905E-10 | 1.20455E-08 |
| SP110 | 1.440583548 | 0.526653333 | 7.30369E-06 | 6.53857E-05 |
| KIAA1524 | 1.44092642 | 0.526996667 | 2.73533E-07 | 4.22596E-06 |
| MOB1A | 1.442275396 | 0.528346667 | 1.82159E-10 | 1.11911E-08 |
| SFSWAP | 1.443455535 | 0.529526667 | 1.85328E-07 | 3.08119E-06 |
| GDPD1 | 1.446720941 | 0.532786667 | 7.44601E-05 | 0.000471449 |
| INHBA | 1.449019155 | 0.535076667 | 1.12328E-09 | 4.8473E-08 |
| TBL1XR1 | 1.450506406 | 0.536556667 | 9.619E-14 | 2.97803E-11 |
| MAPK13 | 1.450526514 | 0.536576667 | 5.21403E-05 | 0.000348776 |
| PPP1R1C | 1.453008692 | 0.539043333 | 1.8946E-05 | 0.000147571 |
| SLC7A2 | 1.453260501 | 0.539293333 | 7.36175E-10 | 3.39738E-08 |
| RAB9B | 1.453431756 | 0.539463333 | 0.000108441 | 0.000646639 |
| SH3RF2 | 1.454432827 | 0.540456667 | 1.1123E-05 | 9.36503E-05 |
| SLC25A1 | 1.456817341 | 0.54282 | 3.89967E-07 | 5.62065E-06 |
| LMNB1 | 1.458858543 | 0.54484 | 1.42721E-07 | 2.51417E-06 |
| LOC100287934 | 1.458969779 | 0.54495 | 0.000622168 | 0.00288956 |
| DCAF5 | 1.459097881 | 0.545076667 | 2.47235E-05 | 0.000184254 |
| ERO1B | 1.460500987 | 0.546463333 | 6.48548E-06 | 5.90831E-05 |
| ATPIF1 | 1.461675776 | 0.547623333 | 2.16698E-05 | 0.000165201 |
| CCDC71L | 1.463186159 | 0.549113333 | 1.64298E-11 | 1.65642E-09 |
| HMCN1 | 1.463777897 | 0.549696667 | 2.97139E-05 | 0.000215293 |
| SYNPO | 1.464224396 | 0.550136667 | 1.42713E-07 | 2.51417E-06 |
| CERCAM | 1.465737412 | 0.551626667 | 7.19673E-07 | 9.33889E-06 |
| SEPT2 | 1.467262162 | 0.553126667 | 2.52936E-13 | 6.76543E-11 |
| AGR2 | 1.468150636 | 0.554 | 8.57767E-13 | 1.72957E-10 |
| C6orf47 | 1.468812254 | 0.55465 | 7.08991E-06 | 6.37039E-05 |
| DNAJB5 | 1.469389292 | 0.555216667 | 2.36837E-08 | 5.84937E-07 |
| ZNF618 | 1.470866864 | 0.556666667 | 4.7817E-06 | 4.56671E-05 |
| GPC6 | 1.471614708 | 0.5574 | 6.26138E-09 | 2.00087E-07 |
| LOC105369239 | 1.47253984 | 0.558306667 | 8.22102E-07 | 1.04305E-05 |
| ABCC6P2 | 1.47253984 | 0.558306667 | 8.22102E-07 | 1.04305E-05 |
| ABCC6 | 1.47253984 | 0.558306667 | 8.22102E-07 | 1.04305E-05 |
| ABCC6P1 | 1.47253984 | 0.558306667 | 8.22102E-07 | 1.04305E-05 |
| PARP9 | 1.473118342 | 0.558873333 | 0.001283241 | 0.005346589 |
| PLEKHA2 | 1.476062012 | 0.561753333 | 2.89293E-06 | 2.99887E-05 |
| TNFAIP3 | 1.479756711 | 0.56536 | 4.73007E-08 | 1.02582E-06 |
| DAAM2 | 1.480522757 | 0.566106667 | 6.38265E-08 | 1.31186E-06 |
| ITPR1 | 1.481949891 | 0.567496667 | 1.11196E-07 | 2.04209E-06 |
| SIK2 | 1.482234113 | 0.567773333 | 4.95655E-09 | 1.67141E-07 |
| SCG5 | 1.483179628 | 0.568693333 | 1.02503E-12 | 2.00514E-10 |
| NAA15 | 1.487259781 | 0.572656667 | 1.09733E-09 | 4.76751E-08 |
| NABP1 | 1.48808816 | 0.57346 | 8.17552E-10 | 3.72916E-08 |
| HEG1 | 1.489030529 | 0.574373333 | 2.09142E-11 | 2.00079E-09 |
| ENPP2 | 1.491513118 | 0.576776667 | 1.41422E-05 | 0.000115126 |
| MFHAS1 | 1.492299042 | 0.577536667 | 2.84086E-09 | 1.0381E-07 |
| SPRYD4 | 1.493216477 | 0.578423333 | 3.8083E-09 | 1.32278E-07 |
| PIM2 | 1.494766359 | 0.57992 | 4.3449E-09 | 1.48039E-07 |
| NCOA7 | 1.495246492 | 0.580383333 | 1.85038E-07 | 3.08023E-06 |
| BDNF | 1.495571274 | 0.580696667 | 1.33191E-05 | 0.000109308 |
| OVOS | 1.498774496 | 0.583783333 | 5.9368E-06 | 5.48728E-05 |
| OVOS2 | 1.498774496 | 0.583783333 | 5.9368E-06 | 5.48728E-05 |
| PCDHB14 | 1.498843756 | 0.58385 | 2.0881E-06 | 2.27702E-05 |
| CXCL2 | 1.499893432 | 0.58486 | 7.2438E-07 | 9.39066E-06 |
| CXCL1 | 1.499893432 | 0.58486 | 7.2438E-07 | 9.39066E-06 |
| FKBP7 | 1.500271218 | 0.585223333 | 4.98629E-09 | 1.67856E-07 |
| STAT4 | 1.50318579 | 0.588023333 | 4.76977E-06 | 4.55643E-05 |
| PLAG1 | 1.508888987 | 0.593486667 | 1.15476E-08 | 3.25478E-07 |
| SEMA3C | 1.50934227 | 0.59392 | 2.65351E-08 | 6.44424E-07 |
| CHRNA9 | 1.51082511 | 0.595336667 | 2.78181E-07 | 4.28593E-06 |
| NFIA | 1.514333888 | 0.598683333 | 5.58732E-05 | 0.000369845 |
| SCARNA14 | 1.515467941 | 0.599763333 | 4.75814E-08 | 1.03021E-06 |
| ZNF155 | 1.515601003 | 0.59989 | 4.04058E-07 | 5.78766E-06 |
| TTC39A | 1.517335377 | 0.60154 | 8.7602E-06 | 7.63223E-05 |
| CFH | 1.51774561 | 0.60193 | 1.12311E-05 | 9.43378E-05 |
| SLC12A8 | 1.522765019 | 0.606693333 | 8.1303E-07 | 1.03321E-05 |
| FXN | 1.524377269 | 0.60822 | 1.07684E-11 | 1.22018E-09 |
| ARL14 | 1.524405445 | 0.608246667 | 1.1187E-05 | 9.40482E-05 |
| PTGES | 1.525071272 | 0.608876667 | 1.69618E-09 | 6.8262E-08 |
| VCAN | 1.525582289 | 0.60936 | 1.21498E-07 | 2.20146E-06 |
| EFEMP1 | 1.525716239 | 0.609486667 | 4.12621E-11 | 3.37997E-09 |
| CALD1 | 1.526816483 | 0.610526667 | 2.1845E-10 | 1.286E-08 |
| CSRNP1 | 1.52737749 | 0.611056667 | 8.15421E-11 | 5.80825E-09 |
| SDC1 | 1.527564538 | 0.611233333 | 6.7253E-11 | 4.9783E-09 |
| DMKN | 1.532669536 | 0.616046667 | 3.4032E-06 | 3.43986E-05 |
| ARID3B | 1.533721639 | 0.617036667 | 1.62395E-11 | 1.64992E-09 |
| RSPO3 | 1.536253893 | 0.619416667 | 0.000364253 | 0.0018247 |
| PLEKHH1 | 1.538225129 | 0.621266667 | 1.03282E-06 | 1.26233E-05 |
| GPX3 | 1.542196572 | 0.624986667 | 3.6276E-07 | 5.29253E-06 |
| NFKBIA | 1.543387151 | 0.6261 | 6.52195E-13 | 1.42465E-10 |
| MSRB3 | 1.544146892 | 0.62681 | 3.69942E-06 | 3.68433E-05 |
| METTL21A | 1.54768657 | 0.630113333 | 1.41196E-09 | 5.86859E-08 |
| CNN2 | 1.547840342 | 0.630256667 | 6.65343E-09 | 2.09537E-07 |
| ZNF267 | 1.548638056 | 0.631 | 1.85512E-08 | 4.79248E-07 |
| USP44 | 1.549579386 | 0.631876667 | 3.1563E-05 | 0.000226624 |
| AVPI1 | 1.55022039 | 0.632473333 | 3.7718E-10 | 1.99333E-08 |
| TPD52L1 | 1.550360085 | 0.632603333 | 4.39529E-09 | 1.49472E-07 |
| PTGER2 | 1.55139924 | 0.63357 | 1.75321E-08 | 4.58035E-07 |
| SMIM10 | 1.55148527 | 0.63365 | 8.65618E-07 | 1.08773E-05 |
| ARID5B | 1.553454515 | 0.63548 | 1.45773E-11 | 1.52437E-09 |
| TFRC | 1.557461679 | 0.639196667 | 8.92639E-16 | 8.35659E-13 |
| SERPINH1 | 1.557893559 | 0.639596667 | 1.75719E-13 | 4.91716E-11 |
| PLAC8 | 1.559802462 | 0.641363333 | 2.80432E-10 | 1.54214E-08 |
| KLF5 | 1.562356117 | 0.643723333 | 2.49233E-05 | 0.000185493 |
| C1orf53 | 1.563547809 | 0.644823333 | 9.26408E-12 | 1.09058E-09 |
| TET1 | 1.564657259 | 0.645846667 | 1.14489E-07 | 2.09376E-06 |
| PCIF1 | 1.565973718 | 0.64706 | 1.03719E-10 | 7.03126E-09 |
| EDARADD | 1.56612207 | 0.647196667 | 1.25872E-07 | 2.26818E-06 |
| CREB5 | 1.566310244 | 0.64737 | 3.51404E-06 | 3.53192E-05 |
| AMOTL2 | 1.570056707 | 0.650816667 | 1.46166E-11 | 1.52443E-09 |
| FOS | 1.571210708 | 0.651876667 | 1.21337E-11 | 1.32893E-09 |
| IL6 | 1.571483001 | 0.652126667 | 4.00122E-06 | 3.93999E-05 |
| ELOVL6 | 1.571896978 | 0.652506667 | 2.30977E-07 | 3.69029E-06 |
| MMP2 | 1.573281323 | 0.653776667 | 7.92077E-08 | 1.56422E-06 |
| TXNIP | 1.573899404 | 0.654343333 | 3.53175E-06 | 3.5479E-05 |
| CFHR1 | 1.57665827 | 0.65687 | 1.15265E-06 | 1.3788E-05 |
| AFAP1 | 1.579473063 | 0.659443333 | 2.66593E-11 | 2.41525E-09 |
| C17orf58 | 1.57956065 | 0.659523333 | 2.17943E-11 | 2.07489E-09 |
| APH1B | 1.580184848 | 0.660093333 | 1.01673E-07 | 1.90062E-06 |
| SLITRK5 | 1.580210406 | 0.660116667 | 9.64126E-07 | 1.18873E-05 |
| DUSP2 | 1.585059137 | 0.664536667 | 4.317E-07 | 6.10357E-06 |
| SNX16 | 1.586352443 | 0.665713333 | 6.78303E-07 | 8.86348E-06 |
| LETM1 | 1.588006334 | 0.667216667 | 5.36341E-09 | 1.7662E-07 |
| MYC | 1.588149434 | 0.667346667 | 2.97002E-13 | 7.73364E-11 |
| STARD4 | 1.599953526 | 0.67803 | 2.28623E-10 | 1.33048E-08 |
| TMEM150C | 1.601011126 | 0.678983333 | 3.38902E-06 | 3.42905E-05 |
| ALG5 | 1.603109902 | 0.680873333 | 6.09097E-15 | 3.4213E-12 |
| IFI44 | 1.603691531 | 0.681396667 | 1.05927E-08 | 3.01806E-07 |
| PALMD | 1.603943512 | 0.681623333 | 1.13622E-07 | 2.08275E-06 |
| RNF152 | 1.604125111 | 0.681786667 | 1.14309E-05 | 9.58728E-05 |
| EPDR1 | 1.604581053 | 0.682196667 | 7.31293E-10 | 3.38279E-08 |
| BICC1 | 1.605333825 | 0.682873333 | 1.95094E-07 | 3.20272E-06 |
| BASP1 | 1.606576858 | 0.68399 | 5.3533E-15 | 3.05084E-12 |
| TMTC3 | 1.60666966 | 0.684073333 | 1.12432E-09 | 4.8473E-08 |
| MAPK8 | 1.6112496 | 0.68818 | 3.77062E-05 | 0.000264745 |
| SNAP25 | 1.616249585 | 0.69265 | 4.38902E-07 | 6.18537E-06 |
| MFSD2A | 1.624932737 | 0.70038 | 4.49113E-09 | 1.52099E-07 |
| CNEP1R1 | 1.627694559 | 0.70283 | 1.78342E-07 | 2.99417E-06 |
| FICD | 1.627920221 | 0.70303 | 3.38128E-11 | 2.90281E-09 |
| PHF10 | 1.630796388 | 0.705576667 | 1.24934E-12 | 2.23286E-10 |
| PEX19 | 1.63221752 | 0.706833333 | 2.26685E-09 | 8.61993E-08 |
| PPP2R2D | 1.633504014 | 0.70797 | 4.37126E-11 | 3.52923E-09 |
| SRGN | 1.634802854 | 0.709116667 | 2.68864E-13 | 7.14289E-11 |
| VGLL3 | 1.635335525 | 0.709586667 | 6.23633E-07 | 8.26723E-06 |
| MLLT11 | 1.640163912 | 0.71384 | 1.01897E-11 | 1.17149E-09 |
| FAM117A | 1.642818767 | 0.716173333 | 3.10655E-10 | 1.68711E-08 |
| APP | 1.642868112 | 0.716216667 | 8.40026E-13 | 1.72026E-10 |
| P3H2 | 1.646375403 | 0.719293333 | 1.72238E-09 | 6.91751E-08 |
| RGS2 | 1.647886258 | 0.720616667 | 8.27077E-14 | 2.60159E-11 |
| HOXA1 | 1.649718648 | 0.72222 | 2.71616E-07 | 4.20128E-06 |
| PALLD | 1.656919295 | 0.728503333 | 2.82226E-10 | 1.54984E-08 |
| NREP | 1.660168872 | 0.73133 | 4.32019E-07 | 6.10589E-06 |
| RNF145 | 1.664608989 | 0.735183333 | 8.84359E-10 | 3.9785E-08 |
| GLT8D2 | 1.68281121 | 0.750873333 | 2.48007E-10 | 1.41529E-08 |
| GYG2 | 1.685620775 | 0.75328 | 4.16718E-08 | 9.26749E-07 |
| RAD23B | 1.686103776 | 0.753693333 | 8.95304E-12 | 1.06674E-09 |
| SNAI2 | 1.690707006 | 0.757626667 | 1.23751E-12 | 2.22181E-10 |
| RBM47 | 1.705431332 | 0.770136667 | 6.87434E-08 | 1.39398E-06 |
| EGR1 | 1.705577132 | 0.77026 | 3.97418E-15 | 2.62888E-12 |
| PPP2R2B | 1.712554618 | 0.77615 | 3.08973E-08 | 7.29203E-07 |
| ARL4A | 1.714355927 | 0.777666667 | 2.01311E-07 | 3.28438E-06 |
| SMOC1 | 1.719874546 | 0.782303333 | 2.38764E-08 | 5.88957E-07 |
| RPUSD4 | 1.722606683 | 0.784593333 | 7.15243E-10 | 3.32419E-08 |
| PRDM1 | 1.728423399 | 0.789456667 | 3.06307E-07 | 4.63219E-06 |
| HLA-F | 1.76210513 | 0.8173 | 5.15431E-08 | 1.09868E-06 |
| GATM | 1.765707861 | 0.820246667 | 3.77232E-06 | 3.74556E-05 |
| FOSL2 | 1.772435752 | 0.825733333 | 3.46201E-11 | 2.96564E-09 |
| MAPK9 | 1.772603663 | 0.82587 | 2.04978E-09 | 7.96395E-08 |
| PCDH7 | 1.77436564 | 0.827303333 | 2.06248E-08 | 5.21846E-07 |
| PTGFRN | 1.783539705 | 0.834743333 | 1.06835E-08 | 3.04173E-07 |
| CSGALNACT1 | 1.793979411 | 0.843163333 | 6.77625E-08 | 1.37835E-06 |
| KIAA1462 | 1.806336489 | 0.853066667 | 1.98249E-11 | 1.92944E-09 |
| ZNF165 | 1.821474334 | 0.865106667 | 1.59932E-08 | 4.2633E-07 |
| GRAMD3 | 1.831738215 | 0.873213333 | 3.34664E-07 | 4.96741E-06 |
| ANGPT1 | 1.846832894 | 0.885053333 | 1.43929E-06 | 1.67084E-05 |
| PPP1R3B | 1.851789357 | 0.88892 | 4.01114E-09 | 1.37983E-07 |
| LYPD6B | 1.859270484 | 0.894736667 | 9.17682E-09 | 2.7118E-07 |
| CEBPD | 1.859442325 | 0.89487 | 1.84699E-12 | 3.02176E-10 |
| MZT1 | 1.885242356 | 0.91475 | 4.52707E-15 | 2.87096E-12 |
| EGR3 | 1.889651252 | 0.91812 | 5.61931E-07 | 7.59525E-06 |
| DUSP1 | 1.891717509 | 0.919696667 | 1.21013E-11 | 1.32893E-09 |
| DNAJC22 | 1.910345168 | 0.933833333 | 5.8542E-08 | 1.22372E-06 |
| ID4 | 1.945786383 | 0.960353333 | 2.70595E-10 | 1.49852E-08 |
| GLIPR1 | 1.971107782 | 0.979006667 | 1.22697E-10 | 8.01381E-09 |
| EREG | 1.978280043 | 0.984246667 | 1.3275E-12 | 2.33434E-10 |
| CYR61 | 1.985909666 | 0.9898 | 2.57564E-18 | 1.26589E-14 |
| E2F6 | 2.003778907 | 1.002723333 | 5.17263E-08 | 1.10175E-06 |
| KYNU | 2.01884479 | 1.01353 | 2.67284E-09 | 9.88649E-08 |
| ID3 | 2.021211089 | 1.01522 | 2.04529E-15 | 1.51733E-12 |
| SGK1 | 2.029198501 | 1.02091 | 1.14491E-15 | 9.78621E-13 |
| MYL9 | 2.064067284 | 1.04549 | 1.11596E-14 | 5.62544E-12 |
| FABP4 | 2.081576625 | 1.057676667 | 6.58641E-10 | 3.10889E-08 |
| PTGS2 | 2.088127782 | 1.06221 | 1.47226E-14 | 6.65379E-12 |
| CTSZ | 2.131816607 | 1.092083333 | 5.3217E-15 | 3.05084E-12 |
| ID1 | 2.139153485 | 1.09704 | 7.13542E-17 | 1.16899E-13 |
| ARHGDIB | 2.152664269 | 1.106123333 | 1.40833E-14 | 6.43887E-12 |
| LIN28B | 2.181267441 | 1.125166667 | 1.58268E-10 | 9.89338E-09 |
| IL11 | 2.255848216 | 1.17367 | 3.14428E-14 | 1.23324E-11 |
| FRZB | 2.316954961 | 1.21223 | 6.41467E-10 | 3.04244E-08 |
| MPZL3 | 2.6102657 | 1.384196667 | 3.16658E-13 | 8.13769E-11 |
| CXCL8 | 2.881460015 | 1.5268 | 1.16744E-14 | 5.667E-12 |
| PDE4B | 3.281897087 | 1.71453 | 1.02994E-11 | 1.18064E-09 |
| CTGF | 3.453733265 | 1.788156667 | 9.39442E-20 | 9.9032E-16 |
