# Supplementary material for: POLE2 promotes osteosarcoma progression by enhancing the stability of CD44
Source: Cell Death Discov. 2024 Apr 16;10:177. doi: 10.1038/s41420-024-01875-x (PMC11021398; doi:10.1038/s41420-024-01875-x)
Supplement: Supplementary file 8 — Original Data File [file 41420_2024_1875_MOESM8_ESM.pdf]

Fig.4F

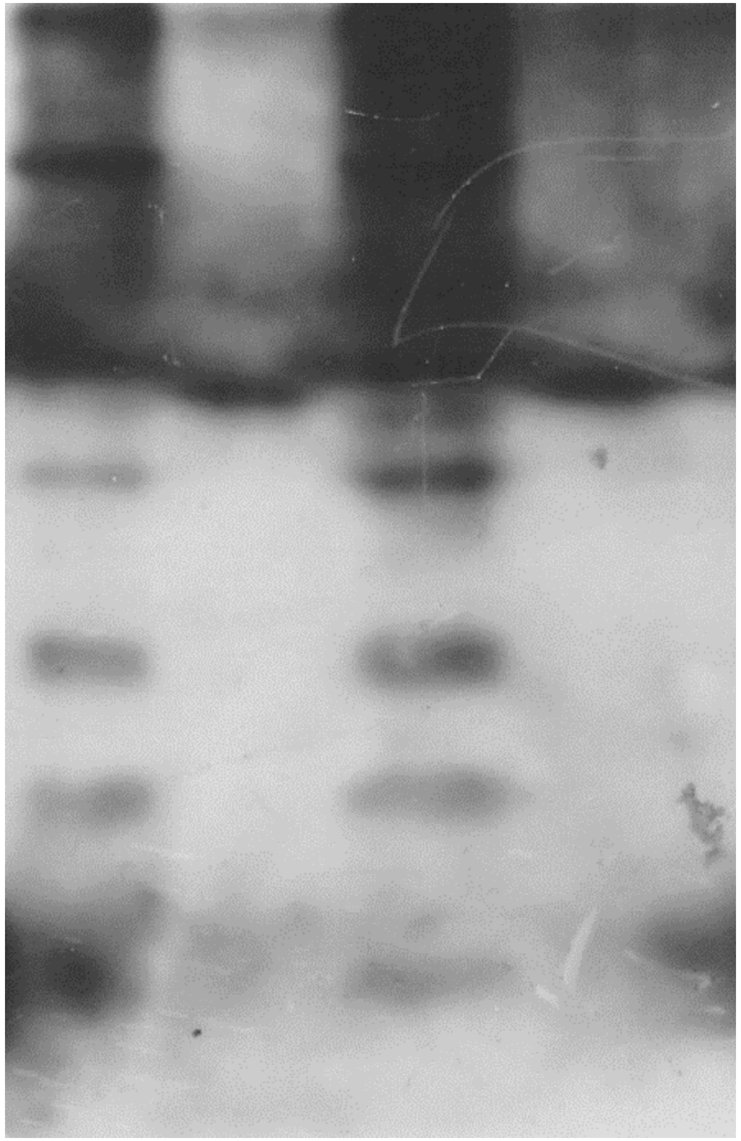

Fig.4K-Control and MDM2

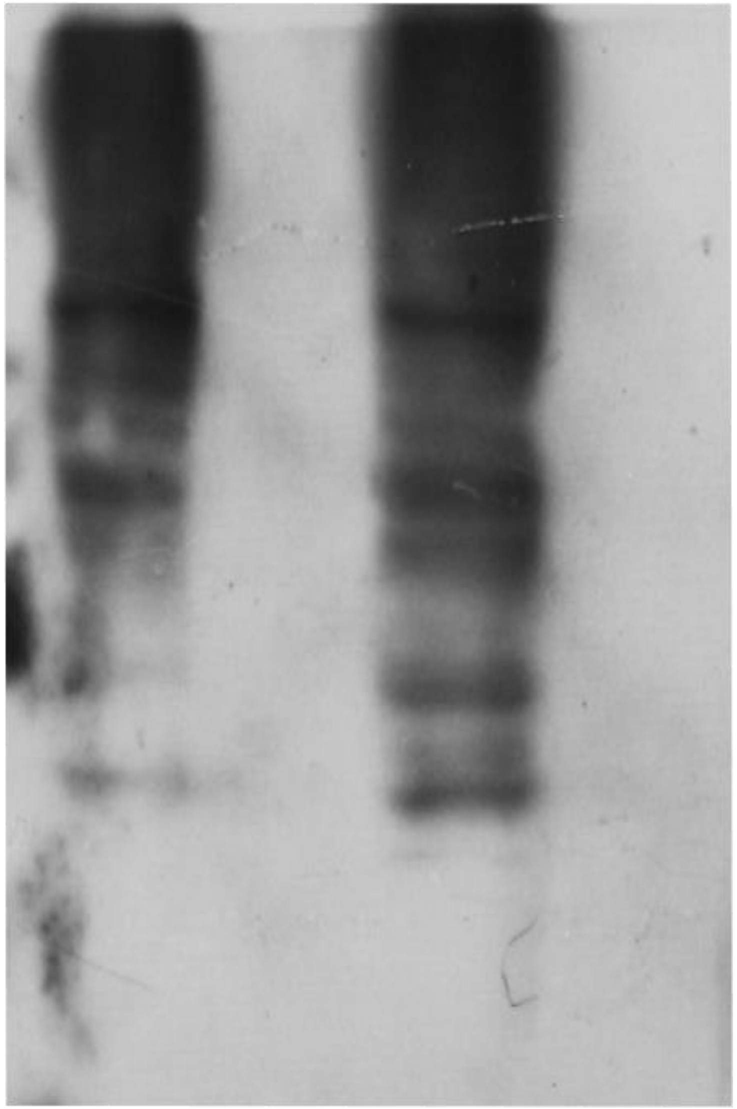

Fig.4K-shCtrl and shPOLE2+shMDM2

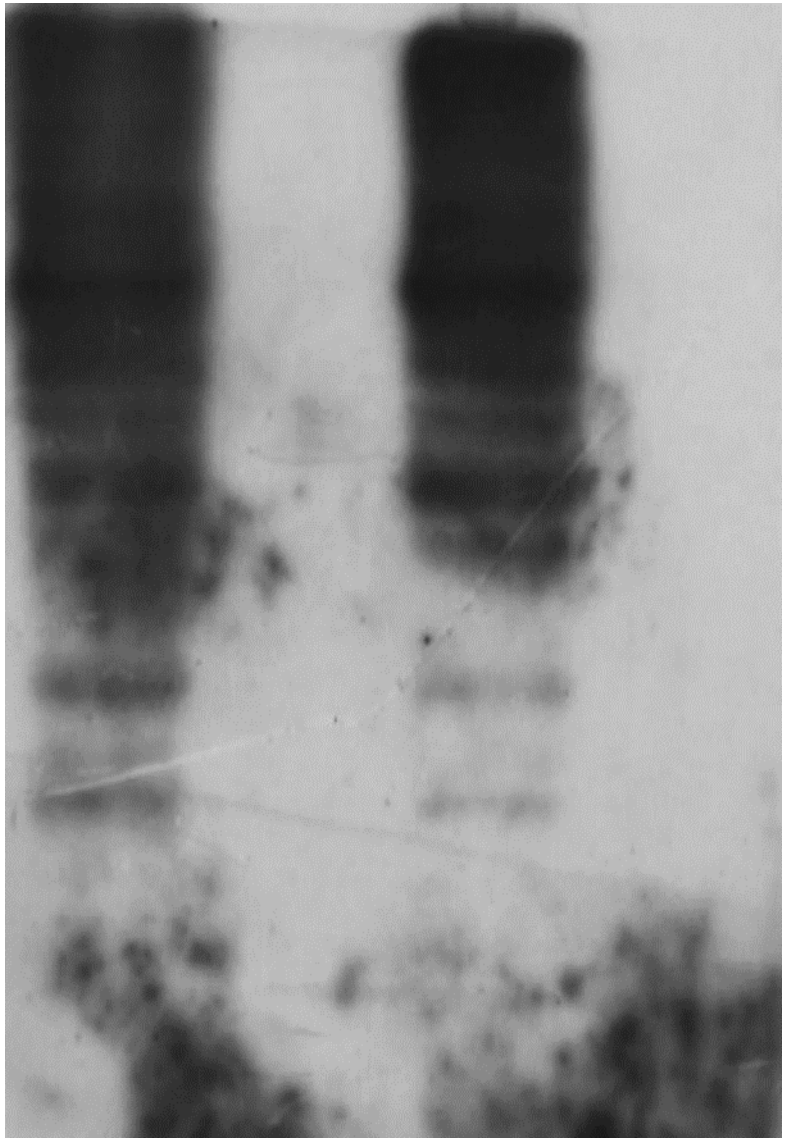

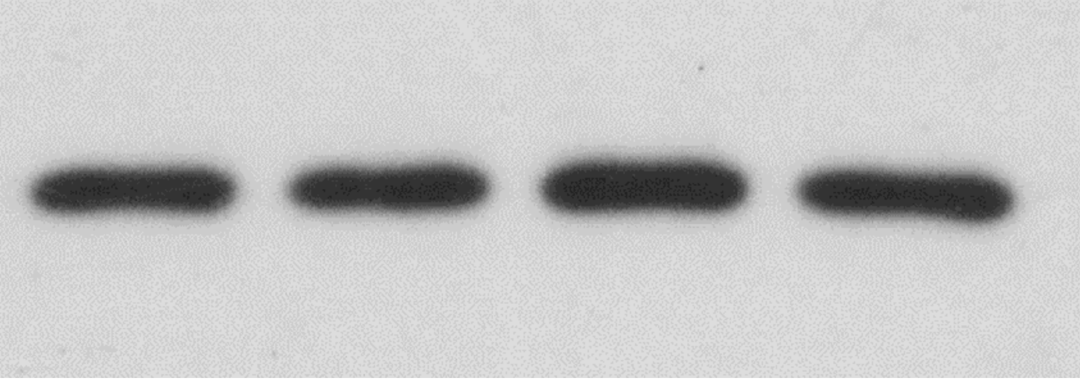

Fig4D-shCtrl-CD44

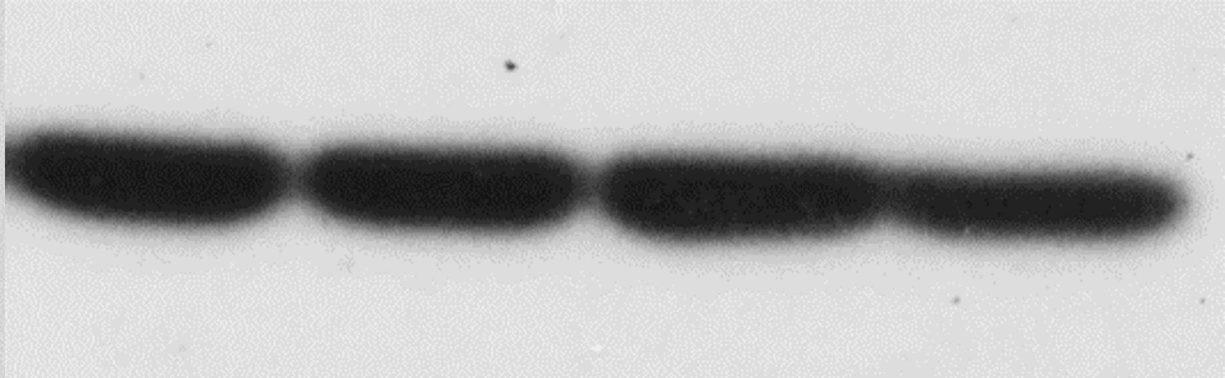

Fig4D-shCtrl-GAPDH

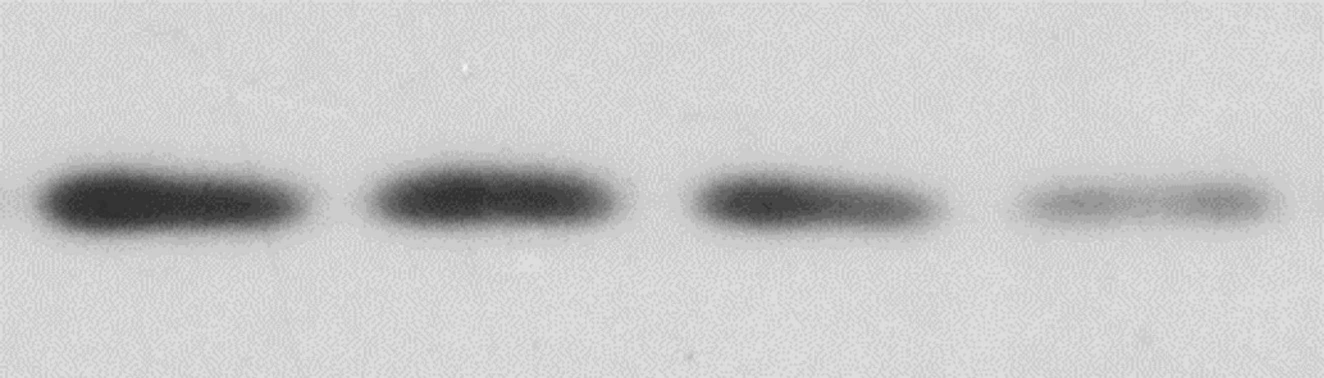

Fig4D-shPOLE2-CD44

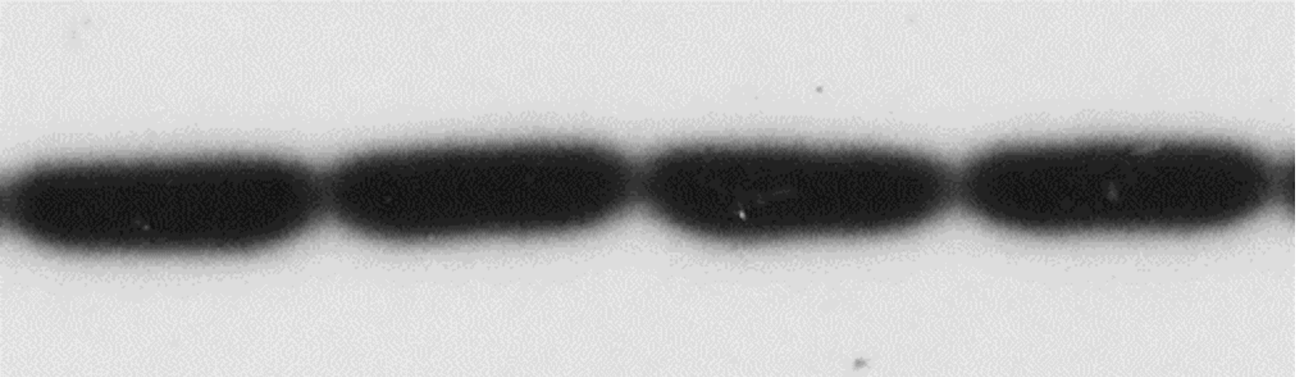

Fig4D-shPOLE2-GAPDH

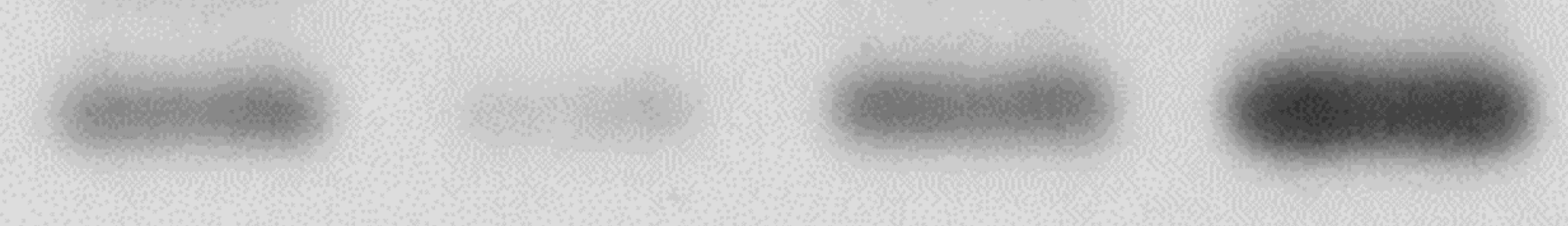

Fig4E-CD44

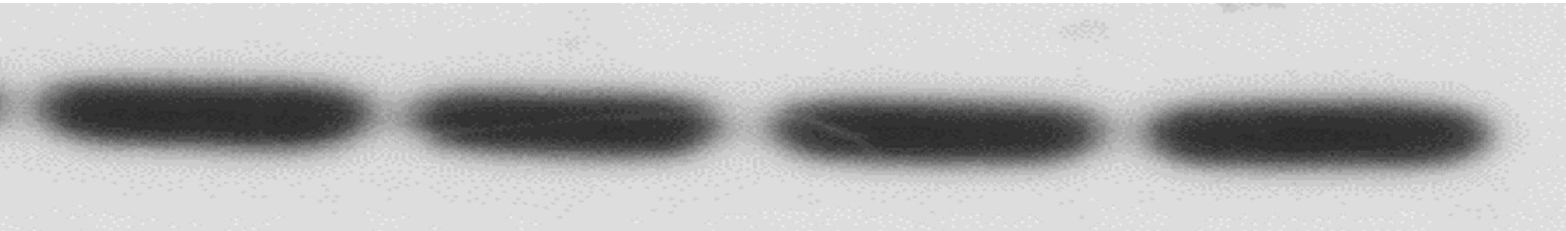

Fig4E-GAPDH

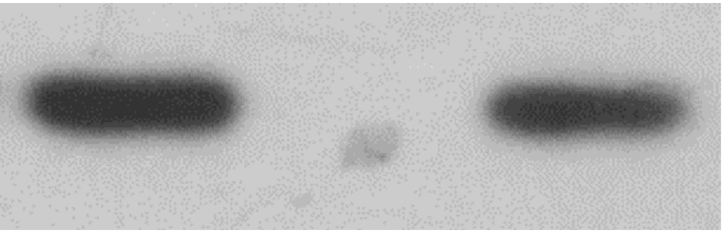

Fig4H-IB-MDM2

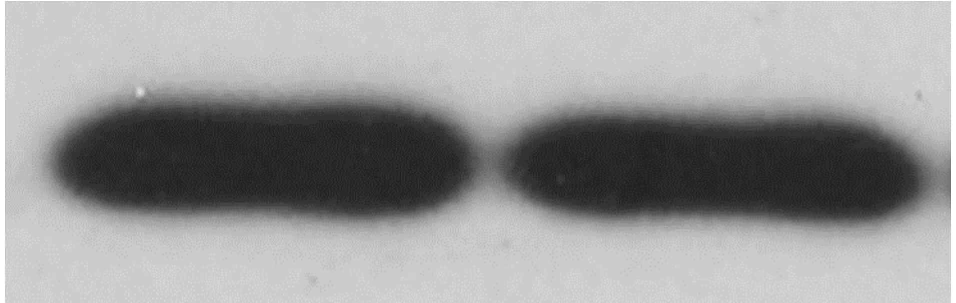

Fig4I-CD44

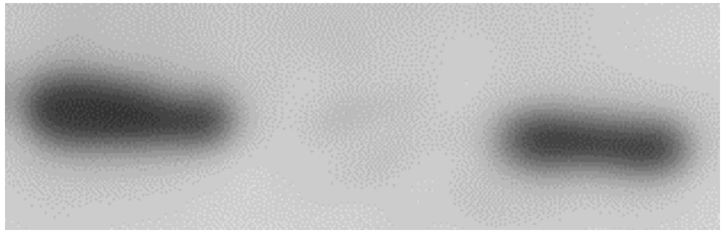

Fig4H-IB-POLE2

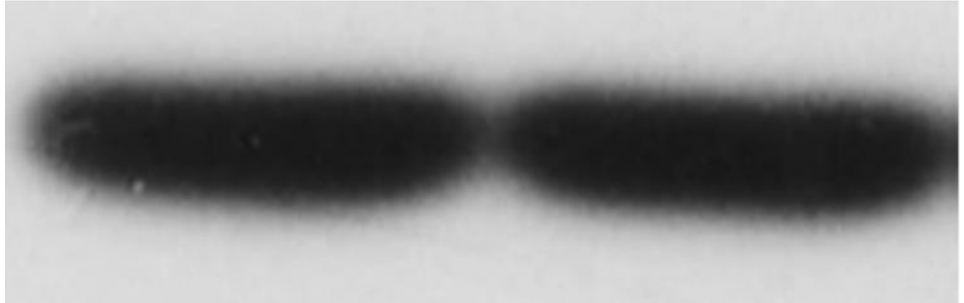

Fig4I-GAPDH

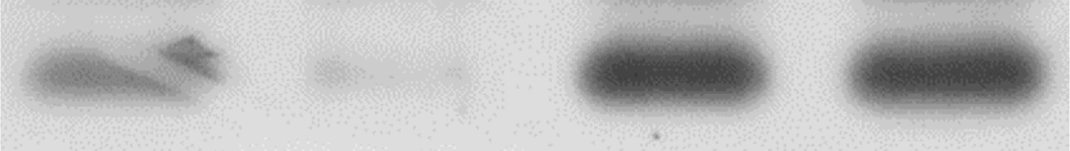

Fig4I-CD44

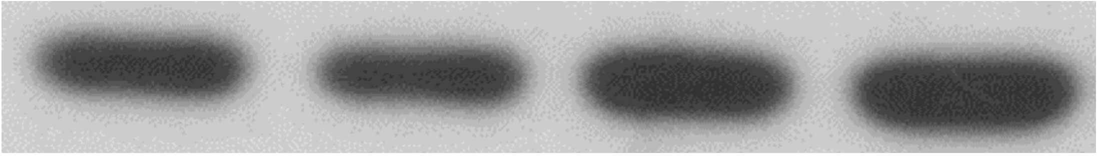

Fig4I-GAPDH

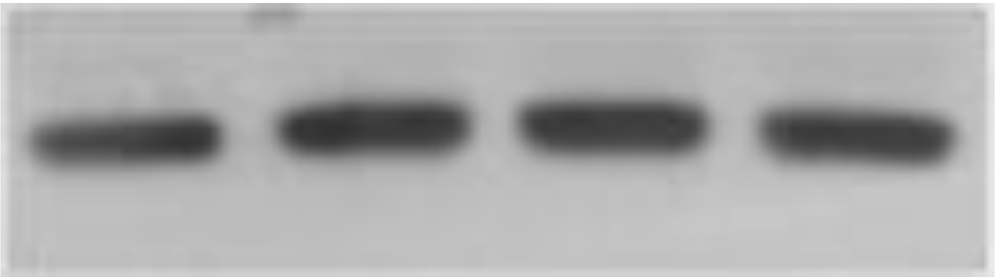

Fig4J-Control-CD44

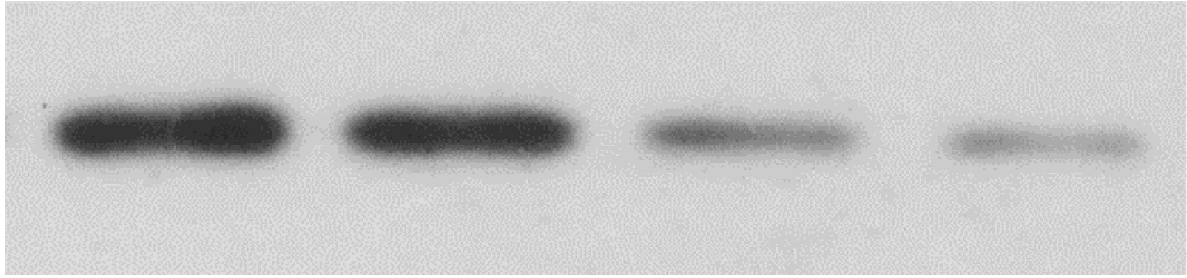

Fig4J-MDM2-CD44

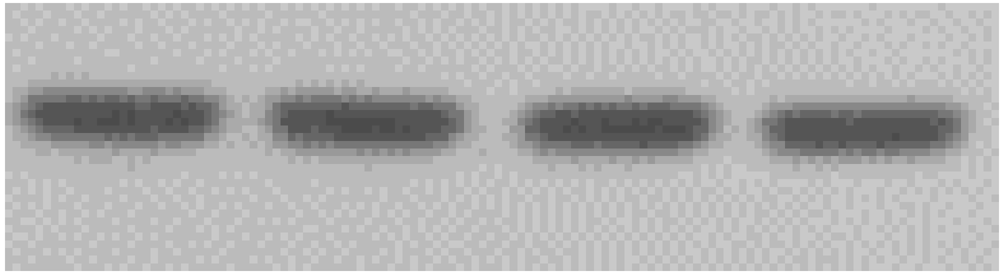

Fig4J-Control-GAPDH

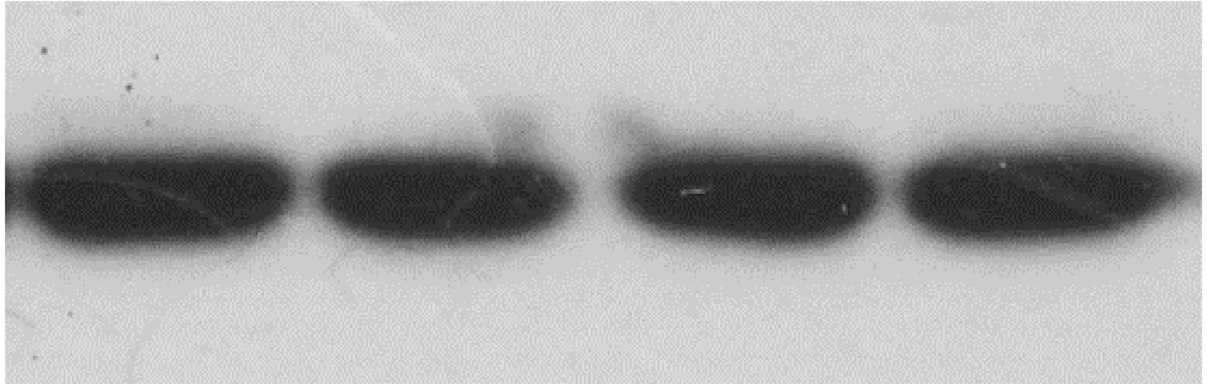

Fig4J-MDM2-GAPDH

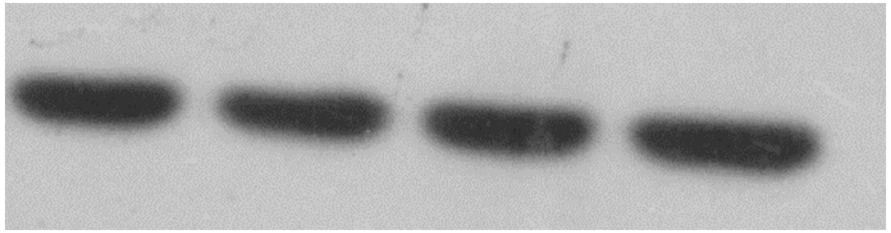

Fig4J-shCtrl-CD44

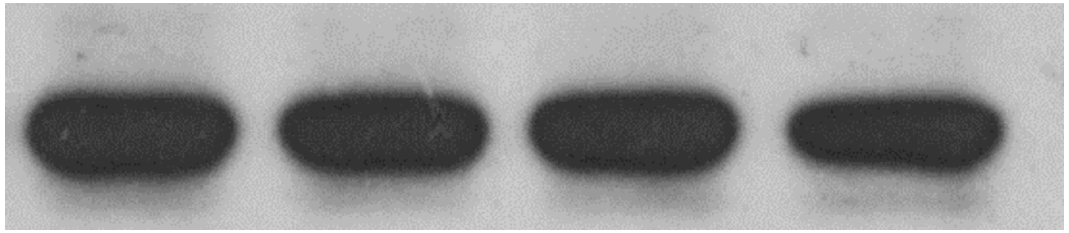

Fig4J-shCtrl-GAPDH

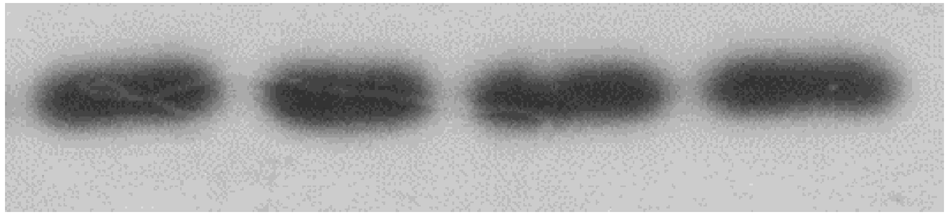

Fig4J-shPOLE2+shMDM2-CD44

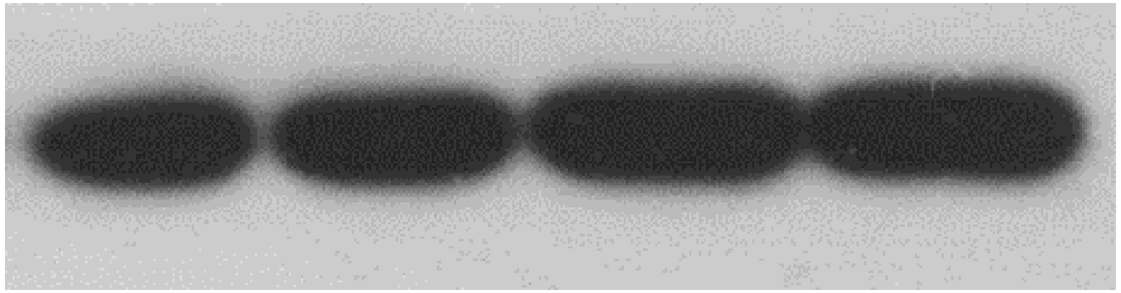

Fig4J-shPOLE2+shMDM2-GAPDH

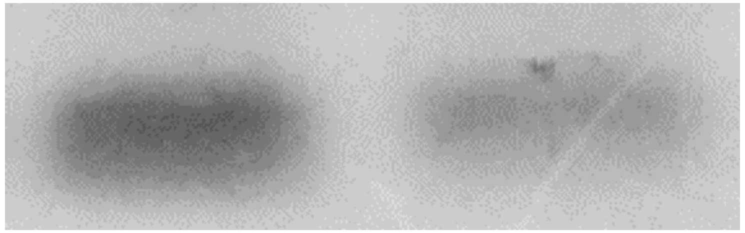

Fig5F-CD44

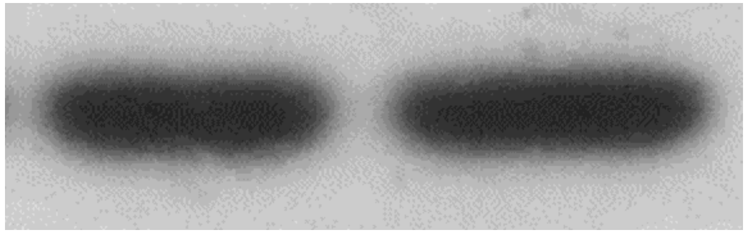

Fig5F-GAPDH

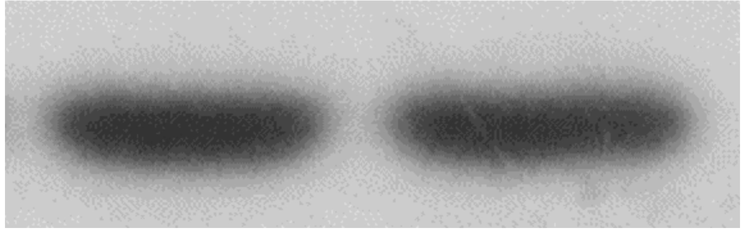

Fig5F-MDM2

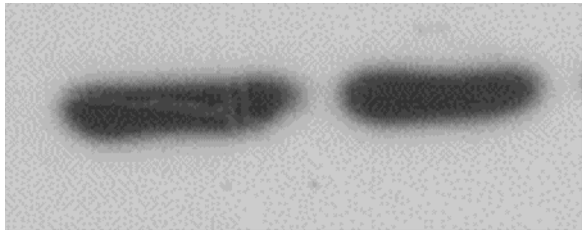

Fig.S1D-MNNG HOS-GAPDH

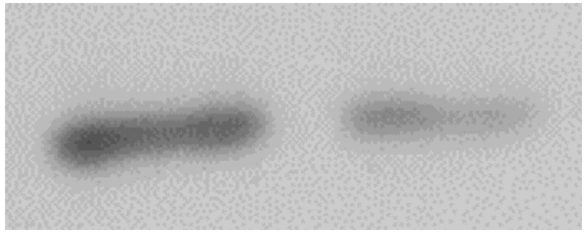

Fig.S1D-MNNG HOS-POLE2

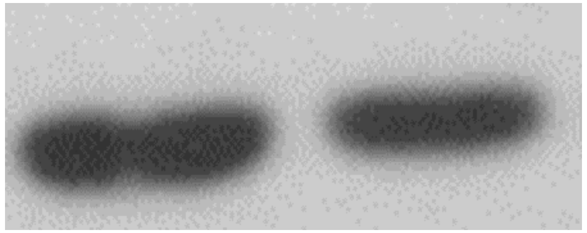

Fig.S1D-U-2OS-GAPDH

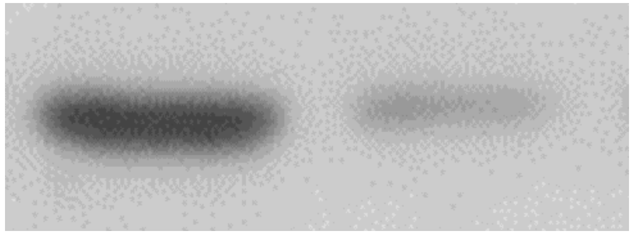

Fig.S1D-U-2OS-POLE2

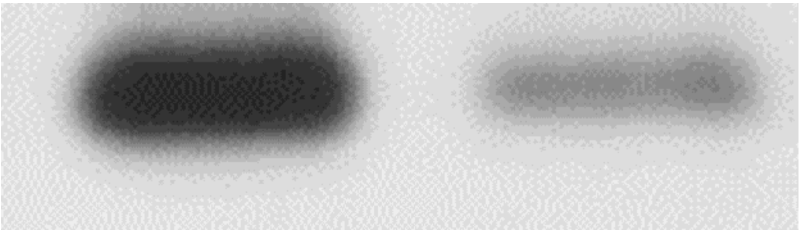

Fig.S3C-CD44

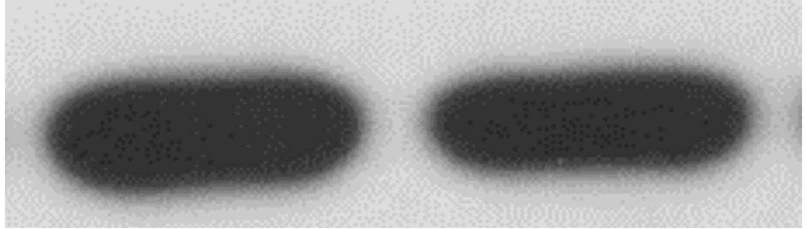

Fig.S3C-GAPDH

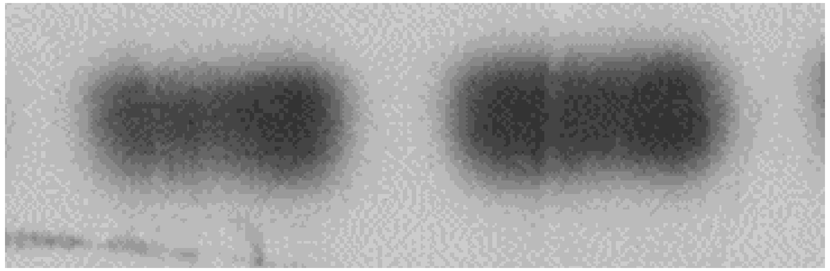

Fig.S3E-Akt

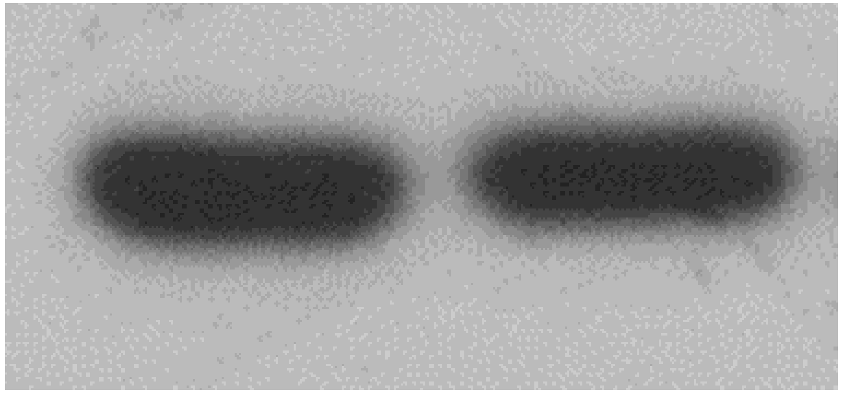

Fig.S3E-GAPDH

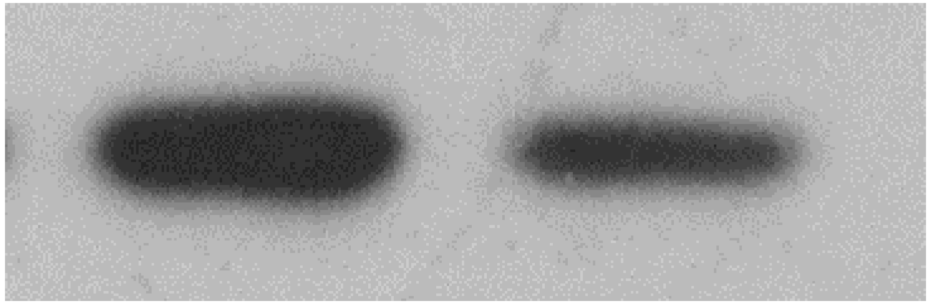

Fig.S3E-JNK1-2

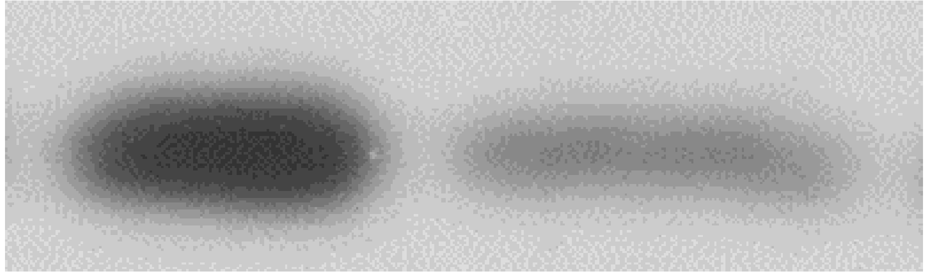

Fig.S3E-p-Akt

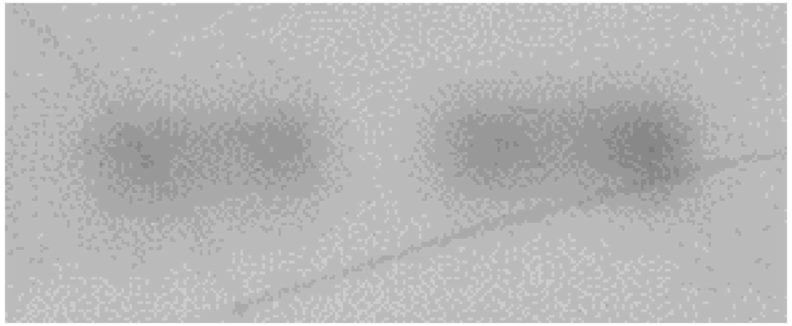

Fig.S3E-PI3K

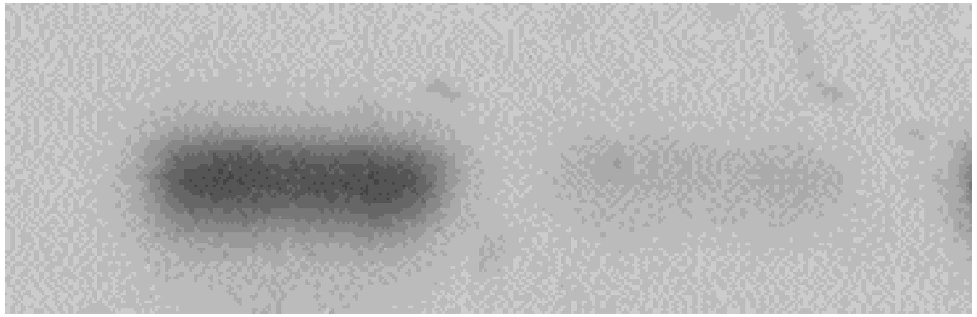

Fig.S3E-p-PI3K

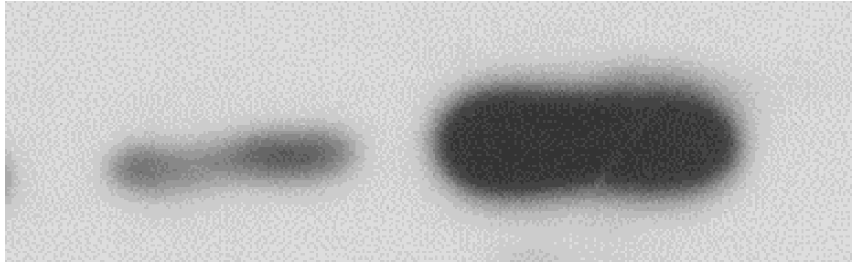

Fig.S3E-p-JNK1-2

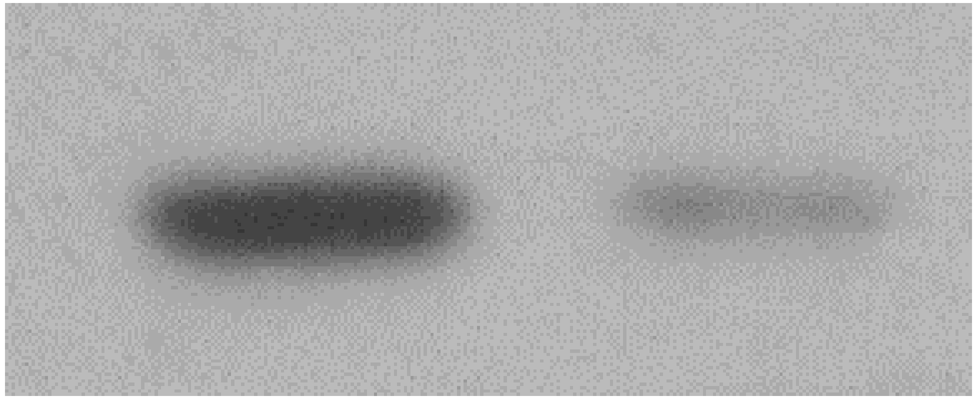

Fig.S3E-p-RAC

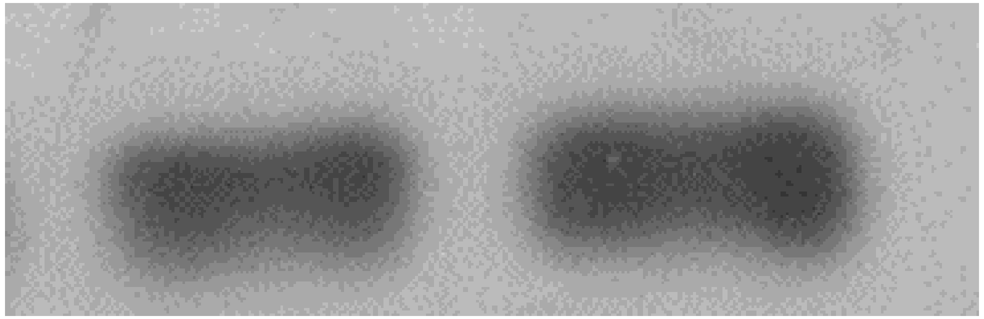

Fig.S3E-RAC

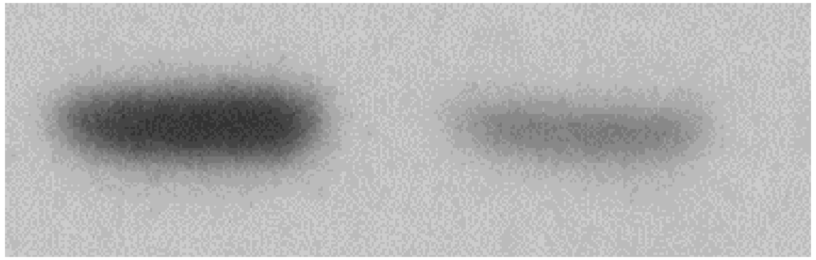

Fig.S5C-CD44

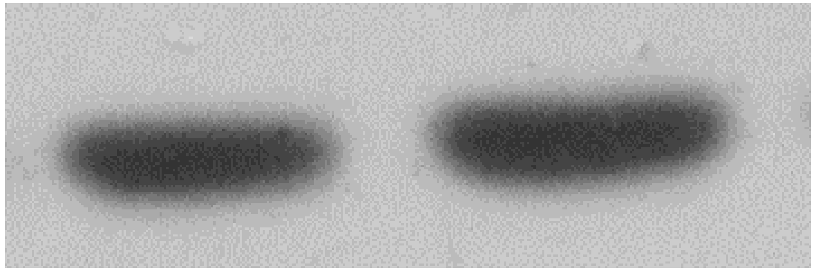

Fig.S5C-CD44-GAPDH

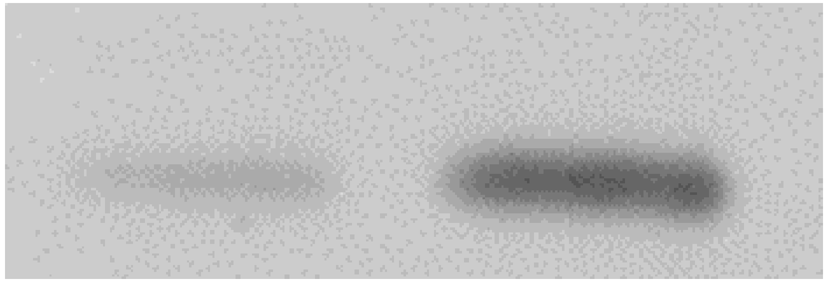

Fig.S5C-POLE2

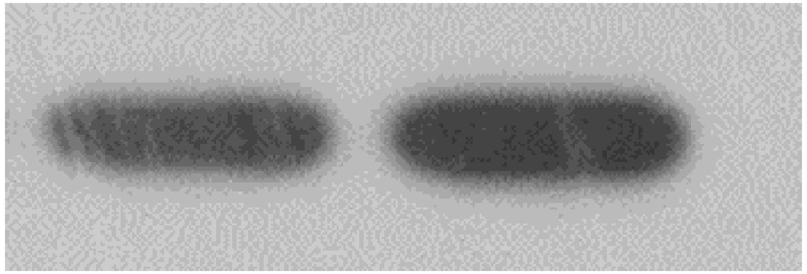

Fig.S5C-POLE2-GAPDH
